# Inferring Long-Term Effective Population Size with Mutation–Selection Models

**DOI:** 10.1093/molbev/msab160

**Published:** 2021-06-30

**Authors:** Thibault Latrille, Vincent Lanore, Nicolas Lartillot

**Affiliations:** 1 Laboratoire de Biométrie et Biologie Évolutive UMR 5558, Université de Lyon, Université Lyon 1, CNRS, Villeurbanne, France; 2 École Normale Supérieure de Lyon, Université de Lyon, Université Lyon 1, Lyon, France

**Keywords:** phylogenetic, codon models, mutation–selection models, population genetic, population size, mutation rate, life-history traits

## Abstract

Mutation–selection phylogenetic codon models are grounded on population genetics first principles and represent a principled approach for investigating the intricate interplay between mutation, selection, and drift. In their current form, mutation–selection codon models are entirely characterized by the collection of site-specific amino-acid fitness profiles. However, thus far, they have relied on the assumption of a constant genetic drift, translating into a unique effective population size ($N_{e}$) across the phylogeny, clearly an unrealistic assumption. This assumption can be alleviated by introducing variation in $N_{e}$ between lineages. In addition to $N_{e}$, the mutation rate (*μ*) is susceptible to vary between lineages, and both should covary with life-history traits (LHTs). This suggests that the model should more globally account for the joint evolutionary process followed by all of these lineage-specific variables ($N_{e}$, *μ*, and LHTs). In this direction, we introduce an extended mutation–selection model jointly reconstructing in a Bayesian Monte Carlo framework the fitness landscape across sites and long-term trends in $N_{e}$, *μ*, and LHTs along the phylogeny, from an alignment of DNA coding sequences and a matrix of observed LHTs in extant species. The model was tested against simulated data and applied to empirical data in mammals, isopods, and primates. The reconstructed history of $N_{e}$ in these groups appears to correlate with LHTs or ecological variables in a way that suggests that the reconstruction is reasonable, at least in its global trends. On the other hand, the range of variation in $N_{e}$ inferred across species is surprisingly narrow. This last point suggests that some of the assumptions of the model, in particular concerning the assumed absence of epistatic interactions between sites, are potentially problematic.

## Introduction

Since the realization by Zuckerkandl and Pauling (1965) that genetic sequences are informative about the evolutionary history of the species, molecular phylogenetics has developed into a mature and very active field. A broad array of models and inference methods have been developed, using DNA sequences for reconstructing the phylogenetic relationships among species (Felsenstein 1981), for estimating divergence times (Thorne and Kishino 2002), or for reconstructing the genetic sequences of remote ancestors (Liberles 2007). However, genetic sequences might contain information about other aspects of the evolutionary history and, in particular, about past population-genetic regimes.

Interspecific divergence is the long-term outcome of population-genetic processes, in which point mutations at the level of individuals are then subjected to selection and genetic drift, leading to substitutions at the level of the population. As a result, the substitution patterns that can be reconstructed along phylogenies are modulated by the underlying population-genetic parameters (mutation biases, selective landscapes, effective population size), suggesting the possibility to infer the past variation of these parameters over the phylogeny. Independently, ecological properties, such as phenotypic characters or life-history traits (LHTs) can be observed in extinct or in present-day species. Using the comparative method (Felsenstein 1985), these traits can be reconstructed for the unobserved ancestral species. Combined together, genetic and phenotypic ancestral reconstructions can then be used to unravel the interplay between evolutionary and ecological mechanisms.

Practically, in order to disentangle mutation, selection and genetic drift, we need to classify individual substitutions into different categories, differing in the strength of mutation, selection or genetic drift. In protein-coding DNA sequences, the mutational process occurs at the nucleotide level. Assuming that synonymous mutations are selectively neutral and that selection mostly acts at the protein level, synonymous substitutions can be used to infer the patterns of mutation, without any interference contributed by selection. Then, by comparing the nonsynonymous substitution rate relative to the synonymous substitution rate (the ratio $d_{N}/d_{S}$), one can estimate the global strength of selection acting on proteins. This idea was formalized using phylogenetic codon models (Goldman and Yang 1994; Muse and Gaut 1994). This led to a broad range of applications, either to detect proteins under adaptive selection (Kosiol et al. 2008), or to measure the modulations of the strength of purifying selection between sites (Echave et al. 2016), genes (Zhang and Yang 2015), or lineages (Lartillot and Poujol 2011).

Concerning variation in $d_{N}/d_{S}$ between lineages, and in a context mostly characterized by purifying selection, the nearly neutral theory predicts that changes in the global strength of selection (measured as $d_{N}/d_{S}$) is related to changes in the relative strength of genetic drift, which is in turn mediated by changes in effective population size ($N_{e}$) (Ohta 1992). Mechanistically, populations with high $N_{e}$ are characterized by more efficient purifying selection against mildly deleterious mutations, resulting in lower $d_{N}/d_{S}$ (Kimura 1979; Welch et al. 2008).

Codon models allowing for variation in $d_{N}/d_{S}$ across branches (Yang and Nielsen 1998; Yang 1998, 2007; Dutheil et al. 2012) have been used to empirically measure such changes in the efficacy of purifying selection along phylogenies. Alternatively, $d_{N}/d_{S}$ can be modeled as a continuous trait, varying along the phylogeny as a stochastic process, splitting at each node of the tree into independent processes (Seo et al. 2004). Once empirical estimates of the variation in $d_{N}/d_{S}$ between lineages or groups have been obtained, these can be compared with changes in $N_{e}$ across lineages, so as to test the validity of the predictions of the nearly neutral theory. Independent empirical estimation of $N_{e}$ is usually done vie proxies, such as the neutral diversity within species (Galtier 2016), or LHTs. For instance, animal species characterized by a large body size or an extended longevity are typically expected to also have a low $N_{e}$ (Romiguier et al. 2014). Alternatively, a Bayesian integrative framework has been proposed (Lartillot and Poujol 2011), extending the approach of Seo et al. (2004), in which the joint variation in *d_S_*, $d_{N}/d_{S}$ and in LHTs or other proxies of $N_{e}$ is modeled as a multivariate Brownian process, with a variance–covariance matrix capturing the signal of their correlated evolution.

Analyses using these approaches and these proxies of $N_{e}$ have suggested a negative correlation between $d_{N}/d_{S}$ and $N_{e}$ (Popadin et al. 2007; Lanfear et al. 2010; Lartillot and Poujol 2011; Lartillot and Delsuc 2012; Romiguier et al. 2014; Figuet et al. 2017), thus confirming the theoretical prediction of the nearly neutral theory. However, the universality and robustness of the correlation between $d_{N}/d_{S}$ and $N_{e}$ is still debated (Nabholz et al. 2013; Lanfear et al. 2014; Figuet et al. 2016; Bol**í**var et al. 2019), and further investigation might be required. Moreover, these analyses do not explicitly formalize the quantitative relationship between $N_{e}$ and $d_{N}/d_{S}$. This relation is in principle dependent on the underlying fitness landscape (Cherry 1998; Welch et al. 2008; Goldstein 2011), and can show complicated behavior due to nonequilibrium properties (Jones et al. 2017). These questions could be addressed in the context of a mechanistic modeling approach.

A first attempt in this direction was proposed by Nielsen and Yang (2003), using a population-genetic argument to relate the distribution of $d_{N}/d_{S}$ across sites with the underlying distribution of fitness effects. This first approach assumes that all nonsynonymous mutations at a given site have the same selection coefficient. As a result of this assumption, there is a simple, one-to-one mapping between the $d_{N}/d_{S}$ at a given site and the selection coefficient associated with all nonsynonymous mutations at that site. In practice, different nonsynonymous mutations are likely to have different fitness effects. In this direction, an alternative mutation–selection codon modeling approach originally proposed by Halpern and Bruno (1998) explicitly assigns a fitness parameter to each amino acid. As a result, the substitution rate between each pair of codons can be predicted, as the product of the mutation rate and the fixation probability of the new codon, which is in turn dependent on the fitness of the initial and the final codons. Since the strength of selection is typically not homogeneous along the protein sequence, and depends on the local physicochemical requirements (Echave et al. 2016; Goldstein and Pollock 2016, 2017), local changes in selective strength are usually taken into account by allowing for site-specific amino-acid fitness profiles. Site-specific amino-acid preferences are typically estimated either by penalized maximum likelihood (Tamuri et al. 2012, 2014), or in a Bayesian context, using an infinite mixture based on a Dirichlet process prior (Rodrigue et al. 2010; Rodrigue and Lartillot 2014). This second approach is further considered below.

Although not directly expressed in terms of this variable, the mutation–selection formalism induces an equilibrium $d_{N}/d_{S}$, which is theoretically lower than 1, thus explicitly modeling purifying selection (Dos Reis 2015; Spielman and Wilke 2015). As a result, the mutation–selection codon framework proved to be a valuable null (nearly neutral) model, against which to compare the observed $d_{N}/d_{S}$ by classical codon models, so as to test for the presence of adaptation (Rodrigue and Lartillot 2017; Bloom 2017).

However, these mutation–selection methods have so far assumed the strength of genetic drift, or equivalently $N_{e}$, to be constant across the phylogeny. This assumption is clearly not realistic, as attested by the empirically measured variation in $d_{N}/d_{S}$ between lineages using classical codon models or, more directly, by the broad range of synonymous neutral diversity observed across species (Galtier 2016). The impact of this assumption on the estimation of the fitness landscape across sites (Rodrigue and Lartillot 2014; Tamuri et al. 2014), or on the tests for the presence of adaptation (Rodrigue and Lartillot 2017; Bloom 2017) is totally unknown. Relaxing this assumption of a constant $N_{e}$ is thus necessary.

Conversely, since the mutation–selection formalism explicitly incorporates $N_{e}$ as a parameter of the model, extending the model so as to let $N_{e}$ vary across lineages is relatively straightforward, at least conceptually. This idea was previously explored in the context of two mechanistic models, relying on the distribution of $d_{N}/d_{S}$ across sites (Nielsen and Yang 2003) or accounting for selection on codon usage (Nielsen et al. 2007). Doing this in the context of mutation–selection models with site-specific amino-acid preferences would provide an occasion to address several important questions: Do we have enough signal in empirical sequence alignments, to estimate the evolutionary history of $N_{e}$ along a phylogeny? Can we more generally revisit the question of the empirical correlations between $N_{e}$ and ecological LHTs (longevity, maturity, weight, size, …), previously explored using classical $d_{N}/d_{S}$ based models, but now in the context of this mechanistic framework*?*

## New Approaches

**Fig. 1.**
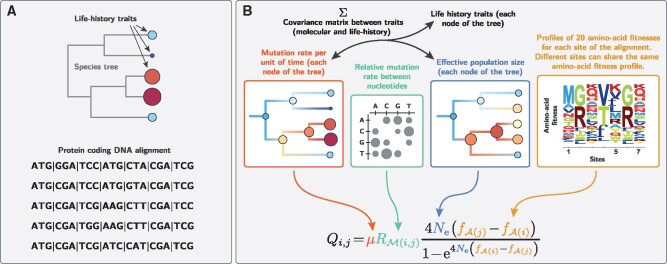
Model summary. (*A*) Our method requires a (given) rooted tree topology, an alignment of protein-coding DNA and (optionally) quantitative life-history trait for the extant species. (*B*) Relying on a codon model based on the mutation–selection formalism, assuming an autocorrelated geometric Brownian process for the variation through time in effective population size ($N_{e}$), mutation rate (***μ***), and life-history traits, our Bayesian inference method estimates amino-acid fitness profiles across sites, variation in mutation rate and effective population size along the tree, as well as the node ages and the nucleotide mutation rates.

To address these questions, here we introduce a variant of the mutation–selection codon model, in which selection is modulated along the sequence (using site-specific amino-acid profiles), whereas the mutation rate (*μ*), the effective population size ($N_{e}$), and LHTs are allowed to vary along the phylogeny (fig. 1). Methodologically, our model is fundamentally an integration between the Bayesian nonparametric version of the Halpern and Bruno (1998) mutation–selection model (Rodrigue and Lartillot 2014), and the molecular comparative framework modeling the joint evolution of life-history and molecular traits (Lartillot and Poujol 2011).

Formally, the substitution rate (per unit of time) from codon *i* to *j*, denoted $Q_{i,j}$, is equal to the total rate of mutation (per unit of time) at the level of the population ($2N_{e}\mu_{i,j}$) multiplied by the probability of fixation of the mutation $P_{\text{fix}}(i,j)$:

(1) $$Q_{i,j}=2N_{e}\mu_{i,j}P_{\text{fix}}(i,j).$$

In the case of synonymous mutations, which we assumed are neutral, the probability of fixation is independent of the original and target codon, and equals $1/2N_{e}$, such that $Q_{i,j}$ simplifies to:

(2) $$Q_{i,j}=\mu_{i,j}.$$

In the case of nonsynonymous mutations, the probability of fixation depends on the difference in fitness between the amino acid encoded by the initial and final codons:

(3) $$Q_{i,j}=\mu_{i,j}\frac{4N_{e}\left(f_{A(j)}-f_{A(i)}\right)}{1-e^{4N_{e}\left(f_{A(i)}-f_{A(j)}\right)}},$$

where ***f*** is a 20-dimensional vector specifying the log-fitness for each amino acid, and A(i) is the amino acid encoded by codon *i*.

In the model introduced here, $N_{e}$ and *μ* are allowed to vary between species (among branches) as a multivariate geometric Brownian process, but are assumed constant along the DNA sequence. Conversely, amino-acid fitness profiles ***f*** are considered constant along the tree but are assumed to vary across sites, being modeled as independent and identically distributed random-effects from an unknown distribution estimated using a Dirichlet process prior. Of note, since $N_{e}$ and ***f*** are confounded parameters (eq. 3), the effective population size at the root is set to 1 for identifiability of the fitness profiles. As a result, all values of $N_{e}$ along the phylogeny are relative to that of the root, with a value of $N_{e}>1$ reflecting an increase in $N_{e}$ along the branches (respectively a decrease for $N_{e}<1$) compared with the $N_{e}$ at the root.

This model was implemented in a Markov Chain Monte Carlo (MCMC) framework, allowing for joint inference of site-specific selection profiles and reconstruction of LHTs and population-genetic regimes along the phylogeny. After validating our model and our inference framework against simulated data, we apply it to several cases of interest across metazoans (placental mammals, primates and isopods), for which some proxies of $N_{e}$ are available.

## Results

### Validation Using Simulations

The inference framework was first tested on independently simulated multiple sequence alignments (MSAs) (see Materials and Methods). With the aim of applying the inference method to empirical data sets, the simulation parameters were chosen so as to match an empirically relevant empirical regime. Thus, the tree topology and the branch lengths were chosen based on a tree estimated on the mammalian data set further considered below. The other aspects of the simulation model (fitness landscape, variation in $N_{e}$) were then varied along a gradient of increasing complexity, so as to test the inference framework under increasingly challenging conditions.

**Fig. 2.**
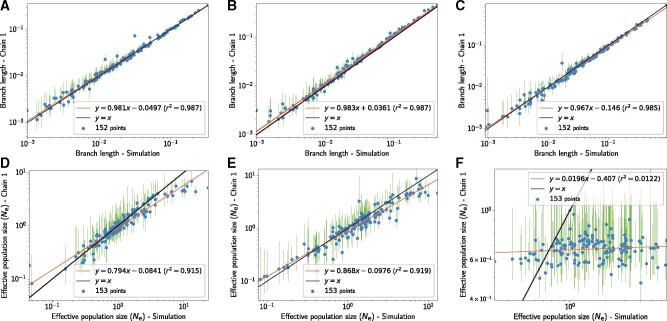
(*A*–*C*) Branch lengths in expected number of substitutions per site. (*D*–*F*) $N_{e}$ values across nodes (including the leaves) relative to $N_{e}$ at the root. From left to right: Simulation under the mutation–selection approximation (*A*, *D*), under a Wright–Fisher model accounting for small population size effects (5,000 individuals at the root), site linkage and short term fluctuation of $N_{e}$ (*B*, *E*), and finally accounting for site epistasis in the context of selection for protein stability (*C*, *F*). The tree root is 150 My old, where the initial population starts with a mutation rate of $10^{-8}$ per site per generation and generation time of 10 years. These experiments confirm that signal in the placental mammalian tree can allow to reliably infer the direction of change in $N_{e}$, even if linkage disequilibrium, short term fluctuation of $N_{e}$ and finite population size effects are not accounted for in the inference framework. However, the presence of epistasis between sites is a serious threat to the inference of $N_{e}$.

A first series of simulations was meant to test the soundness of our inference framework, by simulating essentially under the model used for inference, although with an independently developed software. Thus, the mutation–selection approximation was assumed to be valid, and sites were simulated under different fitness profiles empirically determined (Bloom 2017), and finally, $N_{e}$ was assumed to undergo discrete shifts at the tree nodes but otherwise to remain constant along each branch. In this context, branch lengths and branch-specific values of $N_{e}$ were accurately estimated by our inference method (fig. 2*A* and *D*). Concerning $N_{e}$, the slope of the linear regression between true and estimated branch-specific $N_{e}$ is 0.794 ($r^{2}=0.915$).

However, the assumptions made for this first round of simulations are almost certainly violated in practice. First, $N_{e}$ is expected to undergo continuous changes along the lineages of the phylogeny. Second, the diffusion approximation for the probability of fixation (eq. 3) may not hold in small finite populations. Third, assuming a separate substitution process for each site is equivalent to assuming no linkage between sites (free recombination). In practice, however, there is limited recombination, at least within exons, and this could induce deviations from the mutation–selection approximation, due to Hill–Robertson effects.

The finite population was now modeled explicitly, using a Wright–Fisher simulator, tracking the frequency of each allele at the gene level and at each generation along the phylogeny. No recombination was implemented within genes. These more complex simulation settings account for small population size effects, for hitchhiking of weakly deleterious mutations during selective sweep and for background selection due to linkage disequilibrium. In addition, the effective population size $N_{e}$ and the mutation rate were allowed to fluctuate continuously along the branches of the tree (changing by a small amount after each generation of the underlying Wright–Fisher process). Finally, short-term fluctuations of $N_{e}$, of the order of 20% per generation, were accounted for by adding a random noise to the Brownian process describing the long-term evolution of $N_{e}$. In spite of these deviations between the simulation and the inference models, branch lengths and branch-specific effective population sizes could again be robustly recovered by the inference framework (slope of 0.868, $r^{2}=0.919$, fig. 2*B* and *E*).

These results are encouraging. However, they still rely on the assumption of a site-independent fitness landscape, which is equivalent to assuming no epistasis. Yet this assumption is almost certainly violated in practice (Pollock and Goldstein 2014; Shah et al. 2015). Accordingly, we implemented a more complex, site-dependent fitness landscape accounting for the selective interactions between sites induced by the 3-dimensional structure of protein. In this model, the conformational stability of the protein determines its probability of being in the folded state, which is in turn taken as a proxy for fitness (Williams et al. 2006; Goldstein 2011; Pollock et al. 2012). Under this evolutionary model, and at any given time, the fitness landscape at a particular codon site is dependent on the amino acids that are currently present at those sites that are in the vicinity of the focal site in 3D space (see supplementary, Supplementary Material online). When applied to data simulated using this model, our inference framework could accurately recover the simulated branch lengths (fig. 2*D*). On the other hand, the distribution of $N_{e}$ across the tree could not be accurately recovered (slope of 0.0196, $r^{2}=0.0122$, fig. 2*F*). In fact, no meaningful variation in $N_{e}$ is detected, and the little variation in $N_{e}$ that is inferred shows no correlation with the true branch-specific mean $N_{e}$ values. This effect can be explained by the predicted independence of $d_{N}/d_{S}$, and more generally of the scaled selection coefficients associated with nonsynonymous mutations, to changes in $N_{e}$ in this specific model of protein stability, as shown theoretically by Goldstein (2013).

As an alternative model of epistasis between sites, a Fisher geometric model was also considered for the simulations (see supplementary, Supplementary Material online). The results under this model are intermediate between simulations without epistasis and simulations under the biophysically inspired model considered above. More specifically, under data simulated using Fisher’s geometric model, the true and estimated branch-specific $N_{e}$ are strongly correlated with each other ($r^{2}=0.73$). On the other hand, the slope of the correlation is substantially <1 (0.571). In other words, the trends in $N_{e}$ across the tree are correctly recovered, but the range of the variation in effective population size over the tree is substantially underestimated. As for the branch lengths, they are again correctly estimated. In summary, our simulation experiments show that our inference framework is reliable in the absence of model misspecification and is robust to violations concerning short- versus long-term variation in $N_{e}$ or to the presence of empirically reasonable levels of Hill–Robertson interference. On the other hand, and very importantly, epistasis, which is ignored by the inference model, appears to lead to a general underestimation of the true variation in $N_{e}$, to an extent that depends on the exact epistatic model but can go as far as completely obliterating any signal about the true variation in $N_{e}$ across the tree in the most extreme situations.

### Empirical Experiments

We next applied our inference framework to a series of four empirical data sets spanning different taxonomic groups within metazoans. As a first empirical case, we considered a data set of 77 placental mammals, for which complete genome sequences and information about LHTs is available. Placental mammals offer an interesting example, for which effective population size is likely to show substantial variation across lineages. This variation in $N_{e}$ is expected to covary with LHTs, such that large-bodied species are expected to have smaller effective population sizes, compared with small-bodied species.

For computational reasons, we restricted our analyses to a random set 18 of orthologous genes, which are then concatenated into a single MSA for analysis. Of note, the mutation–selection model considered here assumes that the fitness profiles do not change with time. In contrast, some genes might experience fluctuating fitness landscapes through time. Such fluctuations are in fact one main cause of ongoing adaptation (Mustonen and Lässig 2009; Rodrigue and Lartillot 2017). For that reason, genes for which positive selection was detected using a site codon model were excluded from the analysis. To assess the reproducibility of our inference and check that the signal about variation in $N_{e}$ is not driven by particular genes, we analyzed in total four different concatenated MSA each containing 18 randomly sampled genes. The different concatenated MSA showed similar trends in the change of *μ* and $N_{e}$ between pairs of replicates (see supplementary, Supplementary Material online).

**Fig. 3.**
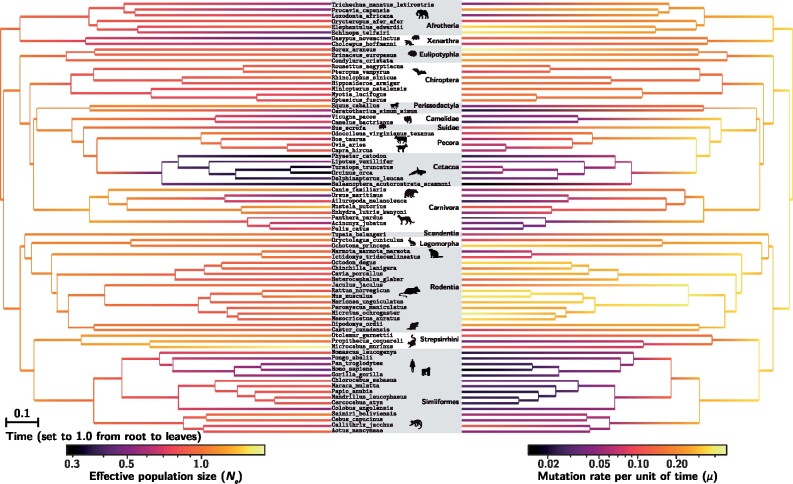
Inferred phylogenetic history of $N_{e}$ (left) and *μ* (right) across placental mammals (posterior mean estimate), based on an analysis of a concatenation of 18 genes randomly chosen among single-copy orthologs putatively under an exclusively purifying selection regime. $N_{e}$ estimates are relative to the value of $N_{e}$ at the root. The scale for *μ* is per nucleotide site and per total tree depth (i.e., total time from the most recent common ancestor to the present). If we assume the root to be 105 My old (Meredith et al. 2011; Kumar et al. 2017), the rescaled mutation rate per site per year in extant species is between $1.1\times 10^{-10}$ and $7.8\times 10^{-9}$. Icons are adapted from http://phylopic.org.

The reconstructed long-term changes in effective population size ($N_{e}$) is displayed in figure 3. We visually observe a global trend of increasing $N_{e}$ throughout the tree around 90 and 60 My. We also observe $N_{e}$ to be lower in some clades, such as Cetacea and Camelidae, whereas being higher in other clades, such as Rodentia and Pecora. In some cases, a decrease in $N_{e}$ can be observed along an isolated branch of the tree, for example on the branches leading to the Alpaca (*Vicugna pacos*) or the cheetah (*Acinonyx jubatus*).

The estimated covariance matrix (table 1) gives a global synthetic picture of the patterns of covariation between the mutation rate per unit of time *μ*, the effective population size $N_{e}$ and the three LHTs. First, *μ* covaries negatively with body mass, age at sexual maturity and longevity (table 1). These correlations, which were previously reported (Lartillot and Delsuc 2012; Nabholz et al. 2013) probably reflect generation time effects (Lanfear et al. 2010; Gao et al. 2016). Similarly, and more interestingly in the present context, $N_{e}$ covaries negatively with LHTs (table 1). This is consistent with the expectation that small-sized and short-lived species tend to be characterized by larger effective population sizes (Romiguier et al. 2014). Of note, these results mirror previous findings, based on classical codon models, showing that $d_{N}/d_{S}$ tends to be positively correlated with LHTs (Lartillot and Delsuc 2012; Nabholz et al. 2013; Figuet et al. 2017). This positive correlation between $d_{N}/d_{S}$ and LHTs was also recovered on the present data set, using a classical $d_{N}/d_{S}$ based codon model (supplementary materials, Supplementary Material online). Interestingly, the correlation between $d_{N}/d_{S}$ and LHTs is weaker than the correlation between our inferred $N_{e}$ and LHTs, as expected if the variation in $d_{N}/d_{S}$ indirectly (and imperfectly) reflects the underlying variation in $N_{e}$. Finally, $N_{e}$ and *μ* are positively correlated in their variation (ρ=0.44), which might simply reflect the fact that both covary negatively with LHTs. The partial-correlation coefficients (see supplementary, Supplementary Material online) between $N_{e}$ and LHTs are not significantly different from 0. However, this might simply be due to the very strong correlation between the three LHTs considered here, such that controlling for any one of them removes most of the signal contributed by the empirically available variation between species.

**Table 1.** Correlation Coefficients between Effective Population Size ($N_{e}$), Mutation Rate per Site per Unit of Time (*μ*), and Life-History Traits (maximum longevity, adult weight, and female maturity).

Correlation (ρ)	$N_{e}$	μ	Maximum Longevity	Adult Weight	Female Maturity
$N_{e}$	—	0.439*	–0.525*	–0.544*	–0.47*
μ	—	—	–0.832*	–0.835*	–0.833*
Maximum longevity	—	—	—	0.827*	0.845*
Adult weight	—	—	—	—	0.809*
Female maturity	—	—	—	—	—

* Note.—Asterisks indicate strength of support of the posterior probability to be different than 0 (pp) as pp>0.975.

Thus, altogether, the inferred trends in $N_{e}$ across species appear to be as expected, based on considerations about life-history evolution. On the other hand, the total range of the inferred variation in $N_{e}$ across the entire extant taxa is surprisingly narrow, with one order of magnitude (9.2) at most between high and low $N_{e}$ (see supplementary, Supplementary Material online). This almost certainly represents an underestimate of the true range of variation across placental mammals.

As another case study, we analyzed a group of isopod species that have made multiple independent transitions to subterranean environments. The transition from a terrestrial to a subterranean lifestyle is typically associated with a global life-history and ecological syndrome characterized by a loss of vision, longer generation times and, most interestingly, smaller population sizes, due to a lower carrying capacity of the subterranean environment (Capderrey et al. 2013). Protein coding DNA sequence alignments and qualitative LHTs, such as habitat (surface or underground), pigmentation (depigmented, partially depigmented or pigmented), and ocular structure (anophthalmia, microphthalmia, or ocular) are available for these species (Eme et al. 2013; Saclier et al. 2018). The assumption of a Brownian autocorrelated process for describing the changes in $N_{e}$ along the tree may not be so well adapted to the present case, since the changes in $N_{e}$ associated with the transition to a subterranean environment are likely to correspond to relatively sudden shifts, rather than continuous variation, and the ecological correlate (subterranean vs. terrestrial) is not a quantitative trait. However, the data set considered here contains independent transitions to a subterranean lifestyle, thus offering an opportunity to test for a potential correlation between inferred $N_{e}$ variation and terrestrial versus subterranean lifestyles over the terminal branches.

**Fig. 4.**
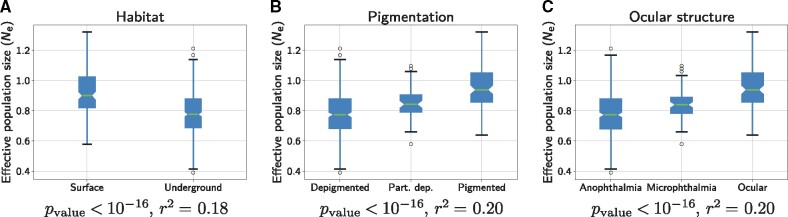
$N_{e}$ estimation for extant isopods species sorted according to their habitat (*A*), pigmentation (*B*), and ocular structure (*C*). Estimated $N_{e}$ are pooled across six different multiple sequence alignments (MSA) each containing 12 randomly sampled genes in isopods species. All three qualitative traits statistically correlate with changes in $N_{e}$ in the terminal branches.

To assess the reproducibility of our inference, we analyzed in total six different concatenated MSA each containing 12 randomly sampled genes. The six different concatenated MSA showed similar trends in the change of *μ* and $N_{e}$ between pairs of replicates (see supplementary, Supplementary Material online). A statistical analysis performed on the pooled estimation of $N_{e}$ across the six different concatenated MSA exhibits a statistically significant reduction in $N_{e}$ for underground or depigmented species, or for species with visual impairment (see fig. 4). Of note, the species that did not undergo a transition to subterranean environments feature a relative $N_{e}$ close to 1, meaning that $N_{e}$ has not changed much along the lineages (since the root of the tree). Again, the total range of the inferred variation in $N_{e}$ across the entire extant taxa is surprisingly narrow, with ratio of 3.3 at most between high and low $N_{e}$ (see supplementary, Supplementary Material online).

Next, our empirical framework was also applied on a set of genes sampled across primates, taken from Perelman et al. (2011) and reanalyzed in Brevet and Lartillot (2021). In addition to LHTs (mass, female maturity, generation time and longevity), information about nuclear synonymous diversity (***π***_*S*_) and nonsynonymous over synonymous diversity ($\pi_{N}/\pi_{S}$), are available for 10 species across the data set and are expected to correlate with $N_{e}$ according to population genetics (Eyre-walker and Keightley 2007; Galtier 2016). However, the correlation coefficient between our inferred $N_{e}$ and ***π***_*S*_ or $\pi_{N}/\pi_{S}$ and LHTs are not statistically significant, nor with LHTs (see supplementary, Supplementary Material online). Again, the total range of the inferred variation in $N_{e}$ across the entire tree is narrow, with ratio of 6.4 at most between high and low $N_{e}$. These results contrast with the finding of Brevet and Lartillot (2021) on the same data set based on $d_{N}/d_{S}$-based codon models, where the estimated $N_{e}$ was found to span several orders of magnitude, and correlated positively with ***π***_*S*_.

## Discussion

Mechanistic phylogenetic codon models express the substitution rates between codons as a function of the mutation rates at the nucleotide level, selection over amino-acid sequences and effective population size. Thus far, the development of mutation–selection models of the Halpern and Bruno (1998) family (Rodrigue et al. 2010; Tamuri et al. 2012) has mostly focused on the question of fully accounting for the fine-scale modulations of selection between amino-acids and across sites (Rodrigue et al. 2010; Tamuri et al. 2012). However, the issue of the variation in the global population-genetic regime between species has received much less attention. In particular, effective population size ($N_{e}$) is expected to vary substantially over the species of a given clade, yet current mutation–selection models all invariably assume $N_{e}$ to be constant across the phylogeny.

Here, we have introduced an extension of the mutation–selection model that accounts for this variation. When applied to an alignment of protein coding sequences, this mechanistic model returns an estimate of the modulations of amino-acid preferences across sites. Simultaneously, it reconstructs the joint evolution of LHTs and molecular and population-genetic parameters (mutation rate *μ* and effective population size $N_{e}$) along the phylogeny, whereas estimating the correlation matrix between these variables, intrinsically accounting for phylogenetic inertia.

### Reliability of the Inference of the Phylogenetic History of $N_{e}$

The reconstructions obtained on several empirical data sets, in particular in mammals and in isopods, suggest that the method is able to correctly infer the directional trends of the changes in $N_{e}$ across species. In particular, in mammals, the inferred variation in $N_{e}$ correlates negatively with body size and, more generally, with LHTs, as expected under the reasonable assumption that large-bodied mammals would tend to have smaller effective population sizes (Popadin et al. 2007; Lartillot and Delsuc 2012; Nabholz et al. 2013; Figuet et al. 2017). Similarly, in isopods, smaller effective population sizes are inferred in subterranean species, again, as expected (Capderrey et al. 2013).

However, although the changes in $N_{e}$ are in the expected direction (negative correlation with body size, weight, and maturity) (Romiguier et al. 2014), the magnitude of the changes inferred across the phylogeny is surprisingly narrow (at most a factor 9.2 in mammals). This range does not match independent empirical estimates of the variation in mammals, where synonymous diversity varies by a factor at least 10 between species (Galtier 2016). In animals, the synonymous diversity roughly spans two orders of magnitude, whereas $N_{e}$ varies considerably more across species, by a factor of 10^3^ (Galtier and Rousselle 2020). For instance, effective population sizes estimated based on population genomic data are of the order of 10,000 in humans (Li and Durbin 2011), and 100,000 in mice (Geraldes et al. 2008). Thus, clearly, our approach underestimates the true variation. Different mechanisms not accounted for by the model could explain this result.

First, genetic hitchhiking, Hill–Robertson interference, and short-term fluctuations of $N_{e}$ could generate this effect. However, inference conducted on alignments simulated under a Wright–Fisher model accounting for linkage and for short-term variation in $N_{e}$ suggests that empirically reasonable levels of Hill–Robertson interferences are not strong enough to explain this observation, at least in the regimes explored. Second, *μ* and $N_{e}$ could also be fluctuating along the genome (Ellegren et al. 2003; Gossmann et al. 2011; Eyre-Walker and Eyre-Walker 2014). This assumption needs to be tested, though we expect that relaxing this assumption would not change drastically the magnitude of inferred $N_{e}$ since some of this fluctuation should be absorbed by the inferred site-specific fitness profiles. Third, the DNA sequences could also be misaligned at some sites. However, we observe the same magnitude of inferred $N_{e}$ for different sets of genes indicating this might not be the primary reason. Fourth, the genes selected in our alignments could be under adaptive evolution, or their function could have changed. However, at least in mammals, the impact of this potential problem was minimized by the use of genes for which no positive selection was detected using standard phylogenetic codon site models.

Finally, one key assumption of the mutation–selection model that is likely to be violated in practice is the assumption of site-independence. In reality, epistasis might be prevalent in protein coding sequence evolution (Pollock and Goldstein 2014; Shah et al. 2015). Our simulations under an epistatic landscape point to epistasis being a major factor to be investigated. Indeed, $N_{e}$ could not be appropriately estimated under these simulation settings, although the outcome more specifically depends on the exact model for the fitness landscape. An extreme case is obtained using a biophysically inspired model, assuming purifying selection for conformational stability. This model was previously explored using simulations and theoretical developments Goldstein (2013), and it was shown that, under this model, $d_{N}/d_{S}$ and more generally the substitution process is virtually insensitive to $N_{e}$. This is confirmed by our experiments, showing that the mutation–selection approach explored here cannot infer the true variation in $N_{e}$ under this model.

A less extreme outcome is obtained under an alternative model also implementing epistatic interactions between sites via Fisher’s geometric model (Tenaillon 2014; Blanquart and Bataillon 2016). Interestingly, under this model, our inference framework is able to infer the correct trends of $N_{e}$, although with a substantially underestimated range of inferred variation, thus mirroring the results obtained on placental mammals. Of note, these results do not necessarily imply that models based on biophysics are empirically less relevant than Fisher’s geometric model. Instead, they might just betray that the response of the substitution process to changes in $N_{e}$ may be sensitive to the exact quantitative details of the underlying fitness landscape. More work is probably needed here to characterize these exact conditions. Nevertheless, our simulation experiments suggest a global pattern: Epistatic interactions induce a buffering of the response of the substitution process to changes in $N_{e}$. The meaningful correlation patterns observed with LHTs in the case of placental mammals suggest that this buffering is not complete. Nevertheless, ignoring epistatic interactions at the inference level appears to result in a substantial underestimation of the range over which $N_{e}$ varies across species.

Interestingly, the magnitude of the inferred range of $N_{e}$ variation is similar for the placental and the primate data sets (with a 9-fold and 6-fold variation in mammals and primates, respectively), whereas one would have expected a much larger range of variation over the broader phylogenetic scale of placental mammals, compared with primates. An explanation could be that the effects of epistasis are more apparent at longer time-scales. Indeed, the total number of substitutions from root to leaves is greater, and as a result, the local environment, and therefore the fitness landscape at the level of each site, has been less stable across the phylogeny.

Although modeling epistasis in an inference framework is a complex biological, mathematical and computational problem, our work points to a potential signal of epistasis that could be retrieved in a phylogenetic context. More specifically, since the slope of the response of the substitution process to changes in $N_{e}$ appears to be informative about the epistatic regime, then, conversely, by relying on independent estimates of $N_{e}$ (e.g., using polymorphism), this effect could be used to leverage a quantitative estimate of the statistical distribution of epistatic effects.

Other methods have recently been developed to reconstruct phylogenetic changes in $N_{e}$. For example, a method recently developed uses polymorphism and generation time for some present-day species to reconstruct $N_{e}$ along the phylogeny, based on a classical ($d_{N}/d_{S}$-based) codon model (Brevet and Lartillot 2021). This method implicitly relies on a nearly neutral model, assuming a fixed and gamma-shaped distribution of fitness effects across nonsynonymous mutations. The approach is calibrated using fossils, and as a result, returns estimates of the absolute value of $N_{e}$ and of its phylogenetic variation. Here, in contrast, our method requires neither generation times nor polymorphism data, and the fitness effects are not constrained to a specific distribution. On the other hand, the inferred effective population sizes are only relative.

### Potential Applications and Future Developments

Apart from reconstructing the phylogenetic history of $N_{e}$ and investigating its causes and covariates, another potentially interesting application of our approach is in detecting adaptation. In this direction, mutation–selection models represent a useful null nearly neutral model, explicitly modeling the background of purifying selection acting over protein coding genes. Adaptation can then be detected by measuring the deviation from this null model (Rodrigue and Lartillot 2017; Bloom 2017).

However, by assuming a constant $N_{e}$ along a phylogeny, the statistical power of this approach to detect sites under adaptive evolution may not be optimal. In particular, the site-specific fitness profiles inferred by the model are averaged along the phylogeny and are seemingly more diffuse than those estimated profiles under our present framework (see supplementary materials, Supplementary Material online). Thus, our method should provide a better null model of purifying selection against which to test for the presence of adaptive evolution.

This approach can be further extended in other directions. First, the mutation rate (*μ*) is considered site-invariant, an assumption which could be relaxed by introducing site-specific mutation rate to account for variation in mutation rate along the sequence.

Second, currently, our model also assumes no selection on codon usage. In the case of primates or placental mammals, this assumption is probably reasonable (Yang and Nielsen 2008), although it is more questionable for other groups, in particular Drosophila (Duret and Mouchiroud 1999; Plotkin and Kudla 2011). In principle, this assumption can be relaxed by implementing selective codon preferences that are shared across all sites (Nielsen et al. 2007). Such an implementation would provide the advantage of estimating codon usage biases, whereas simultaneously accounting for its confounding effect when estimating selection on amino-acids and interspecific variation in $N_{e}$.

Third, providing a computationally more efficient implementation of the model would be important for broader application. Currently, running the program on an MSA of 18 mammals genes (77 extant species, and on the order of 15,000 nucleotide sites) for 4,000 iterations of the chain (1,000 are left as burn-in) takes approximately 2–4 weeks of computations, which is quite long although still accessible for reasonably small data sets. Increasing the computational efficiency could be achieved by several means: First, parallelizing the program could be achieved by dispatching genes over multiple cores. Second, a large fraction of the computing time is spent in updating the fitness profiles, and thus, fixing them to empirical values or using pre-estimated profiles under a constant $N_{e}$ would lead to a substantial acceleration.

Finally, estimating $N_{e}$ in a mutation–selection phylogenetic model relies on the relation between $N_{e}$ and the relative strength of drift, in a context where, ultimately, the signal about the intensity of drift comes from the rate of nonsynonymous substitutions relative to that of synonymous substitutions. However, this purely phylogenetic approach does not leverage a second aspect of $N_{e}$ at the population level, namely, the fact that $N_{e}$ also determines the levels of neutral genetic diversity that can be maintained ($\pi =4N_{e}u$, where *u* is the mutation rate per generation). Hence, neutral diversity yields an independent empirical estimate of $N_{e}$. In principle, our mechanistic model could be extended so as to incorporate polymorphism data within species at the tips of the phylogeny. A similar method has been previously pioneered in the case of three species and using a distribution of fitness effect (Wilson et al. 2011). More generally, the nearly neutral theory of evolution defines a long-term $N_{e}$, which might be different from the short-term definition of $N_{e}$ (Platt et al. 2018). Thus we could ask if empirical independent estimations of $N_{e}$ from within species (based on genetic diversity) and between species (based on the substitution process) are congruent, and if not, what are the mechanisms responsible for this discrepancy.

Notwithstanding theoretical considerations on the nearly neutral theory of evolution, empirical clues about the long-term trends in the modulations of the intensity of genetic drift opens up a large diversity of ecological and evolutionary questions. Spatial and temporal changes of genetic drift along ecological niches and events can now be investigated, so as to disentangle the underlying evolutionary and ecological pressures.

## Materials and Methods

In the model presented here, $N_{e}$ and *μ* and quantitative traits are allowed to vary between species (among branches) as a multivariate geometric Brownian process, but assumed constant along the DNA sequence. Conversely, amino-acid fitness profiles are assumed to vary across sites, but are considered constant along the tree. The model makes several assumptions about the evolutionary process generating the observed alignment. First, the species tree topology is supposed to be known, and each gene should match the species tree, meaning genes are strict orthologs (no paralogs and no horizontal transfers). Second, there is no epistasis (interaction between sites), such that any position of the sequence has its own independent evolutionary process and a substitution at one position does not affect the substitution process at other positions. Third, from a population genetics perspective, we assumed sites of the protein to be unlinked, or equivalently the mutation rate is low enough such that there is neither Hill–Robertson interference nor genetic hitchhiking. Fourth, polymorphism is ignored in extant species.

**Fig. 5.**
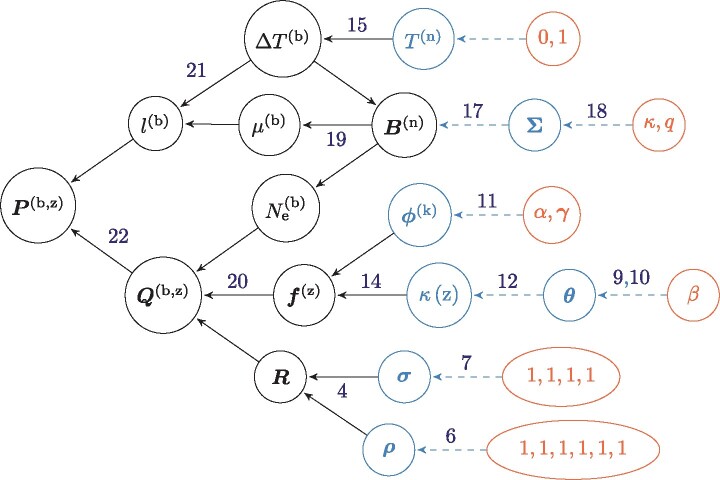
Directed acyclic graph (DAG) of dependencies between variables. Nodes of the directed acyclic graph are the variables, and edges are the functions. Hyper-parameters are depicted in red circles, random variables in blue circles, and transformed variables in black. Blue dashed line denotes a drawing from a random distribution, and black solid lines denote a function. All the nodes pointing toward a given node (upstream) are its dependencies which determine its distribution. The other way around, following the arrows in the DAG (downstream), simple prior distributions are combined together to form more complex joint prior distribution which ultimately defines the prior distribution of the model.

The parameterization of the models is described as a Bayesian hierarchical model, including the prior distributions and the parameters of the model. This hierarchical model is formally represented as directed acyclic graph, depicted in figure 5.

### Nucleotide Mutation Rates

The generalized time-reversible nucleotide mutation rate matrix ***R*** is a function of the nucleotide frequencies σ and the symmetric exchangeability rates ρ (Tavar**é**^1986^). $\sigma =(\sigma_{A},\sigma_{C},\sigma_{G},\sigma_{T})$ is the equilibrium base frequency vector, giving the frequency at which each base occurs at each site. $\rho =\left(\rho_{AC},\rho_{AG},\rho_{AT},\rho_{CG},\rho_{CT},\rho_{GT}\right)$ is the vector of exchangeabilities between nucleotides. Altogether, the rate matrix is:

(4) $$R=\begin{array}{c} A\\ C\\ G\\ T \end{array}\left(\begin{array}{cccc} - & \rho_{AC}\sigma_{C} & \rho_{AG}\sigma_{G} & \rho_{AT}\sigma_{T}\\ \rho_{AC}\sigma_{A} & - & \rho_{CG}\sigma_{G} & \rho_{CT}\sigma_{T}\\ \rho_{AG}\sigma_{A} & \rho_{CG}\sigma_{C} & - & \rho_{GT}\sigma_{T}\\ \rho_{AT}\sigma_{A} & \rho_{CT}\sigma_{C} & \rho_{GT}\sigma_{G} & - \end{array}\right).$$

By definition, the sum of the entries in each row of the nucleotide rate matrix ***R*** is equal to 0, giving the diagonal entries:

(5) $$R_{a,a}=-\sum_{b\neq a,b\in \{A,C,G,T\}}R_{a,b}.$$

The prior on the exchangeabilities ρ is a uniform Dirichlet distribution of dimension 6:

(6) ρ∼Dir(1,1,1,1,1,1).

The prior on the equilibrium base frequencies σ is a uniform Dirichlet distribution of dimension 4:

(7) σ∼Dir(1,1,1,1).

The general time-reversible nucleotide matrix is normalized such that the total flow equals to 1:

(8) $$\sum_{a\in \{A,C,G,T\}}-\sigma_{a}R_{a,a}=1.$$

### Site-Dependent Selection

Site-specific amino-acid fitness profiles are assumed i.i.d. from a mixture model, itself endowed with a truncated Dirichlet process prior. Specifically, the mixture has K components (K=50 by default). The prior on component weights (θ) is modeled using a stick-breaking process, truncated at K and of parameter **_*β*_**:

(9) $$\begin{array}{c} \theta \sim \text{StickBreaking}\left(K,\beta \right)\\ \Leftrightarrow \theta_{k}=\psi_{k}\cdot \prod_{a=1}^{k-1}\left(1-\psi_{a}\right),\;k\in \{1,\ldots ,K\}, \end{array}$$

where $\psi_{k}$ are i.i.d. from a beta distributio*n*

(10) $$\psi_{k}∼\text{Beta}\left(1,\beta \right),\;k\in \{1,\ldots ,K\}.$$

Of note, the weights decrease geometrically in expectation, at rate ***β***, such that lower values of ***β*** induce more heterogeneous distributions of weights.

Each component of the mixture defines a 20-dimensional fitness profile $\phi^{\left(k\right)}$ (summing to 1), for k∈{1,…,K}. These fitness profiles are i.i.d. from a Dirichlet of center γ and concentration ***α***:

(11) $$\phi^{\left(k\right)}∼\text{Dir}\left(\gamma ,\;\alpha \right),\;k\in \{1,\ldots ,K\}.$$

Site allocations to the mixture components κ(z)∈{1,…,K}, for z∈{1,…,Z} running over the *Z* sites of the alignment, are i.i.d. multinomial of parameter θ:

(12) m∼Multinomial(θ),

(13) $$\;\text{where}\;m_{k}=\sum_{z\in \{1,\;\ldots ,\;Z\}}1_{\kappa \left(z\right)=k}\;.$$

For a given parameter configuration for the mixture, the Malthusian fitness selection coefficients $f^{\left(z\right)}$ at site *z*, is obtained by taking the logarithm of the fitness profile assigned to this site:

(14) $$f^{\left(z\right)}=\ln\left(\phi^{(\kappa \left(z)\right)}\right),\;z\in \{1,\ldots ,Z\}.$$

### Dated Tree

The topology of the rooted phylogenetic tree is supposed to be known and is not estimated by the model. The model estimates the dates at which branches split, thus the dated tree requires P−2 internal node ages that are free parameters, where *P* is the number of extant taxa (leaves of the tree). By definition, leaf ages are all set to 0. The root age is set arbitrarily to 1, but if fossils data are also available the dated tree can be rescaled into absolute time using cross-multiplication. A uniform prior is assumed over internal node ages $T^{\left(n\right)},\;n\in \{P+1,\ldots ,2P-2\}$.

The duration $\Delta T^{\left(b\right)}$ represented by a given branch *b*, for b∈{1,…,2P−2} is defined as the difference in ages between the oldest node at the tip of the branch $T^{\left(b^{↑}\right)}$, and the youngest node $T^{\left(b^{↓}\right)}$:

(15) $$\Delta T^{\left(b\right)}=T^{\left(b^{↑}\right)}-T^{\left(b^{↓}\right)}.$$

### Branch Dependent Traits

The effective population size $N_{e}$ and mutation rate per unit of time *μ* are assumed to evolve along the phylogeny, and to be correlated. If quantitative LHTs are also available for some nodes of the tree (leaves and/or internal nodes), they are also assumed to evolve along the phylogeny and to be correlated between them, and with $N_{e}$ and *μ*. The total number of traits is noted *L*, when counting $N_{e}$, *μ* and all user-defined LHT (denoted ***X***). Their variation through time is modeled by an *L*-dimensional geometric Brownian process ***B***. By convention, the first component of the log-Brownian corresponds to $N_{e}$, and the second component to *μ*. Thus:

(16) $$\left\{ \begin{array}{c} B_{1}(t)=\ln N_{e}(t),\\ B_{2}(t)=\ln\mu (t),\\ B_{k+2}(t)=\ln X_{k}(t),k\in \{1,\ldots ,L\}. \end{array}\right.$$

The effective population size at the root is set to 1 for identifiability of the fitness profiles.

Along a branch b∈{1,…,2P−2} of the tree, a geometric Brownian process starts at the oldest node at the tip of the branch ($b^{↑}$), and ends at the youngest node ($b^{↓}$). The rate of change of the geometric Brownian process per unit of time is constant and determined by the positive semidefinite and symmetric covariance matrix Σ. Thus the distribution at node $b^{↓}$ of $B^{\left(b^{↓}\right)}$ is multivariate Gaussian, with mean equals to the Brownian process sampled at the oldest node $B^{\left(b^{↑}\right)}$, and variance $\Delta T^{\left(b\right)}\Sigma$:

(17) $$B^{\left(b^{↓}\right)}\sim N\left(B^{\left(b^{↑}\right)},\Delta T^{\left(b\right)}\Sigma \right),\;b\in \{1,\ldots ,2P-2\}.$$

The Brownian process at the root of the tree is uniformly distributed, except for the first component fixed to 0 for identifiability (see above). The prior on the covariance matrix is an inverse Wishart distribution, parameterized by ***κ***= 1 and with q=L+1 degrees of freedom:

(18) $$\Sigma ∼{\text{Wishart}}^{-1}(\kappa I,q).$$

We are interested in approximating the expected substitution rates between codons over the branch. Ideally, under the Brownian process just described, the rates of substitution between codons are continuously changing through time. Also, even conditional on the value of $N_{e}$ at both ends, the Brownian path along the branch entails a random component, leading to complicated integral expressions for substitution rates (Horvilleur and Lartillot 2014). Here, a branchwise approximation is used (Lartillot and Poujol 2011), which consists of first deriving an approximation for the mean $N_{e}$ along the branch, conditional on the values of $N_{e}$ at both ends, and then using this mean branchwise $N_{e}$ to define the codon substitution rates.

In the case of geometric Brownian process, the most likely path (or geodesic) from $B^{\left(b^{↑}\right)}$ to $B^{\left(b^{↓}\right)}$ is the straight line, and therefore, it would make sense to take the mean value of $e^{B^{\left(n\right)}}$ along this geodesic. We then have $N_{e}^{\left(b\right)}$ and $\mu^{\left(b\right)}$ for each branch b∈{1,…,2P−2} of the tree:

(19) $$\left\{ \begin{array}{c} N_{e}^{\left(b\right)}=\frac{e^{B_{1}^{\left(b^{↓}\right)}}-e^{B_{1}^{\left(b^{↑}\right)}}}{B_{1}^{\left(b^{↓}\right)}-B_{1}^{\left(b^{↑}\right)}},\\ \mu^{\left(b\right)}=\frac{e^{B_{2}^{\left(b^{↓}\right)}}-e^{B_{2}^{\left(b^{↑}\right)}}}{B_{2}^{\left(b^{↓}\right)}-B_{2}^{\left(b^{↑}\right)}}. \end{array}\right.$$

### Codon Substitution Rates

The mutation rate between codons *i* and *j*, denoted $\mu_{i,j}$ depends on the underlying nucleotide change between the codons. First, if codons *i* and *j* are not nearest-neighbors, $\mu_{i,j}$ is equal to 0. Second, if codons *i* and *j* are only one mutation away, M(i,j) denotes the nucleotide change (e.g., M(AAT,AAG)=TG), and $\mu_{i,j}$ is given by the underlying nucleotide relative rate ($R_{M(i,j)}$) scaled by the mutation rate per time (*μ*). Technically, the 4-dimensional nucleotide relative rate matrix (***R***) is normalized such that we expect one substitution per unit of time, hence the scaling by *μ*.

For a given branch *b* and a given site *z*, the codon substitution rate (per unit of branch lenght) matrix $Q^{\left(b,z\right)}$ is given by:

(20) $$\left\{ \begin{array}{c} Q_{i,j}^{\left(b,z\right)}=0\;\text{if codons }i\;\text{and }j\;\text{are not nearest-neighbors},\\ Q_{i,j}^{\left(b,z\right)}=R_{M(i,j)}\;\text{if codons }i\;\text{and }j\;\text{are synonymous},\\ Q_{i,j}^{\left(b,z\right)}=R_{M(i,j)}\frac{4N_{e}^{\left(b\right)}\left(f_{A(j)}^{\left(z\right)}-f_{A(i)}^{\left(z\right)}\right)}{1-e^{4N_{e}^{\left(b\right)}\left(f_{A(i)}^{\left(z\right)}-f_{A(j)}^{\left(z\right)}\right)}}\text{if }i\;\text{and }j\;\text{are nonsynonymous,}\\ Q_{i,i}^{\left(b,z\right)}=-\sum_{j\neq i,j=1}^{61}Q_{i,j}^{\left(b,z\right)}. \end{array}\right.$$

We see from this equation that, *f* and $N_{e}$ are confounded, such that increasing the effective population size whereas decreasing the fitnesses by the same factor leads to the same substitution rate.

The branch lengths $l^{\left(b\right)}$ are defined as the expected number of neutral substitutions per DNA site along a branch:

(21) $$l^{\left(b\right)}=\mu^{\left(b\right)}\Delta T^{\left(b\right)}.$$

Together, the probability of transition between codons for a given branch *b* and site *z* is:

(22) $$P^{\left(b,z\right)}=e^{l^{\left(b\right)}Q^{\left(b,z\right)}},$$

which are the matrices necessary to compute the likelihood of the data (*D*) given the parameters of the model using the pruning algorithm.

### Bayesian Implementation

Bayesian inference was conducted using MCMC. Most phylogenetic MCMC samplers target the distribution over the model parameters given the sequence alignment, which means that they have to repeatedly invoke the pruning algorithm to recalculate the likelihood which is most often the limiting step of the MCMC. An alternative, which is used here, is to do the MCMC conditionally on the detailed substitution history H, thus doing the MCMC over the augmented configuration (H, *D*), under the target distribution obtained by combining the mapping-based likelihood with the prior over model parameters.

The key idea that makes this strategy efficient is that the mapping-based likelihood depends on compact summary statistics of H, leading to very fast evaluation of the likelihood. On the other hand, this requires to implement more complex MCMC procedures that have to alternate between:

1. sampling H conditionally on the data and the current parameter configuration.
2. resampling the parameters conditionally on H.

To implement the mapping-based MCMC sampling strategy, we first sample the detailed substitution history H for all sites along the tree. Several methods exist for doing this (Nielsen 2002; Rodrigue et al. 2008), which are used here in combination (first trying the accept-reject method of Nielsen, then switching to the uniformization approach of Rodrigue et al. if the first round has failed).

Then, we write down the probability of H given the parameters, and finally, we collect all factors that depend on some parameter of interest and make some simplifications. This ultimately leads to relatively compact sufficient statistics (see supplementary, Supplementary Material online) allowing for fast numerical evaluation of the likelihood (Irvahn and Minin 2014; Davydov et al. 2017). As an example, making an MCMC move on the $N_{e}$ at a given node of the tree is faster since only the mapping-based likelihood (using path sufficient statistics) at the neighboring branches of the node is necessary, instead of computing the likelihood for the entire tree.

MCMC are run for 4,000 points and the first 1,000 points are discarded as burn-in. Convergence is then assessed (see supplementary, Supplementary Material online) by comparing two independent chains, checking that both site-specific fitness and branch $N_{e}$ have the same posterior mean.

### Correlation between Traits

The correlation between trait *a* and trait b∈{1,…,L} can be obtained from the covariance matrix **Σ**:

(23) $$\rho_{a,b}=\frac{\Sigma_{a,b}}{\sqrt{\Sigma_{a,a}\Sigma_{b,b}}}.$$

This correlation coefficient is then averaged over the posterior distribution, and statistical support is assessed based on the posterior probability of having a positive (or negative) value for the coefficient.

### Simulations

To test the robustness of the model, four parameterized simulators were developed: SimuDiv, SimuPoly, SimuFold, & SimuGeo. All four simulators use a geometric Brownian multivariate process to model the changes in the mutation rate per generation, the generation time and $N_{e}$ along the lineages. SimuDiv, SimuFold, & SimuGeo all simulate point substitutions along the phylogenetic tree. In our simulations, the tree is composed of 77 species (see supplementary, Supplementary Material online), the tree root is 150 million years old, the initial mutation rate is $10^{-8}$ per site per generation and the initial generation time is 10 years. The simulator starts from an initial sequence at equilibrium, composed of 15,000 codon sites. The change in fitness is computed for all possible mutations, hence computing all strictly positive substitution rates. At each point, the next substitution is chosen proportional to these rates using in Gillespie’s algorithm (Gillespie 1977). At each node, the process is split, and finally stopped at the leaves of the tree. SimuPoly simulates explicitly each generation along the phylogeny under a Wright–Fisher population, consisting of three steps: Mutation, selection and genetic drift of currently segregating alleles. Mutations are drawn based on a user-defined nucleotide matrix, where our simulations used a symmetric time-reversible mutation matrix. Drift is induced by the multinomial resampling of the currently segregating alleles. We assume that the DNA sequence is composed of exons, with no linkage between exons, and total linkage of sites within an exon. Moreover, in SimuPoly, the instant value of log-$N_{e}$ can also be modeled as a sum of a geometric Brownian process and an Ornstein–Uhlenbeck process. The geometric Brownian motion accounts for long-term fluctuations, whereas the Ornstein–Uhlenbeck introduces short-term fluctuations. In SimuDiv and SimuPoly, each codon site contributes independently to the fitness depending on the encoded amino acids, through site-specific amino-acid fitness profiles experimentally determined (Bloom 2017). In SimuFold, the fitness of a sequence is computed as the probability of the protein to be in the folded state. SimuFold is a C++ adaptation of a Java code previously published (Goldstein and Pollock 2016, 2017), where we also allow for changes in $N_{e}$ and *μ* along a phylogenetic tree. Supplementary materials, Supplementary Material online describe the simulations in more details, with parameters and configurations used to produce alignments, as well as performance of the inference model against them.

### Empirical Data

For placental mammals, alignments were extracted from OrthoMam database (Ranwez et al. 2007; Scornavacca et al. 2019). LHTs for longevity, age at maturity and weight were obtained from AnAge database (De Magalhães and Costa 2009; Tacutu et al. 2012). We focused our analysis on 77 taxa for which information is available for at least one LHT. The list of conserved genes putatively not under positive selection is available in supplementary materials, Supplementary Material online.

## Supplementary Material

Supplementary data are available at *Molecular Biology and Evolution* online.

## Supplementary Material

msab160_Supplementary_Data

## Data Availability

The data underlying this article are available in Github, at https://github.com/ThibaultLatrille/MutationSelectionDrift (last accessed June 06, 2021), as well as scripts and instructions necessary to reproduce the simulated and empirical experiments. The Bayesian inference model, written in C++ in the component based (Lanore 2019) software BayesCode, is available at https://github.com/ThibaultLatrille/bayescode (last accessed June 06, 2021). The simulators written in C++ are available at https://github.com/ThibaultLatrille/SimuEvol (last accessed June 06, 2021). Supplementary materials describing additional analysis are available online.
