# Supplementary material for: Inferring Long-Term Effective Population Size with Mutation–Selection Models
Source: Mol Biol Evol. 2021 Jun 30;38(10):4573–87. doi: 10.1093/molbev/msab160 (PMC8476147; doi:10.1093/molbev/msab160)
Supplement: msab160_Supplementary_Data [file msab160_supplementary_data.zip › supp-mat.pdf]

Supplementary materials

Contents

1 Summary statistics **1**
1.1 Partial correlation coefficient . . . 1
1.2 Fitness profile entropy . . . 2

2 Simulations **2**
2.1 Mammal tree used for simulations . . . 2
2.2 Site-specific fitness profiles (`SimuDiv`) . . . 3
2.3 Wright-Fisher with polymorphism (`SimuPoly`) . . . 10
2.4 Fisher geometric landscape (`SimuGeo`) . . . 17
2.5 Protein folding probability (`SimuFold`) . . . 19
2.6 Identifiability of N_{e} and μ . . . 22

3 Empirical data in mammals **22**
3.1 Chain convergence . . . 23
3.2 Traits estimation & correlation (replicate 1, chain 1) . . . 24
3.3 Repeatability of experiments . . . 29
3.4 Amino-acid preferences entropy . . . 34
3.5 Identifiability of N_{e} and μ . . . 34
3.6 Traits estimation with branch ω (replicate 1, chain 1) . . . 35

4 Empirical data in Isopods **36**
4.1 Traits estimation (replicate 1, chain 1) . . . 37
4.2 Repeatability of experiments . . . 39
4.3 Identifiability of N_{e} and μ . . . 45

5 Empirical data in Primates **45**
5.1 Chain convergence . . . 45
5.2 Traits estimation (chain 1) . . . 46
5.3 Amino-acid preferences entropy . . . 54
5.4 Traits estimation with branch ω (chain 1) . . . 54

6 Sufficient statistics **57**
6.1 Path sufficient statistics . . . 57
6.2 Length sufficient statistics . . . 58
6.3 Scatter sufficient statistics . . . 58

1 Summary statistics

1.1 Partial correlation coefficient

The correlation coefficient $\rho_{a,b}$ give the total regression between two variables. Partial-correlation coefficient account for the entire covariance matrix, and measure the correlation between 2 traits, knowing the values of all the other traits:

$$\rho_{a,b|c\in\{1,\ldots,L\}\setminus\{a,b\}} = -\frac{\Omega_{a,b}}{\sqrt{\Omega_{a,a}\Omega_{b,b}}}, \tag{1}$$

where the precision matrix $\mathbf{\Omega}$ is the inverse of the covariance matrix:

$$\mathbf{\Omega} = \mathbf{\Sigma}^{-1} \tag{2}$$

1.2 Fitness profile entropy

For a category k, the Shannon's entropy (Ω) of the fitness profile ($\boldsymbol{\phi}$) is defined as:

$$\Omega^{(\mathrm{k})} = -\sum_{\mathrm{a}=1}^{20} \phi_{\mathrm{a}}^{(\mathrm{k})} \ln\left(\phi_{\mathrm{a}}^{(\mathrm{k})}\right) \tag{3}$$

The Shannon's entropy measures the flatness of the fitness profile, with a value of 0 corresponding to a single peak fitness landscape (only one amino acid is present), and a value of $log(20) \simeq 3$ corresponding to a neutral landscape, where each amino acid has the same fitness.

The Shannon's entropy can be averaged over all sites as:

$$\langle\Omega\rangle = \frac{1}{\mathrm{Z}}\sum_{\mathrm{z}=1}^{\mathrm{Z}} \Omega^{\kappa(\mathrm{z})} \tag{4}$$

2 Simulations

2.1 Mammal tree used for simulations

```
((((Trichechus_manatus_latirostris:0.035513,(Procavia_capensis:0.084637,Loxodonta_africana
    :0.041516)1:0.003159)1:0.012832,(Orycteropus_afer_afer:0.061819,(Elephantulus_edwardii
    :0.113286,Echinops_telfairi:0.132412)1:0.002801)1:0.003326)1:0.030446,(
    Dasypus_novemcinctus:0.062179,Choloepus_hoffmanni:0.052537)1:0.028553)1:0.0128868,((((
    Sorex_araneus:0.151181,Erinaceus_europaeus:0.132436)1:0.010489,Condylura_cristata
    :0.099251)1:0.014601,((((Rousettus_aegyptiacus:0.02426,Pteropus_vampyrus:0.017977)
    1:0.041808,(Rhinolophus_sinicus:0.033606,Hipposideros_armiger:0.034245)1:0.022424)
    1:0.005466,(Miniopterus_natalensis:0.048559,(Myotis_lucifugus:0.02061,Eptesicus_fuscus
    :0.020745)1:0.032909)1:0.026648)1:0.014977,(((Equus_caballus:0.040208,
    Ceratotherium_simum_simum:0.031375)1:0.01757,((Vicugna_pacos:0.01123,Camelus_bactrianus
    :0.007435)1:0.048955,(Sus_scrofa:0.061861,((Odocoileus_virginianus_texanus:0.022142,(
    Bos_taurus:0.015258,(Ovis_aries:0.007108,Capra_hircus:0.005201)1:0.01242)1:0.005885)
    1:0.046478,((Physeter_catodon:0.012098,(Lipotes_vexillifer:0.010043,((Tursiops_truncatus
    :0.004507,Orcinus_orca:0.002912)1:0.003737,Delphinapterus_leucas:0.005948)1:0.00282)
    1:0.004819)1:0.001789,Balaenoptera_acutorostrata_scammoni:0.011414)1:0.021555)1:0.008733)
    1:0.00488)1:0.02127)1:0.00171,((Canis_familiaris:0.045402,((Ursus_maritimus:0.009802,
    Ailuropoda_melanoleuca:0.01071)1:0.019944,(Mustela_putorius:0.014168,
    Enhydra_lutris_kenyoni:0.010979)1:0.033406)1:0.008825)1:0.009892,(Panthera_pardus
    :0.004889,(Acinonyx_jubatus:0.005956,Felis_catus:0.004223)1:0.001612)1:0.040037)
    1:0.028291)1:0.001352)1:0.00366)1:0.011854,((Tupaia_belangeri:0.09974,((
    Oryctolagus_cuniculus:0.057311,Ochotona_princeps:0.105655)1:0.054285,((
    Marmota_marmota_marmota:0.007619,Ictidomys_tridecemlineatus:0.00925)1:0.071266,((((
    Octodon_degus:0.060538,Chinchilla_lanigera:0.038902)1:0.006926,Cavia_porcellus:0.06173)
    1:0.012433,Heterocephalus_glaber:0.049792)1:0.058724,((Jaculus_jaculus:0.103095,(((
    Rattus_norvegicus:0.042005,Mus_musculus:0.03846)1:0.026492,Meriones_unguiculatus:0.06177)
    1:0.007886,(Peromyscus_maniculatus:0.043119,(Microtus_ochrogaster:0.051557,
    Mesocricetus_auratus:0.048603)1:0.002904)1:0.012733)1:0.068248)1:0.022736,(Dipodomys_ordii
    :0.109334,Castor_canadensis:0.067389)1:0.010586)1:0.009351)1:0.002793)1:0.013804)
    1:0.005752)1:0.001773,((Otolemur_garnettii:0.068772,(Propithecus_coquereli:0.020483,
    Microcebus_murinus:0.028823)1:0.018976)1:0.01813,(((Nomascus_leucogenys:0.011348,(
    Pongo_abelii:0.009387,((Pan_troglodytes:0.003287,Homo_sapiens:0.003197)1:0.000928,
    Gorilla_gorilla:0.004417)1:0.004317)1:0.00142)1:0.005095,((Chlorocebus_sabaeus:0.005997,(
    Macaca_mulatta:0.003914,(Papio_anubis:0.003289,(Mandrillus_leucophaeus:0.00288,
    Cercocebus_atys:0.003073)1:0.000625)1:0.000757)1:0.001692)1:0.002705,Colobus_angolensis
    :0.009421)1:0.010106)1:0.009753,((Saimiri_boliviensis:0.014318,Cebus_capucinus:0.012274)
    1:0.001913,(Callithrix_jacchus:0.017752,Aotus_nancymaae:0.012014)1:0.000671)1:0.019849)
    1:0.037902)1:0.009114)1:0.01138)1:0.00067825);
```

The tree has been extracted from OrthoMam database (Ranwez *et al.*, 2007; Scornavacca *et al.*, 2019). This is a subset of 77 species for which information is available for at least one life-history traits in the AnAge database (De Magalhães and Costa, 2009; Tacutu *et al.*, 2013). The longest distance between root to leaf is used to set the root age, since this tree is not ultrametric.

2.2 Site-specific fitness profiles (SimuDiv)

For simulations under a site-independent fitness landscape, with site-specific fitness profiles, the protein log-fitness is computed as the sum of amino-acid log-fitness coefficients along the sequence. In this model, for each codon site z, we assign a fitness profile, denoted $\boldsymbol{\phi}^{(\mathrm{z})} = \{\phi_{\mathrm{a}}^{(\mathrm{z})},\ 1 \leq \mathrm{a} \leq 20\}$, a vector of 20 amino-acid scaled (Wrightian) fitness coefficients. Since $\mathbb{S}[\mathrm{z}]$ is the codon at site z, the encoded amino acid is $\mathcal{A}(\mathbb{S}[\mathrm{z}])$, hence the fitness at site z is $\boldsymbol{\phi}^{(\mathrm{z})}_{\mathcal{A}(\mathbb{S}[\mathrm{z}])}$. Altogether, the selection coefficient of the mutant $\mathbb{S}'$ is:

$$s\left(\mathbb{S}, \mathbb{S}'\right) = \sum_{\mathrm{z}=1}^{\mathrm{Z}} \ln \left(\frac{\boldsymbol{\phi}^{(\mathrm{z})}_{\mathcal{A}(\mathbb{S}'[\mathrm{z}])}}{\boldsymbol{\phi}^{(\mathrm{z})}_{\mathcal{A}(\mathbb{S}[\mathrm{z}])}} \right), \tag{5}$$

The fitness vectors $\boldsymbol{\phi}^{(\mathrm{z})}$ used in this study are extracted from Bloom (2017), which were experimentally determined by deep mutational scanning for 498 codon sites of the nucleoprotein in *Influenza Virus* strains (as human host). Although the *Influenza Virus* is phylogenetically far away from vertebrates, the experimentally determined profiles in these strains capture the structural constrains exerted on proteins. We subsampled randomly 30 fitness profiles out of the 498 profiles experimentally determined, then for each codon site z of our simulation, we assign randomly one the 30 fitness profile (sampling with replacement), which altogether determines the selection coefficient for any non-synonymous mutation.

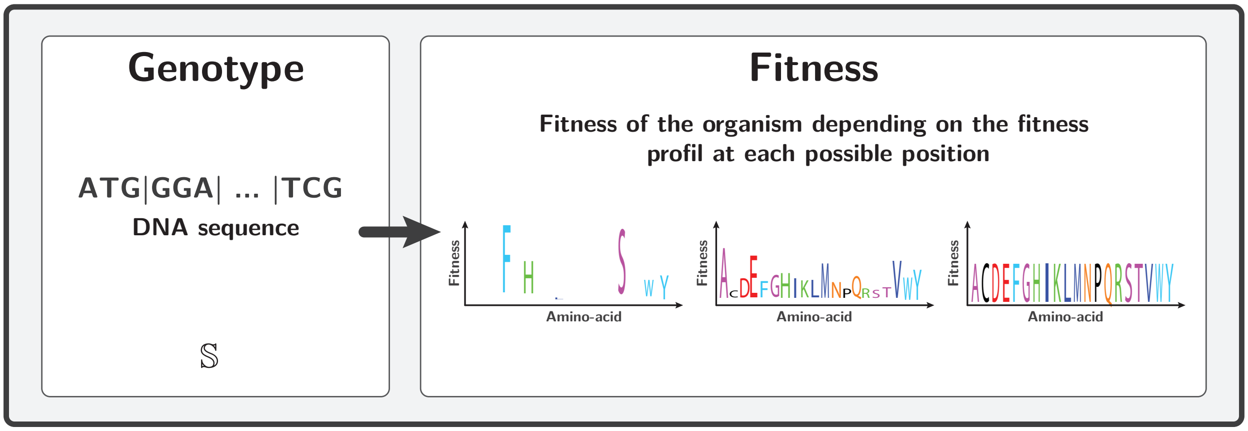

The next change in the protein coding DNA and the time to next the event is chosen using Gillespie's algorithm (Gillespie, 1977), according to the rates of substitution between codons:

$$Q_{i,j} = \mu_{i,j} \frac{4N_{\mathrm{e}} s\left(\mathbb{S}^t, \mathbb{S}^{t+1}\right)}{1 - \mathrm{e}^{-4N_{\mathrm{e}} s\left(\mathbb{S}^t, \mathbb{S}^{t+1}\right)}}, \tag{6}$$

where $Q_{i,j} = \mu_{i,j}$ in the case of synonymous substitutions.

**Parameter**	Value
Mutation rate (at the root)	$u = 1.0 \times 10^{-8}$ per site per generation
Root age	150 Million years
Generation time (at the root)	$\tau = 10$ years
Effective population size (at the root)	$N_{\mathrm{e}} = 1.0$ (for identifiability of fitness profiles)
Number of codon sites	$5,000$
Exon size (number of codon sites)	300
Random seed	65489
Branch-wise correlation	True

Table 1: Parameters used for `SimuDiv`. The configuration files and scripts to produce simulations are available as `config.yaml` at https://github.com/ThibaultLatrille/MutationSelectionDrift.

The input nucleotide mutation matrix ($\boldsymbol{R}$) is a symmetric time-reversible matrix, which is automatically normalized by the simulator:

$$\boldsymbol{R} = \begin{array}{c} \\ A \\ C \\ G \\ T \end{array} \begin{array}{c} \begin{array}{cccc} A & C & G & T \end{array} \\ \begin{pmatrix} - & 1 & 1 & 1 \\ 1 & - & 1 & 1 \\ 1 & 1 & - & 1 \\ 1 & 1 & 1 & - \end{pmatrix} \end{array} \tag{7}$$

The input precision matrix ($\boldsymbol{\Omega} = \boldsymbol{\Sigma}^{-1}$) of the multivariate geometric Brownian is composed of three process, namely the effective population size (N_{e}), mutation rate per generation per site (u) and generation time (τ). The covariance matrix ($\boldsymbol{\Sigma}$) is computed from the input precision matrix ($\boldsymbol{\Omega}$) by the simulator, and represents the covariances of traits along the tree (from root to leaves).

$$\boldsymbol{\Omega} = \begin{array}{c} \\ N_{\mathrm{e}} \\ u \\ \tau \end{array} \begin{array}{c} \begin{array}{ccc} N_{\mathrm{e}} & u & \tau \end{array} \\ \begin{pmatrix} 0.5 & 0 & 0 \\ 0 & 2.0 & 0 \\ 0 & 0 & 1.0 \end{pmatrix} \end{array} \tag{8}$$

Site-specific fitness profiles used as input of the simulator are shown as logo plot:

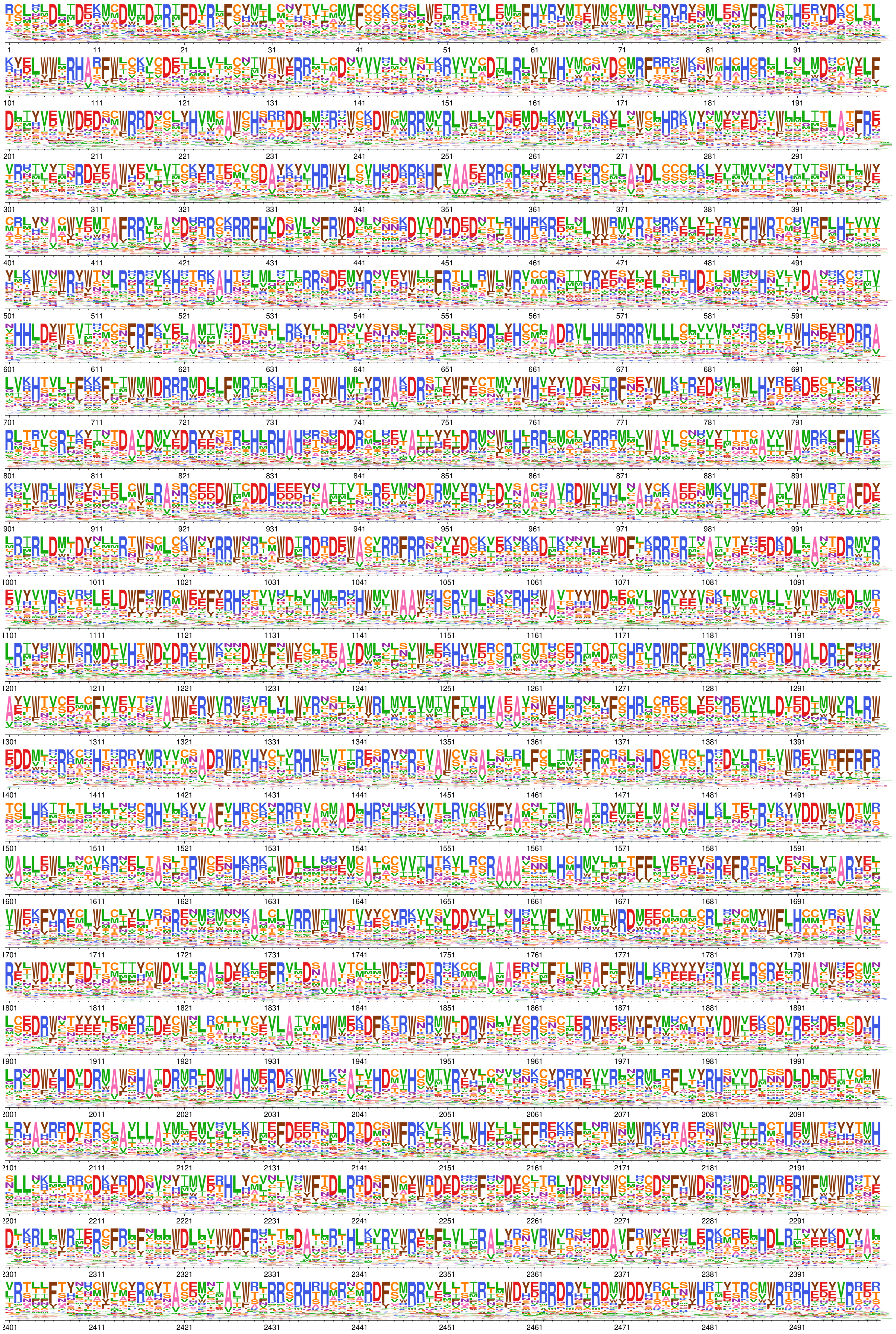

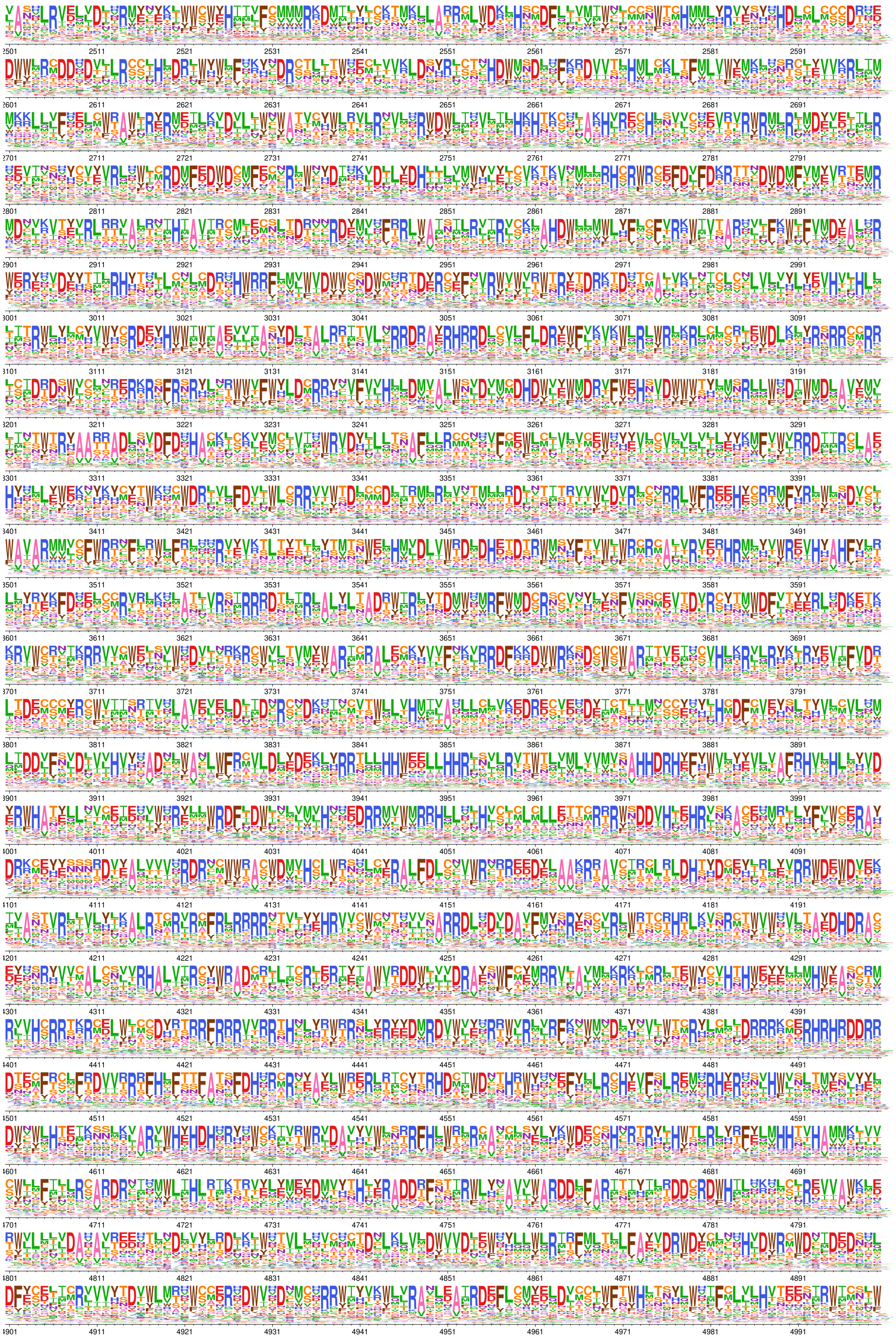

2501 2511 2521 2531 2541 2551 2561 2571 2581 2591
2601 2611 2621 2631 2641 2651 2661 2671 2681 2691
2701 2711 2721 2731 2741 2751 2761 2771 2781 2791
2801 2811 2821 2831 2841 2851 2861 2871 2881 2891
2901 2911 2921 2931 2941 2951 2961 2971 2981 2991
3001 3011 3021 3031 3041 3051 3061 3071 3081 3091
3101 3111 3121 3131 3141 3151 3161 3171 3181 3191
3201 3211 3221 3231 3241 3251 3261 3271 3281 3291
3301 3311 3321 3331 3341 3351 3361 3371 3381 3391
3401 3411 3421 3431 3441 3451 3461 3471 3481 3491
3501 3511 3521 3531 3541 3551 3561 3571 3581 3591
3601 3611 3621 3631 3641 3651 3661 3671 3681 3691
3701 3711 3721 3731 3741 3751 3761 3771 3781 3791
3801 3811 3821 3831 3841 3851 3861 3871 3881 3891
3901 3911 3921 3931 3941 3951 3961 3971 3981 3991
4001 4011 4021 4031 4041 4051 4061 4071 4081 4091
4101 4111 4121 4131 4141 4151 4161 4171 4181 4191
4201 4211 4221 4231 4241 4251 4261 4271 4281 4291
4301 4311 4321 4331 4341 4351 4361 4371 4381 4391
4401 4411 4421 4431 4441 4451 4461 4471 4481 4491
4501 4511 4521 4531 4541 4551 4561 4571 4581 4591
4601 4611 4621 4631 4641 4651 4661 4671 4681 4691
4701 4711 4721 4731 4741 4751 4761 4771 4781 4791
4801 4811 4821 4831 4841 4851 4861 4871 4881 4891
4901 4911 4921 4931 4941 4951 4961 4971 4981 4991

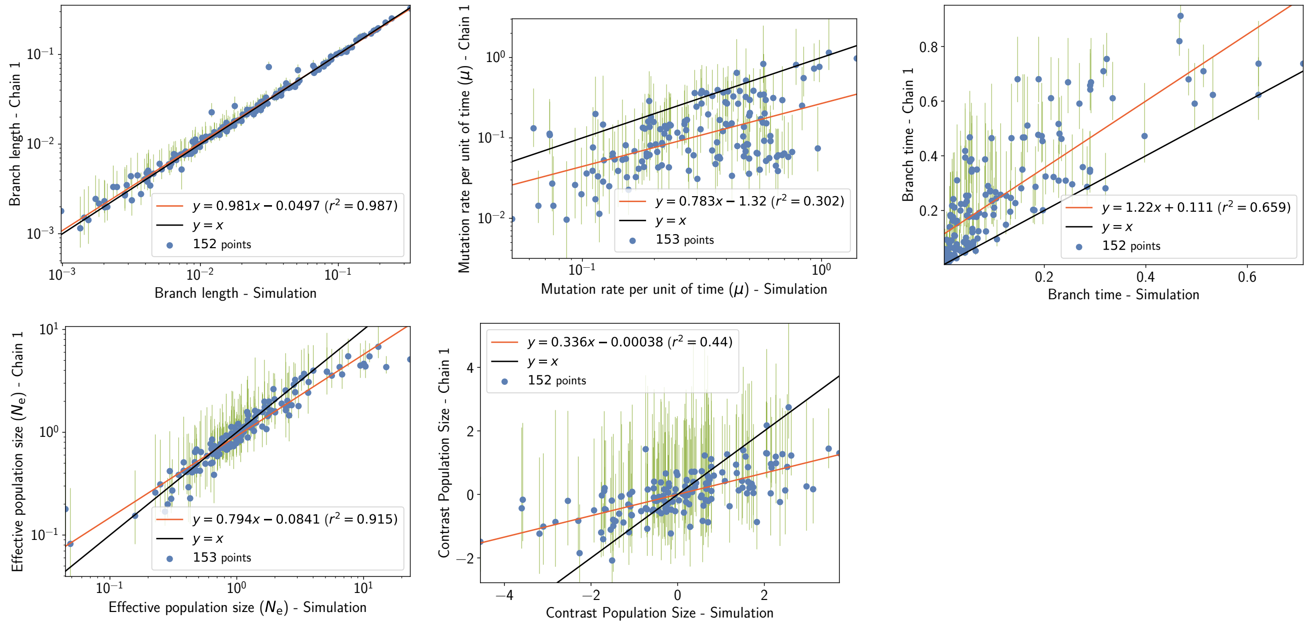

Figure 1: Inferred branch parameters under simulations accounting for site-specific amino-acid profiles, long term fluctuation of N_{e}, mutation rate per generation and generation time. Estimation is obtained with the mechanistic inference model developed in this paper of site-specific amino-acid fitness profiles and log-Brownian process for N_{e}, μ and life-history traits.

**Experiment**	$\langle\Omega\rangle$ (**branch** N_{e})	$\langle\Omega\rangle$ (**constant** N_{e})
`SimuDiv`, chain 1	2.30 ± 0.04	2.45 ± 0.02
`SimuDiv`, chain 2	2.30 ± 0.04	2.45 ± 0.02

Table 2: Estimated amino-acid entropy under simulations accounting for long term fluctuation of N_{e}, mutation rate per generation and generation time. Estimation is obtained with the mechanistic inference model developed in this paper of site-specific amino-acid fitness profiles and log-Brownian process for N_{e}, μ and life-history traits (in the left column), or under the assumption of constant N_{e} (in the right column).

Site-specific estimated fitness profiles (chain 1) are shown as logo plot:

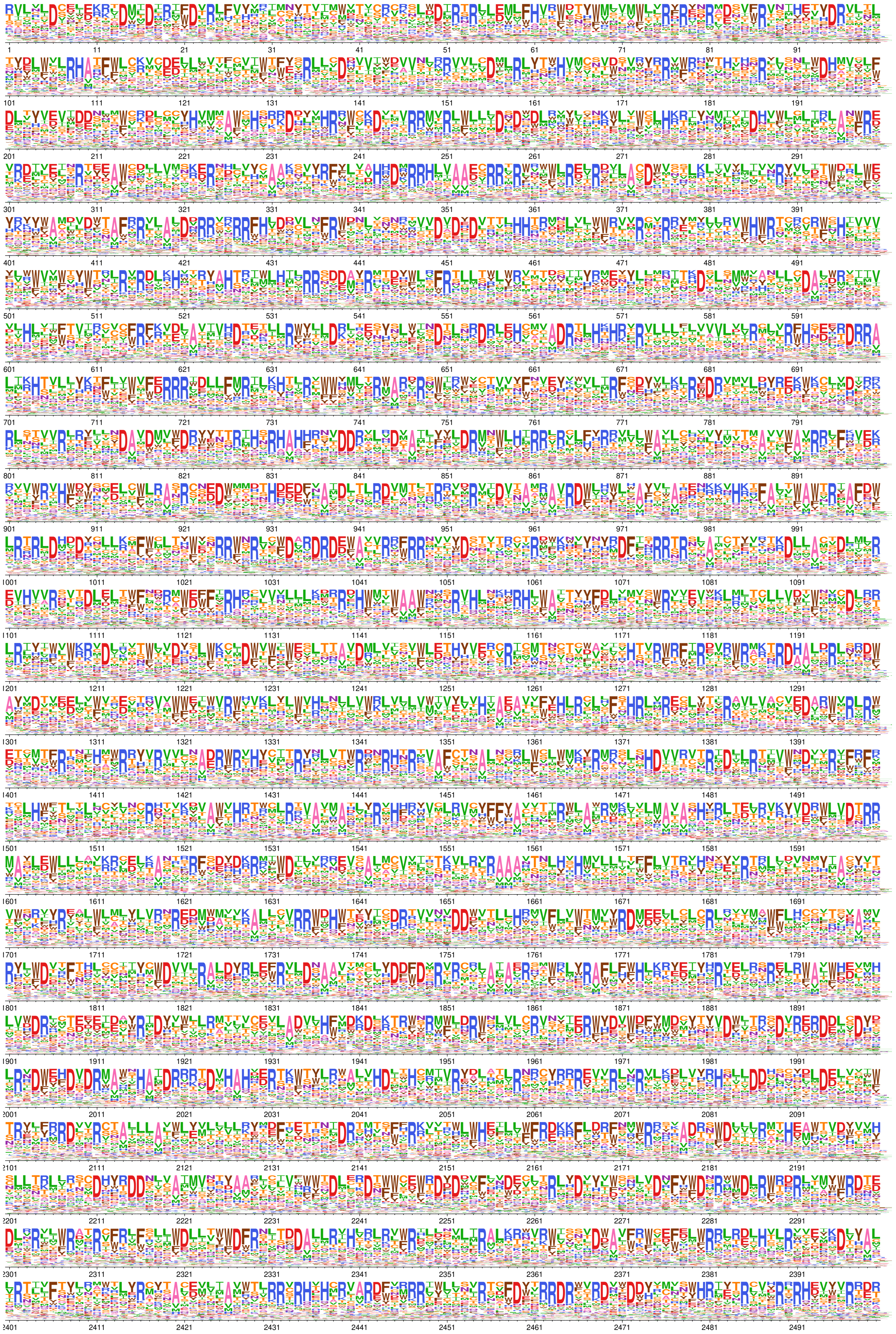
1 11 21 31 41 51 61 71 81 91
101 111 121 131 141 151 161 171 181 191
201 211 221 231 241 251 261 271 281 291
301 311 321 331 341 351 361 371 381 391
401 411 421 431 441 451 461 471 481 491
501 511 521 531 541 551 561 571 581 591
601 611 621 631 641 651 661 671 681 691
701 711 721 731 741 751 761 771 781 791
801 811 821 831 841 851 861 871 881 891
901 911 921 931 941 951 961 971 981 991
1001 1011 1021 1031 1041 1051 1061 1071 1081 1091
1101 1111 1121 1131 1141 1151 1161 1171 1181 1191
1201 1211 1221 1231 1241 1251 1261 1271 1281 1291
1301 1311 1321 1331 1341 1351 1361 1371 1381 1391
1401 1411 1421 1431 1441 1451 1461 1471 1481 1491
1501 1511 1521 1531 1541 1551 1561 1571 1581 1591
1601 1611 1621 1631 1641 1651 1661 1671 1681 1691
1701 1711 1721 1731 1741 1751 1761 1771 1781 1791
1801 1811 1821 1831 1841 1851 1861 1871 1881 1891
1901 1911 1921 1931 1941 1951 1961 1971 1981 1991
2001 2011 2021 2031 2041 2051 2061 2071 2081 2091
2101 2111 2121 2131 2141 2151 2161 2171 2181 2191
2201 2211 2221 2231 2241 2251 2261 2271 2281 2291
2301 2311 2321 2331 2341 2351 2361 2371 2381 2391
2401 2411 2421 2431 2441 2451 2461 2471 2481 2491

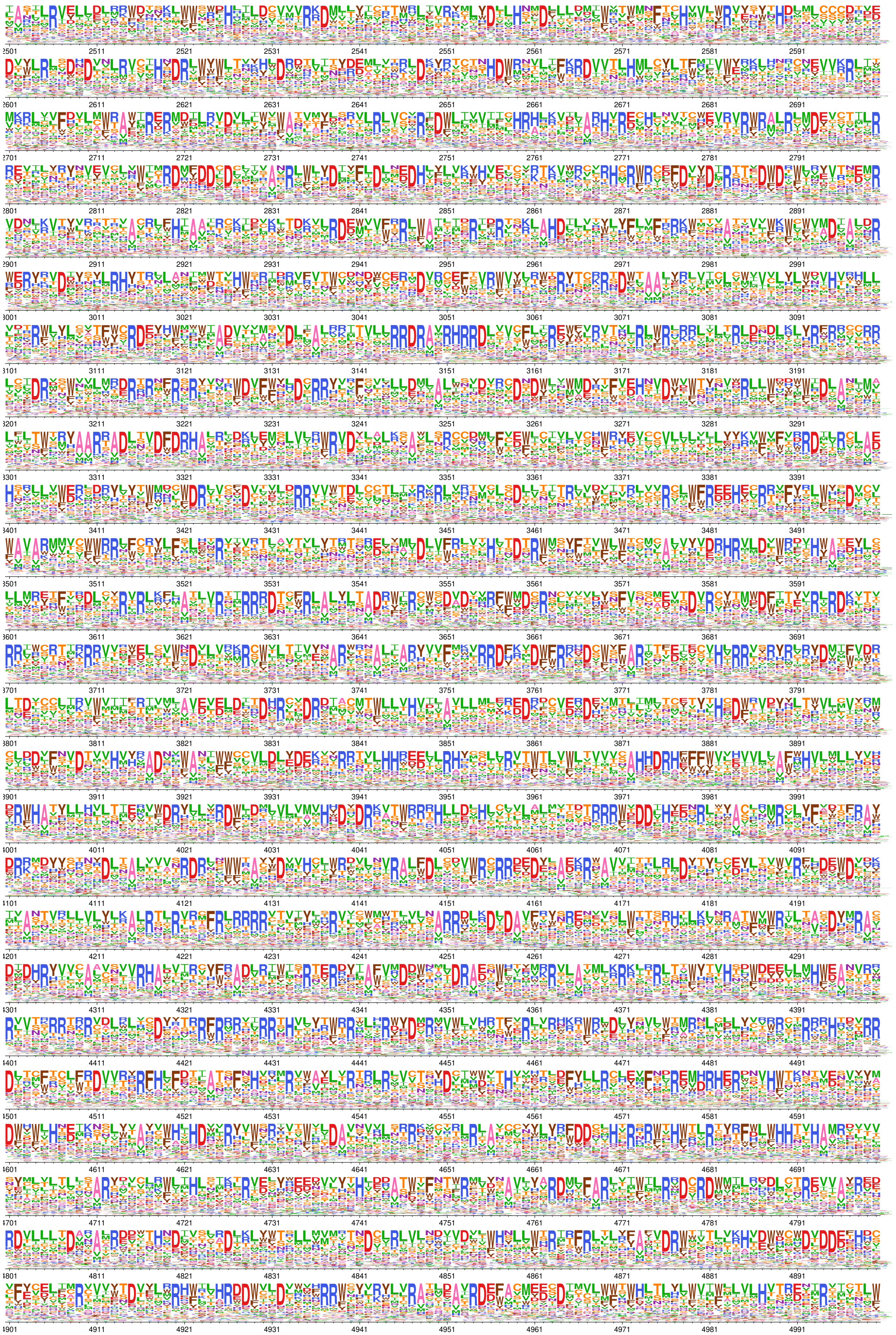

2501 2511 2521 2531 2541 2551 2561 2571 2581 2591
2601 2611 2621 2631 2641 2651 2661 2671 2681 2691
2701 2711 2721 2731 2741 2751 2761 2771 2781 2791
2801 2811 2821 2831 2841 2851 2861 2871 2881 2891
2901 2911 2921 2931 2941 2951 2961 2971 2981 2991
3001 3011 3021 3031 3041 3051 3061 3071 3081 3091
3101 3111 3121 3131 3141 3151 3161 3171 3181 3191
3201 3211 3221 3231 3241 3251 3261 3271 3281 3291
3301 3311 3321 3331 3341 3351 3361 3371 3381 3391
3401 3411 3421 3431 3441 3451 3461 3471 3481 3491
3501 3511 3521 3531 3541 3551 3561 3571 3581 3591
3601 3611 3621 3631 3641 3651 3661 3671 3681 3691
3701 3711 3721 3731 3741 3751 3761 3771 3781 3791
3801 3811 3821 3831 3841 3851 3861 3871 3881 3891
3901 3911 3921 3931 3941 3951 3961 3971 3981 3991
4001 4011 4021 4031 4041 4051 4061 4071 4081 4091
4101 4111 4121 4131 4141 4151 4161 4171 4181 4191
4201 4211 4221 4231 4241 4251 4261 4271 4281 4291
4301 4311 4321 4331 4341 4351 4361 4371 4381 4391
4401 4411 4421 4431 4441 4451 4461 4471 4481 4491
4501 4511 4521 4531 4541 4551 4561 4571 4581 4591
4601 4611 4621 4631 4641 4651 4661 4671 4681 4691
4701 4711 4721 4731 4741 4751 4761 4771 4781 4791
4801 4811 4821 4831 4841 4851 4861 4871 4881 4891
4901 4911 4921 4931 4941 4951 4961 4971 4981 4991

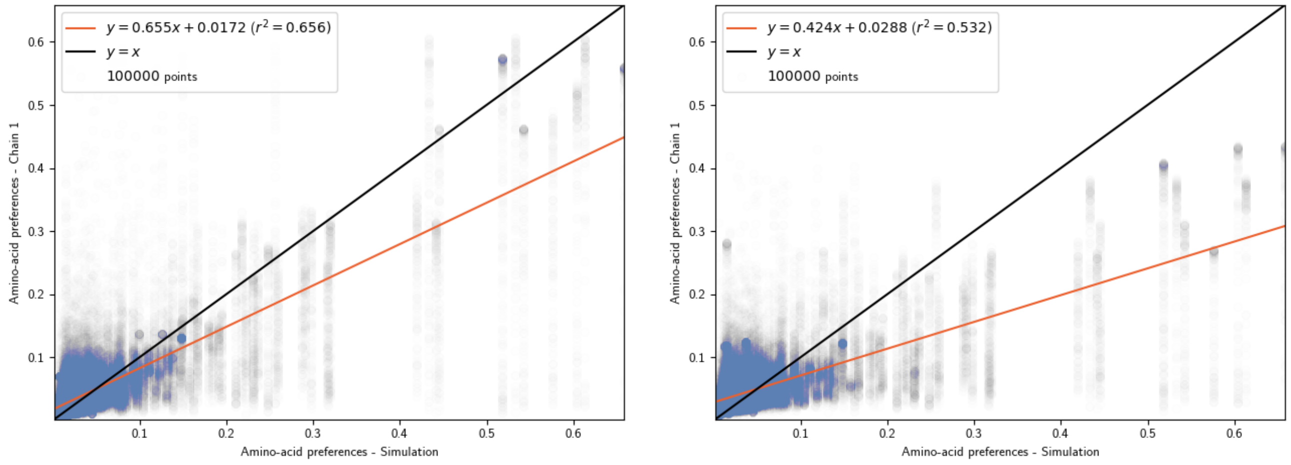

Figure 2: Inferred and simulated site-specific amino-acid profiles under simulation accounting for long term fluctuation of N_{e}, mutation rate per generation and generation time. Estimation is obtained with the mechanistic inference model developed in this paper of site-specific amino-acid fitness profiles and log-Brownian process for N_{e}, μ and life-history traits (in the left panel), or under the assumption of constant N_{e} (in the right panel).

2.3 Wright-Fisher with polymorphism (SimuPoly)

The evolutionary dynamics was formalized as a Wright-Fisher model with mutation, selection and drift The population is assumed to be panmictic, with effective population size N_{e} and with non-overlapping generations.

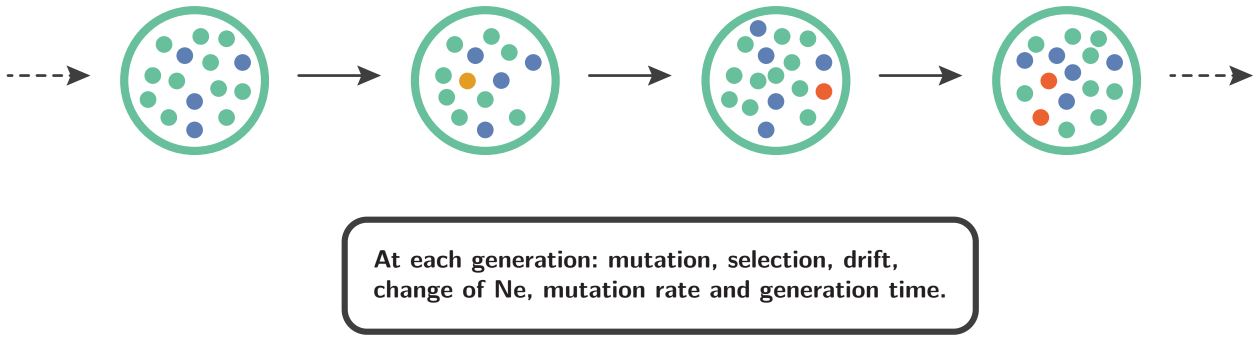

For simulations under a site-independent fitness landscape, with site-specific fitness profiles, the protein log-fitness is computed as the sum of amino-acid log-fitness coefficients along the sequence. In this model, for each codon site z, we assign a fitness profile, denoted $\boldsymbol{\phi}^{(\mathrm{z})} = \{\phi_{\mathrm{a}}^{(\mathrm{z})},\ 1 \leq \mathrm{a} \leq 20\}$, a vector of 20 amino-acid scaled (Wrightian) fitness coefficients. Since $\mathbb{S}[\mathrm{z}]$ is the codon at site z, the encoded amino acid is $\mathcal{A}(\mathbb{S}[\mathrm{z}])$, hence the fitness at site z is $\phi_{\mathcal{A}(\mathbb{S}[\mathrm{z}])}^{(\mathrm{z})}$. Altogether, the selection coefficient of the mutant $\mathbb{S}'$ is:

$$s(\mathbb{S}, \mathbb{S}') = \sum_{\mathrm{z}=1}^{\mathrm{Z}} \ln \left(\frac{\phi_{\mathcal{A}(\mathbb{S}'[\mathrm{z}])}^{(\mathrm{z})}}{\phi_{\mathcal{A}(\mathbb{S}[\mathrm{z}])}^{(\mathrm{z})}} \right), \tag{9}$$

The fitness vectors $\boldsymbol{\phi}^{(\mathrm{z})}$ used in this study are extracted from Bloom (2017), which were experimentally determined by deep mutational scanning for 498 codon sites of the nucleoprotein in *Influenza Virus* strains (as human host). Although the *Influenza Virus* is phylogenetically far away from vertebrates, the experimentally determined profiles in these strains capture the structural constrains exerted on proteins. For each codon site z of our simulation, we assign randomly one the 498 fitness profile (sampling with replacement) experimentally determined, which altogether determines the selection coefficient for any non-synonymous mutation.

**Parameter**	Value
Mutation rate (at the root)	$u = 1.0 \times 10^{-8}$ per site per generation
Root age	150 Million years
Generation time (at the root)	$\tau = 10$ years
Number of codon sites	$5,000$
Exon size (number of codon sites)	300
Random seed	65489
Effective population size (at the root)	$N_{\text{e}} = 5,000$
σ parameter for Ornstein-Uhlenbeck process	0.2
θ parameter for Ornstein-Uhlenbeck process	0.2

Table 3: Parameters used for `SimuPoly`. The configuration files and scripts to produce simulations are available as `config.yaml` at https://github.com/ThibaultLatrille/MutationSelectionDrift.

The input nucleotide mutation matrix ($\boldsymbol{R}$) is a symmetric time-reversible matrix, which is automatically normalized by the simulator:

$$\boldsymbol{R} = \begin{array}{c} \\ A \\ C \\ G \\ T \end{array} \begin{array}{c} \begin{array}{cccc} A & C & G & T \end{array} \\ \begin{pmatrix} - & 1 & 1 & 1 \\ 1 & - & 1 & 1 \\ 1 & 1 & - & 1 \\ 1 & 1 & 1 & - \end{pmatrix} \end{array} \tag{10}$$

The input precision matrix ($\boldsymbol{\Omega} = \boldsymbol{\Sigma}^{-1}$) of the multivariate geometric Brownian is composed of three process, namely the effective population size (N_{e}), mutation rate per generation per site (u) and generation time (τ). The covariance matrix ($\boldsymbol{\Sigma}$) is computed from the input precision matrix ($\boldsymbol{\Omega}$) by the simulator, and represents the covariances of traits along the tree (from root to leaves).

$$\boldsymbol{\Omega} = \begin{array}{c} \\ N_{\text{e}} \\ u \\ \tau \end{array} \begin{array}{c} \begin{array}{ccc} N_{\text{e}} & u & \tau \end{array} \\ \begin{pmatrix} 0.5 & 0 & 0 \\ 0 & 2.0 & 0 \\ 0 & 0 & 1.0 \end{pmatrix} \end{array} \tag{11}$$

Site-specific fitness profiles used as input of the simulator are shown as logo plot:

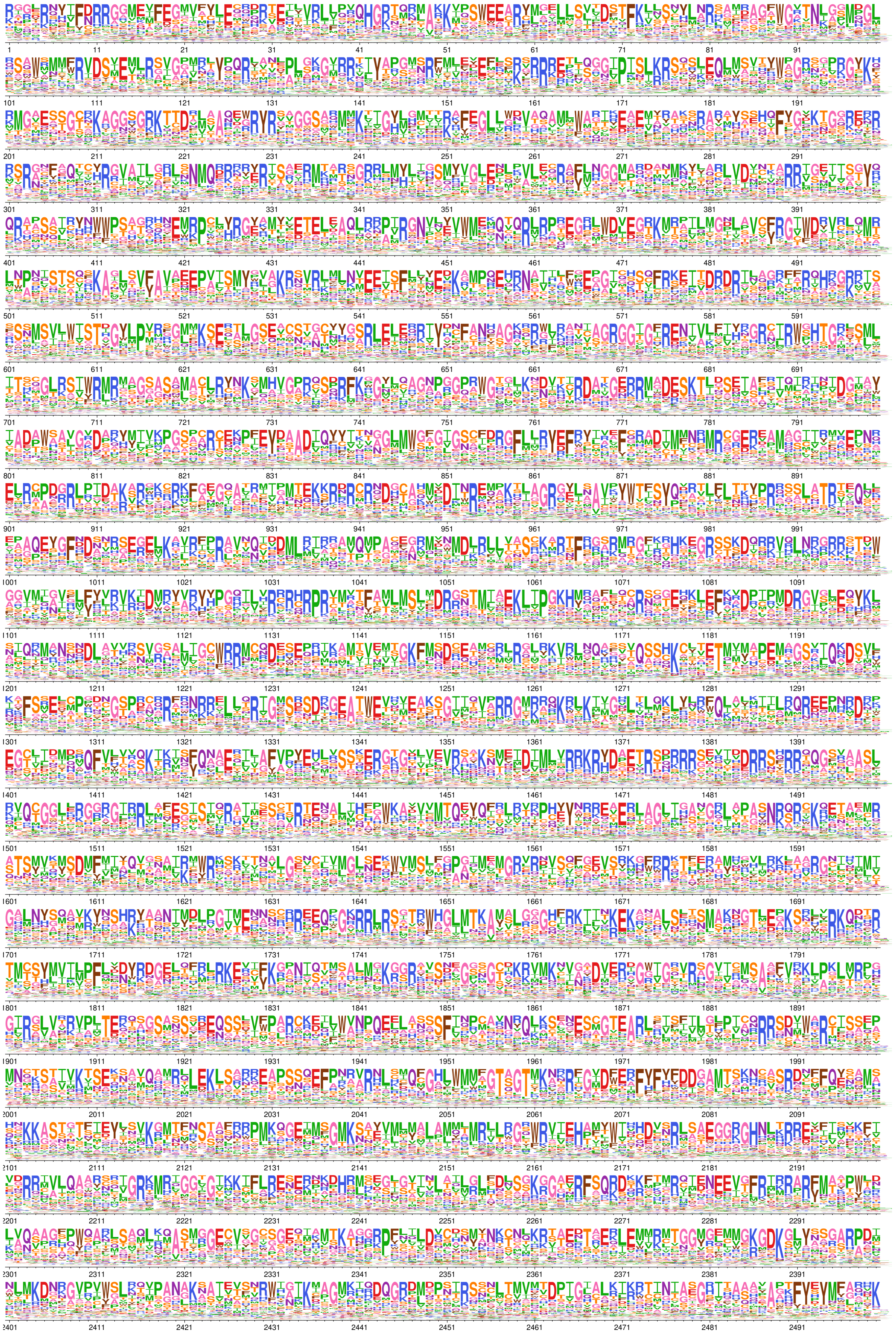

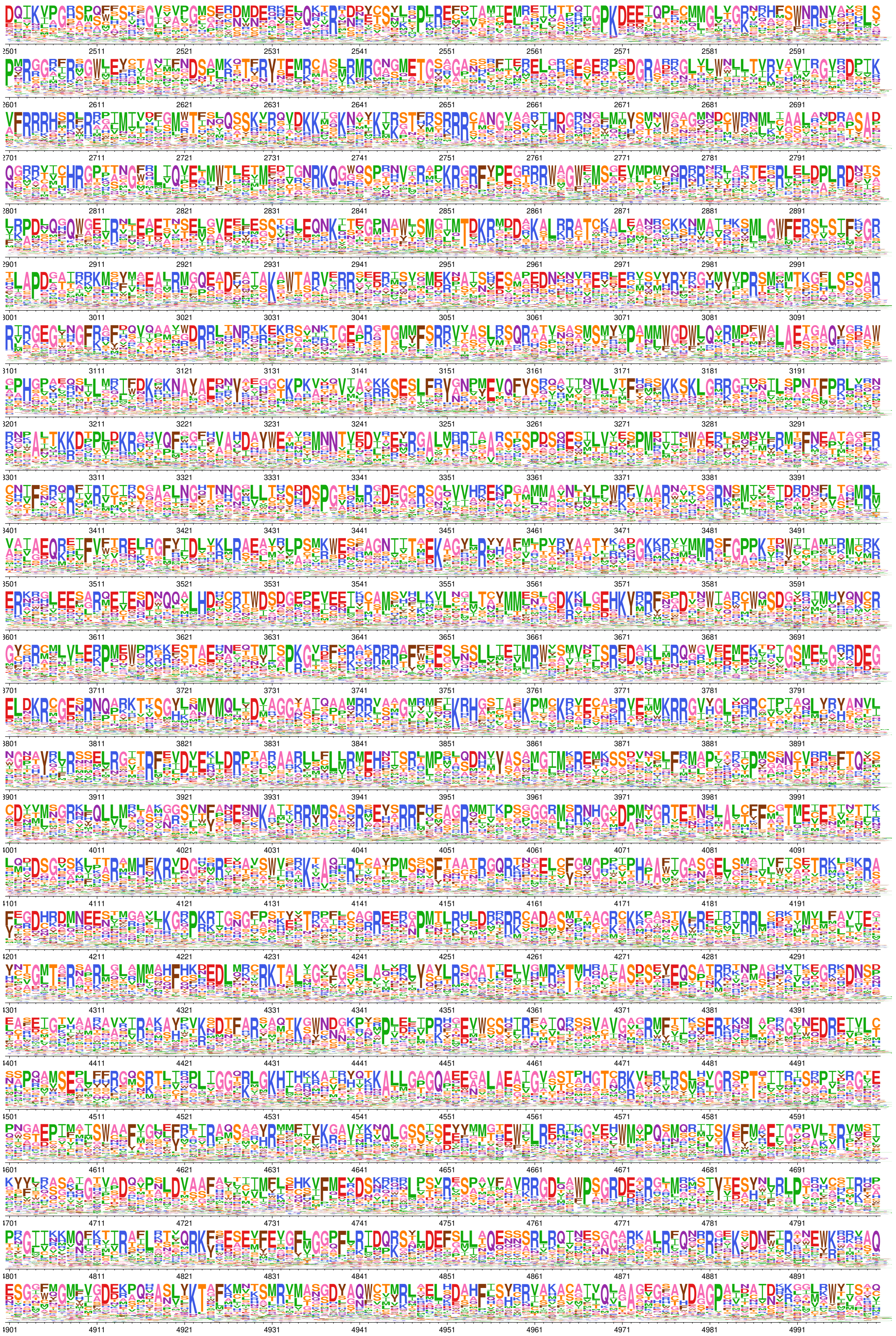
2501 2511 2521 2531 2541 2551 2561 2571 2581 2591
2601 2611 2621 2631 2641 2651 2661 2671 2681 2691
2701 2711 2721 2731 2741 2751 2761 2771 2781 2791
2801 2811 2821 2831 2841 2851 2861 2871 2881 2891
2901 2911 2921 2931 2941 2951 2961 2971 2981 2991
3001 3011 3021 3031 3041 3051 3061 3071 3081 3091
3101 3111 3121 3131 3141 3151 3161 3171 3181 3191
3201 3211 3221 3231 3241 3251 3261 3271 3281 3291
3301 3311 3321 3331 3341 3351 3361 3371 3381 3391
3401 3411 3421 3431 3441 3451 3461 3471 3481 3491
3501 3511 3521 3531 3541 3551 3561 3571 3581 3591
3601 3611 3621 3631 3641 3651 3661 3671 3681 3691
3701 3711 3721 3731 3741 3751 3761 3771 3781 3791
3801 3811 3821 3831 3841 3851 3861 3871 3881 3891
3901 3911 3921 3931 3941 3951 3961 3971 3981 3991
4001 4011 4021 4031 4041 4051 4061 4071 4081 4091
4101 4111 4121 4131 4141 4151 4161 4171 4181 4191
4201 4211 4221 4231 4241 4251 4261 4271 4281 4291
4301 4311 4321 4331 4341 4351 4361 4371 4381 4391
4401 4411 4421 4431 4441 4451 4461 4471 4481 4491
4501 4511 4521 4531 4541 4551 4561 4571 4581 4591
4601 4611 4621 4631 4641 4651 4661 4671 4681 4691
4701 4711 4721 4731 4741 4751 4761 4771 4781 4791
4801 4811 4821 4831 4841 4851 4861 4871 4881 4891
4901 4911 4921 4931 4941 4951 4961 4971 4981 4991

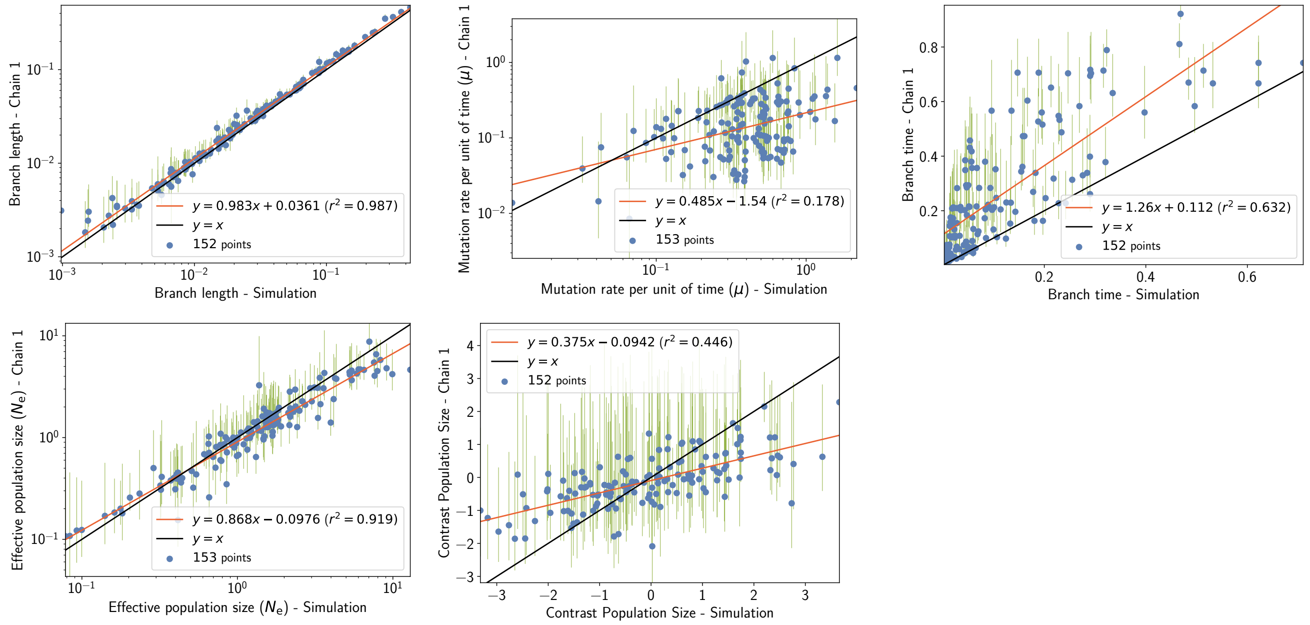

Figure 3: Inferred branch parameters under simulation accounting for finite population effects, site linkage and short term fluctuation of N_{e}. Estimation is obtained with the mechanistic inference model developed in this paper of site-specific amino-acid fitness profiles and log-Brownian process for N_{e}, μ and life-history traits.

**Experiment**	$\langle \Omega \rangle$ **(branch** N_{e}**)**	$\langle \Omega \rangle$ **(constant** N_{e}**)**
`SimuPoly`, chain 1	2.47 ± 0.03	2.37 ± 0.02
`SimuPoly`, chain 2	2.47 ± 0.03	2.37 ± 0.02

Table 4: Estimated amino-acid entropy under simulation accounting for finite population effects, site linkage and short term fluctuation of N_{e}. Estimation is obtained with the mechanistic inference model developed in this paper of site-specific amino-acid fitness profiles and log-Brownian process for N_{e}, μ and life-history traits (in the left column), or under the assumption of constant N_{e} (in the right column).

Site-specific estimated fitness profiles (chain 1) are shown as logo plot:

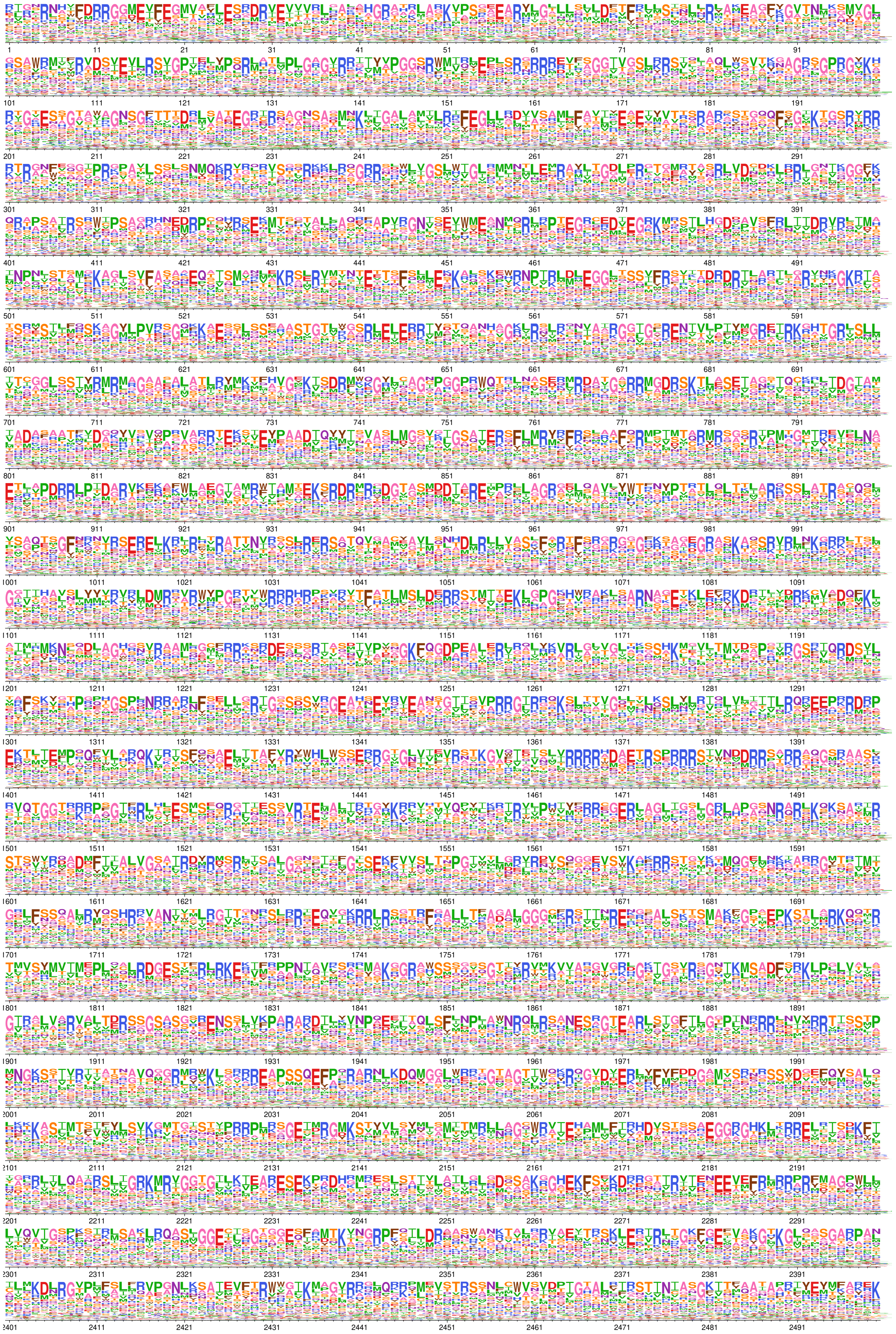

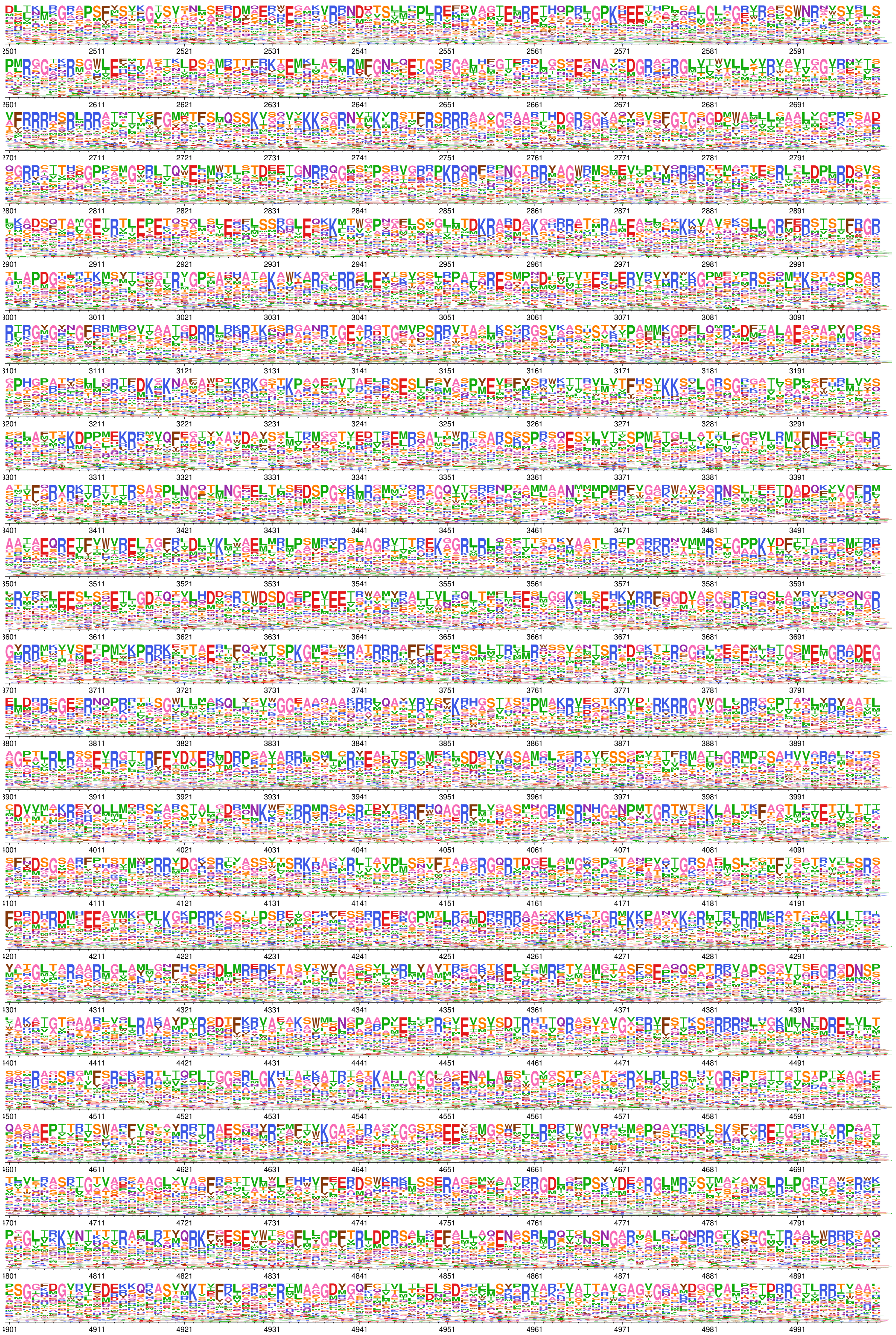
2501 2511 2521 2531 2541 2551 2561 2571 2581 2591
2601 2611 2621 2631 2641 2651 2661 2671 2681 2691
2701 2711 2721 2731 2741 2751 2761 2771 2781 2791
2801 2811 2821 2831 2841 2851 2861 2871 2881 2891
2901 2911 2921 2931 2941 2951 2961 2971 2981 2991
3001 3011 3021 3031 3041 3051 3061 3071 3081 3091
3101 3111 3121 3131 3141 3151 3161 3171 3181 3191
3201 3211 3221 3231 3241 3251 3261 3271 3281 3291
3301 3311 3321 3331 3341 3351 3361 3371 3381 3391
3401 3411 3421 3431 3441 3451 3461 3471 3481 3491
3501 3511 3521 3531 3541 3551 3561 3571 3581 3591
3601 3611 3621 3631 3641 3651 3661 3671 3681 3691
3701 3711 3721 3731 3741 3751 3761 3771 3781 3791
3801 3811 3821 3831 3841 3851 3861 3871 3881 3891
3901 3911 3921 3931 3941 3951 3961 3971 3981 3991
4001 4011 4021 4031 4041 4051 4061 4071 4081 4091
4101 4111 4121 4131 4141 4151 4161 4171 4181 4191
4201 4211 4221 4231 4241 4251 4261 4271 4281 4291
4301 4311 4321 4331 4341 4351 4361 4371 4381 4391
4401 4411 4421 4431 4441 4451 4461 4471 4481 4491
4501 4511 4521 4531 4541 4551 4561 4571 4581 4591
4601 4611 4621 4631 4641 4651 4661 4671 4681 4691
4701 4711 4721 4731 4741 4751 4761 4771 4781 4791
4801 4811 4821 4831 4841 4851 4861 4871 4881 4891
4901 4911 4921 4931 4941 4951 4961 4971 4981 4991

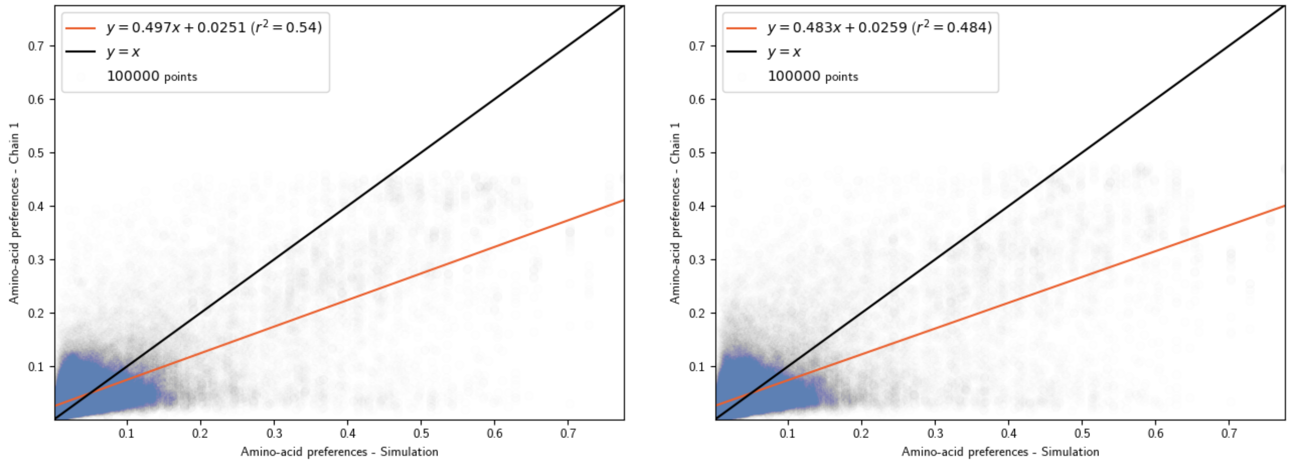

Figure 4: Inferred and simulated site-specific amino-acid profiles under simulation accounting for finite population effects, site linkage and short term fluctuation of N_{e}. Estimation is obtained with the mechanistic inference model developed in this paper of site-specific amino-acid fitness profiles and log-Brownian process for N_{e}, μ and life-history traits (in the left panel), or under the assumption of constant N_{e} (in the right panel).

2.4 Fisher geometric landscape (`SimuGeo`)

We simulated substitutions in a protein using an adaptation of Fisher's geometric landscape (Tenaillon, 2014; Blanquart and Bataillon, 2016). In the original context, the phenotype is a vector ($\boldsymbol{P}$) in a multidimensional space, where the number of dimensions is often termed complexity. From a phenotype, the fitness is a monotonously decreasing function of the phenotype distance to 0. The exact functional phenotype-fitness map depends on 2 external parameters controlling for strength (α) and epistasis (β). If the phenotype-fitness map is explicit, the genotype-phenotype map is more pervasive. Mutations are seen has displacement of the phenotype in the multidimensional space. Beneficial mutations are moving the phenotype closer to 0, whereas deleterious mutations are moving the phenotype further away. In such original context, the distribution of mutational effects is not dependent on the current genotype, but this can be relaxed using a genotype-phenotype map.

In a protein context, the genotype-phenotype map can be defined by assigning to each of the 20 amino acid a vector in the multidimensional space. Since different sites of the protein do not have the same physico-chemical properties, we can define a specific genotype-phenotype map for each position of the sequence. Overall, the protein phenotype is computed as the sum of site-specific multidimensional vectors, obtained by accessing the amino acid present at each site of the protein. From a DNA sequence $\mathbb{S}^t$ after t substitutions, the protein's phenotype is given by:

$$\boldsymbol{P}\left(\mathbb{S}^t\right) = \sum_{\mathrm{z}=1}^{\mathrm{Z}} \boldsymbol{P}_{\mathrm{z}}\left(\mathbb{S}^t(\mathrm{z})\right), \tag{12}$$

where $\boldsymbol{P}_{\mathrm{z}}$ is the genotype-phenotype map at site z.

And the Wrightian fitness of $\mathbb{S}^t$ is :

$$W\left(\boldsymbol{P}\left(\mathbb{S}^t\right)\right) = e^{-\alpha\left|\boldsymbol{P}\left(\mathbb{S}^t\right)\right|^{\beta}}, \tag{13}$$

where strength ($\alpha > 0$) and epistasis (β) are parameters of the fitness function.

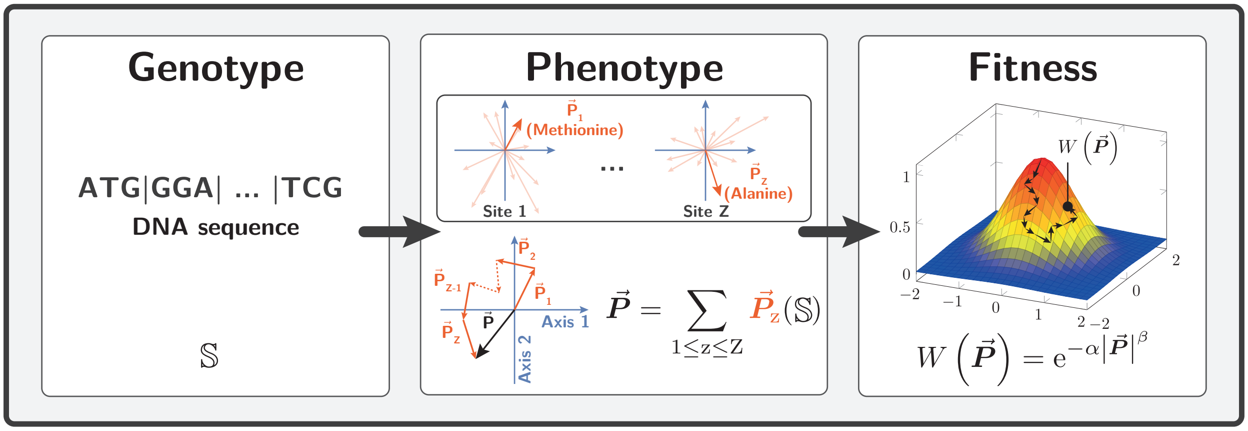

For each possible mutant (at time $t+1$ substitutions), we compute $\boldsymbol{P}\left(\mathbb{S}^{t+1}\right)$ from the updated sequence $\mathbb{S}^{t+1}$, and subsequently the selection coefficient of the mutant:

$$s\left(\mathbb{S}^{t}, \mathbb{S}^{t+1}\right) = \frac{W\left(\boldsymbol{P}\left(\mathbb{S}^{t+1}\right)\right) - W\left(\boldsymbol{P}\left(\mathbb{S}^{t}\right)\right)}{W\left(\boldsymbol{P}\left(\mathbb{S}^{t}\right)\right)}. \tag{14}$$

The next change in the protein coding DNA and the time to next the event is chosen using Gillespie's algorithm (Gillespie, 1977), according to the rates of substitution between codons:

$$Q_{i,j} = \mu_{i,j} \frac{4N_{\mathrm{e}} s\left(\mathbb{S}^{t}, \mathbb{S}^{t+1}\right)}{1 - e^{-4N_{\mathrm{e}} s\left(\mathbb{S}^{t}, \mathbb{S}^{t+1}\right)}}, \tag{15}$$

where $Q_{i,j} = \mu_{i,j}$ in the case of synonymous substitutions.

**Parameter**	Value
Mutation rate (at the root)	$u = 1.0 \times 10^{-8}$ per site per generation
Root age	150 Million years
Generation time (at the root)	$\tau = 10$ years
Exon size	300
Number of exons	16
Number of codon sites	$4,800$
Population size (at the root)	200
Complexity (number of dimensions)	3
Discretization of the geometric Brownian process	100 step per branch

Table 5: Parameters used for `SimuGeo`. The configuration files and scripts to produce simulations are available as `config.yaml` at https://github.com/ThibaultLatrille/MutationSelectionDrift.

The input nucleotide mutation matrix ($\boldsymbol{R}$) is a symmetric time-reversible matrix, which is automatically normalized by the simulator:

$$\boldsymbol{R} = \begin{array}{c} \\ A \\ C \\ G \\ T \end{array} \begin{array}{c} \begin{array}{cccc} A & C & G & T \end{array} \\ \begin{pmatrix} - & 1 & 1 & 1 \\ 1 & - & 1 & 1 \\ 1 & 1 & - & 1 \\ 1 & 1 & 1 & - \end{pmatrix} \end{array} \tag{16}$$

The input precision matrix ($\boldsymbol{\Omega} = \boldsymbol{\Sigma}^{-1}$) of the multivariate geometric Brownian is composed of three process, namely the effective population size (N_{e}), mutation rate per generation per site (u) and generation time (τ). The covariance matrix ($\boldsymbol{\Sigma}$) is computed from the input precision matrix ($\boldsymbol{\Omega}$) by the simulator, and represents the

covariances of traits along the tree (from root to leaves).

$$\mathbf{\Omega} = \begin{matrix} & N_{\mathrm{e}} & u & \tau \\ N_{\mathrm{e}} & \\ u & \\ \tau & \end{matrix} \begin{pmatrix} 0.5 & 0 & 0 \\ 0 & 2.0 & 0 \\ 0 & 0 & 1.0 \end{pmatrix} \tag{17}$$

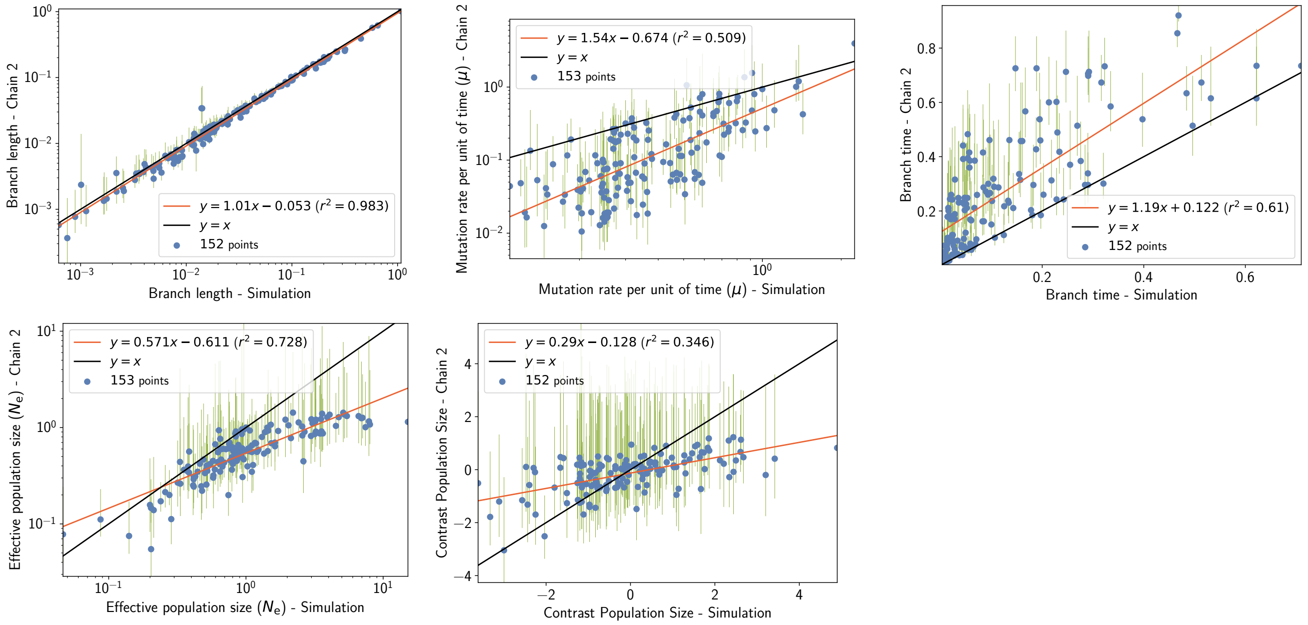

Figure 5: Inferred branch parameters under simulation accounting for site epistasis in geometric landscape, thus fluctuation of the selection coefficient along the phylogeny. Estimation is obtained with the mechanistic inference model developed in this paper of site-specific amino-acid fitness profiles and log-Brownian process for N_{e}, μ and life-history traits.

**Experiment**	$\langle \Omega \rangle$ **(branch** N_{e}**)**	$\langle \Omega \rangle$ **(constant** N_{e}**)**
`SimuGeo`, chain 1	2.27 ± 0.02	2.46 ± 0.02
`SimuGeo`, chain 2	2.23 ± 0.04	2.46 ± 0.02

Table 6: Estimated amino-acid entropy under simulation accounting for site epistasis (geometric landscape), thus fluctuation of the selection coefficient along the phylogeny. Estimation is obtained with the mechanistic inference model developed in this paper of site-specific amino-acid fitness profiles and log-Brownian process for N_{e}, μ and life-history traits (in the left column), or under the assumption of constant N_{e} (in the right column).

2.5 Protein folding probability (`SimuFold`)

We simulated substitutions in the protein phosphatase (Z = 300 codon sites) as in Goldstein and Pollock (2017). From a DNA sequence $\mathbb{S}^t$ after t substitutions, we compute the free energy of the folded state $G_{\mathrm{F}}\left(\mathbb{S}^t\right)$, using the 3-dimensional structure of the folded state and pair-wise contact energies between neighboring amino-acid residues:

$$G_{\mathrm{F}}\left(\mathbb{S}^t\right) = \sum_{\mathrm{z}=1}^{\mathrm{Z}} \sum_{r \in \mathcal{V}(\mathrm{z})} I\left(\mathbb{S}^t(\mathrm{z}), \mathbb{S}^t(r)\right), \tag{18}$$

where $I(a, b)$ is the pair-wise contact energies between amino acid a and b, using contact potentials estimated by Miyazawa and Jernigan (1985), and $\mathcal{V}(\mathrm{z})$ are the neighbor residues of site z (closer than 7Å) in the 3D structure.

The free energy of unfolded states $G_{\mathrm{U}}\left(\mathbb{S}^t\right)$ is approximated using 55 decoy 3D structures that supposedly represent a sample of possible unfolded states:

$$G_{\mathrm{U}}\left(\mathbb{S}^t\right) = \left\langle G\left(\mathbb{S}^t\right)\right\rangle - kT\ln(1.0\mathrm{E}^{160}) - \frac{2\left[\left\langle G\left(\mathbb{S}^t\right)^2\right\rangle - \left\langle G\left(\mathbb{S}^t\right)\right\rangle^2\right]}{kT} \tag{19}$$

where the average $\langle . \rangle$ runs other the 55 decoy 3D structures, and k is the Boltzmann constant and T the temperature in Kelvin.

From the energy of folded and unfolded states, we can compute the difference in free energy between the states:

$$\Delta G\left(\mathbb{S}^t\right) = G_{\mathrm{F}}\left(\mathbb{S}^t\right) - G_{\mathrm{U}}\left(\mathbb{S}^t\right) \tag{20}$$

Wrightian fitness is defined as the probability of our protein to be in the folded state:

$$W\left(\Delta G\left(\mathbb{S}^t\right)\right) = \mathbb{P}_{\mathrm{F}}\left(\mathbb{S}^t\right) = \frac{e^{-\beta G_{\mathrm{F}}\left(\mathbb{S}^t\right)}}{e^{-\beta G_{\mathrm{F}}\left(\mathbb{S}^t\right)} + e^{-\beta G_{\mathrm{U}}\left(\mathbb{S}^t\right)}} = \frac{1}{1+e^{\beta \Delta G\left(\mathbb{S}^t\right)}}, \tag{21}$$

where β is the inverse of the temperature ($\beta = 1/kT$).

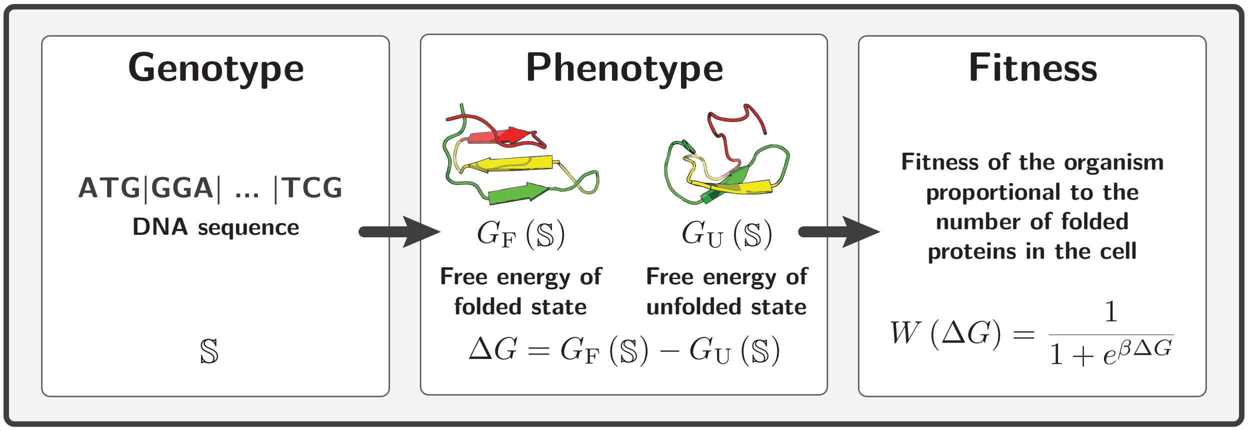

For each possible mutant (at time $t+1$ substitutions), we compute ΔG^{t+1} from the updated sequence $\mathbb{S}^{t+1}$, and subsequently the selection coefficient of the mutant:

$$s\left(\mathbb{S}^t, \mathbb{S}^{t+1}\right) = \frac{W\left(\Delta G\left(\mathbb{S}^{t+1}\right)\right) - W\left(\Delta G\left(\mathbb{S}^t\right)\right)}{W\left(\Delta G\left(\mathbb{S}^t\right)\right)}. \tag{22}$$

The next change in the protein coding DNA and the time to next the event is chosen using Gillespie's algorithm (Gillespie, 1977), according to the rates of substitution between codons:

$$Q_{i,j} = \mu_{i,j}\frac{4N_{\mathrm{e}}s\left(\mathbb{S}^t, \mathbb{S}^{t+1}\right)}{1-\mathrm{e}^{-4N_{\mathrm{e}}s\left(\mathbb{S}^t, \mathbb{S}^{t+1}\right)}}, \tag{23}$$

where $Q_{i,j} = \mu_{i,j}$ in the case of synonymous substitutions.

**Parameter**	Value
Mutation rate (at the root)	$u = 1.0 \times 10^{-8}$ per site per generation
Root age	150 Million years
Generation time (at the root)	$\tau = 10$ years
Protein	Protein phosphatase (Goldstein and Pollock, 2017)
Exon size	300
Number of exons	16
Number of codon sites	4,800
Population size (at the root)	100,000
Discretization of the geometric Brownian process	100 step per branch

Table 7: Parameters used for `SimuGeo`. The configuration files and scripts to produce simulations are available as `config.yaml` at `https://github.com/ThibaultLatrille/MutationSelectionDrift`.

The input nucleotide mutation matrix ($\boldsymbol{R}$) is a symmetric time-reversible matrix, which is automatically normalized by the simulator:

$$\boldsymbol{R} = \begin{matrix} & \begin{matrix} A & C & G & T \end{matrix} \\ \begin{matrix} A \\ C \\ G \\ T \end{matrix} & \begin{pmatrix} - & 1 & 1 & 1 \\ 1 & - & 1 & 1 \\ 1 & 1 & - & 1 \\ 1 & 1 & 1 & - \end{pmatrix} \end{matrix} \tag{24}$$

The input precision matrix ($\boldsymbol{\Omega} = \boldsymbol{\Sigma}^{-1}$) of the multivariate geometric Brownian is composed of three process, namely the effective population size (N_{e}), mutation rate per generation per site (u) and generation time (τ). The covariance matrix ($\boldsymbol{\Sigma}$) is computed from the input precision matrix ($\boldsymbol{\Omega}$) by the simulator, and represents the covariances of traits along the tree (from root to leaves).

$$\boldsymbol{\Omega} = \begin{matrix} & \begin{matrix} N_{\mathrm{e}} & u & \tau \end{matrix} \\ \begin{matrix} N_{\mathrm{e}} \\ u \\ \tau \end{matrix} & \begin{pmatrix} 0.5 & 0 & 0 \\ 0 & 2.0 & 0 \\ 0 & 0 & 1.0 \end{pmatrix} \end{matrix} \tag{25}$$

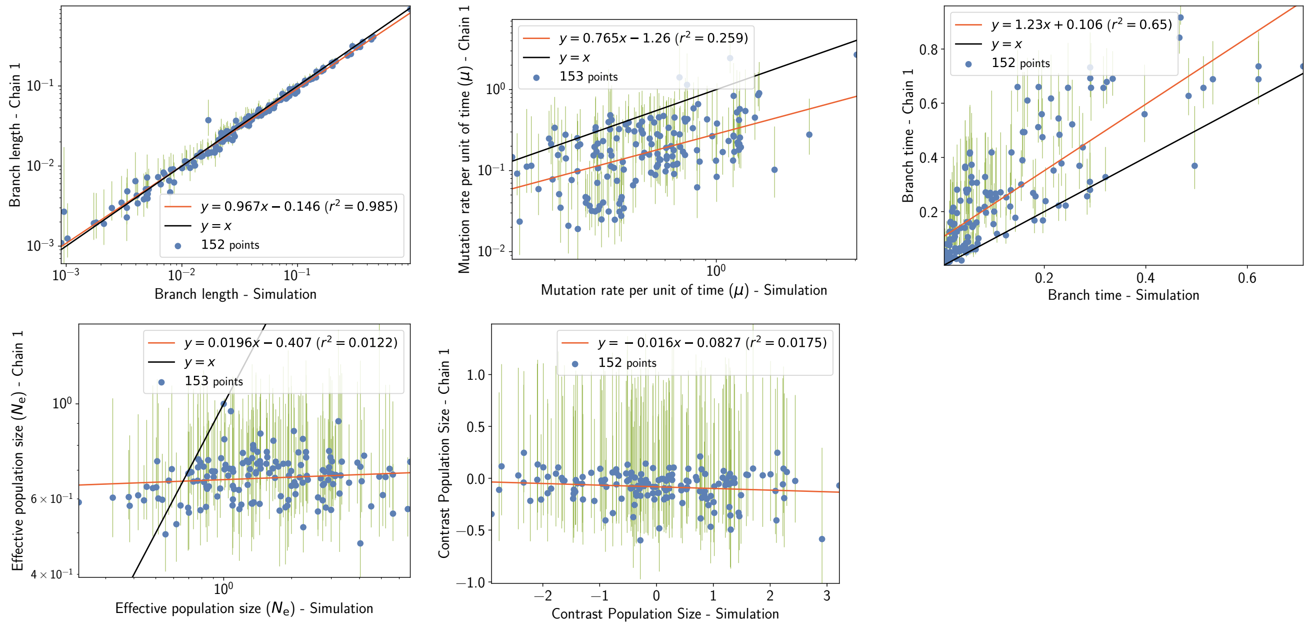

Figure 6: Inferred branch parameters under simulation accounting for site epistasis (folding stability model), thus fluctuation of the selection coefficient along the phylogeny. Estimation is obtained with the mechanistic inference model developed in this paper of site-specific amino-acid fitness profiles and log-Brownian process for N_{e}, μ and life-history traits.

**Experiment**	$\langle\Omega\rangle$ **(branch** N_{e}**)**	$\langle\Omega\rangle$ **(constant** N_{e}**)**
`SimuFold`, chain 1	1.31 ± 0.05	1.61 ± 0.03
`SimuFold`, chain 2	1.30 ± 0.04	1.60 ± 0.03

Table 8: Estimated amino-acid entropy under simulation accounting for site epistasis (folding stability model), thus fluctuation of the selection coefficient along the phylogeny. Obtained with the mechanistic inference model developed in this paper of site-specific amino-acid fitness profiles and log-Brownian process for N_{e}, μ and life-history traits (in the left column), or under the assumption of constant N_{e} (in the right column).

2.6 Identifiability of N_e and μ

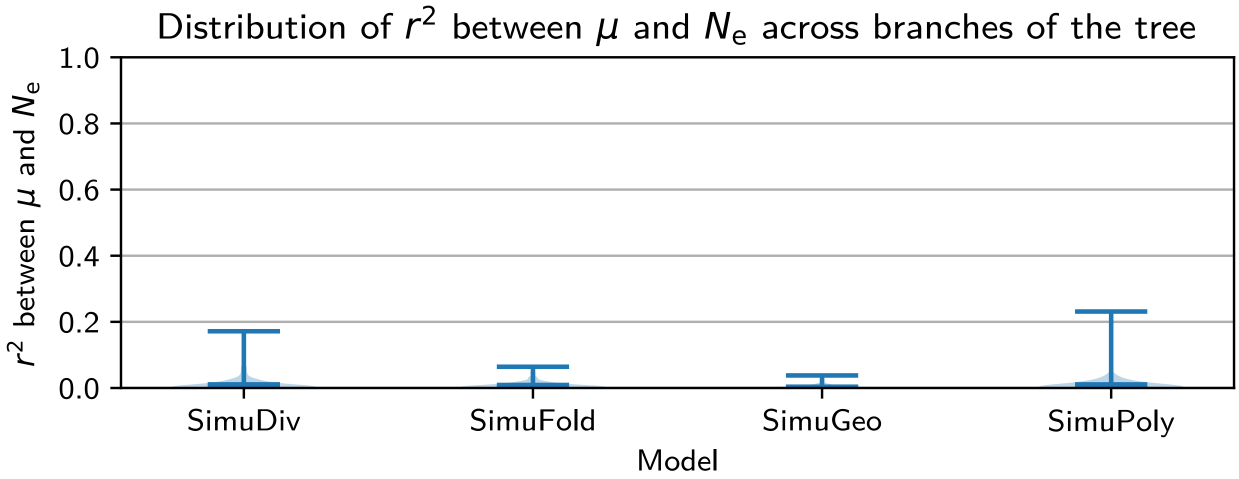

Figure 7: Some parameters might be strongly correlated or only weakly identifiable, which may make it hard to fit the model to realistically sized datasets. To test whether N_e and μ are identifiable, for each branch of the tree we draw a 2-D scatter plot for N_e and μ, where each point is a step in the MCMC procedure. We subsequently fit a linear regression and compute the coefficient of determination (r^2) for each branch of the tree. For a given MCMC, the distribution of r^2 across all branches is then represented as a violin plot. The distribution of r^2 is relatively low, below 0.01 on average, providing confidence that N_e and μ are indeed identifiable.

3 Empirical data in mammals

Only highly conserved coding sequences are kept for the analysis, representing 226 genes with $\leq 1\%$ of gaps in the alignment. We verified that they do not display a signature of positive selection using a site codon model. The results of site codon models are available in a tabulated file at `https://github.com/ThibaultLatrille/MutationSelectionDrift/blob/master/DataEmpirical/OrthoMam/orthomam.tsv` The list of genes included in the analysis is (also available at `https://github.com/ThibaultLatrille/MutationSelectionDrift/blob/master/DataEmpirical/OrthoMam/cds.highcoverage.list`):
ENSG00000264364 ENSG00000171617 ENSG00000175155 ENSG00000101608 ENSG00000119431 ENSG00000165527 ENSG00000177125 ENSG00000170745 ENSG00000013392 ENSG00000165474 ENSG00000181218 ENSG00000186130 ENSG00000178235 ENSG00000174482 ENSG00000184076 ENSG00000128710 ENSG00000168539 ENSG00000162630 ENSG00000123595 ENSG00000157551 ENSG00000180785 ENSG00000146006 ENSG00000185985 ENSG00000166170 ENSG00000179796 ENSG00000205279 ENSG00000186468 ENSG00000196542 ENSG00000184209 ENSG00000163866 ENSG00000166923 ENSG00000214194 ENSG00000203814 ENSG00000119401 ENSG00000166292 ENSG00000177868 ENSG00000181773 ENSG00000171243 ENSG00000133019 ENSG00000189143 ENSG00000120963 ENSG00000135211 ENSG00000184602 ENSG00000156284 ENSG00000088986 ENSG00000181817 ENSG00000187713 ENSG00000066322 ENSG00000232112 ENSG00000113658 ENSG00000213593 ENSG00000196570 ENSG00000254598 ENSG00000197403 ENSG00000111262 ENSG00000149591 ENSG00000181467 ENSG00000178585 ENSG00000184697 ENSG00000169976 ENSG00000258818 ENSG00000016082 ENSG00000084774 ENSG00000177938 ENSG00000123395 ENSG00000167414 ENSG00000188997 ENSG00000127774 ENSG00000144230 ENSG00000180332 ENSG00000170989 ENSG00000198033 ENSG00000198081 ENSG00000140006 ENSG00000125995 ENSG00000153879 ENSG00000157005 ENSG00000185070 ENSG00000176422 ENSG00000168496 ENSG00000147588 ENSG00000185909 ENSG00000134809 ENSG00000172209 ENSG00000198523 ENSG00000172057 ENSG00000171786 ENSG00000171488 ENSG00000179542 ENSG00000278637 ENSG00000253148 ENSG00000197063 ENSG00000177954 ENSG00000185361 ENSG00000175928 ENSG00000187918 ENSG00000169717 ENSG00000163347 ENSG00000171155 ENSG00000131808 ENSG00000143878 ENSG00000125848 ENSG00000164751 ENSG00000188133 ENSG00000221813 ENSG00000168421 ENSG00000186469 ENSG00000165023 ENSG00000262179 ENSG00000170903 ENSG00000183475 ENSG00000180772 ENSG00000162188 ENSG00000183671

ENSG00000253719 ENSG00000176490 ENSG00000276410 ENSG00000156253 ENSG00000182117 ENSG00000180660 ENSG00000164600 ENSG00000102981 ENSG00000117318 ENSG00000170915 ENSG00000221886 ENSG00000159713 ENSG00000169155 ENSG00000159197 ENSG00000123416 ENSG00000185972 ENSG00000175793 ENSG00000233932 ENSG00000177971 ENSG00000113013 ENSG00000112218 ENSG00000109606 ENSG00000148948 ENSG00000143105 ENSG00000123700 ENSG00000124733 ENSG00000139826 ENSG00000164978 ENSG00000175197 ENSG00000140285 ENSG00000188636 ENSG00000168301 ENSG00000126778 ENSG00000159251 ENSG00000176907 ENSG00000178750 ENSG00000187513 ENSG00000177981 ENSG00000170340 ENSG00000196154 ENSG00000123307 ENSG00000177105 ENSG00000155868 ENSG00000165496 ENSG00000147145 ENSG00000269743 ENSG00000123080 ENSG00000034510 ENSG00000213937 ENSG00000178764 ENSG00000178033 ENSG00000188725 ENSG00000221933 ENSG00000188042 ENSG00000091010 ENSG00000178093 ENSG00000163154 ENSG00000127588 ENSG00000172336 ENSG00000256235 ENSG00000168582 ENSG00000188486 ENSG00000119938 ENSG00000175264 ENSG00000157224 ENSG00000111269 ENSG00000166192 ENSG00000180440 ENSG00000174946 ENSG00000144476 ENSG00000204390 ENSG00000166603 ENSG00000128340 ENSG00000158710 ENSG00000121742 ENSG00000162772 ENSG00000162734 ENSG00000235711 ENSG00000187678 ENSG00000164708 ENSG00000176533 ENSG00000124479 ENSG00000123570 ENSG00000167635 ENSG00000156127 ENSG00000186767 ENSG00000168795 ENSG00000070831 ENSG00000135116 ENSG00000152954 ENSG00000144362 ENSG00000173915 ENSG00000168917 ENSG00000156486 ENSG00000143546 ENSG00000165152 ENSG00000176087 ENSG00000214827 ENSG00000157150 ENSG00000172318 ENSG00000159248 ENSG00000170961 ENSG00000164484 ENSG00000197409 ENSG00000155366 ENSG00000146386 ENSG00000175567 ENSG00000181690 ENSG00000182544 ENSG00000181541 ENSG00000160307 ENSG00000132581

4 independent inferences were performed on a randomly chosen set of 18 coding sequences (CDS) out of the 226.

The replicate 1 is composed of :
ENSG00000066322 ENSG00000183671 ENSG00000156127 ENSG00000123307 ENSG00000140285 ENSG00000221813 ENSG00000157005 ENSG00000213593 ENSG00000128710 ENSG00000180332 ENSG00000126778 ENSG00000175567 ENSG00000034510 ENSG00000176907 ENSG00000162188 ENSG00000184076 ENSG00000187678 ENSG00000159713

The replicate 2 is composed of :
ENSG00000166603 ENSG00000147588 ENSG00000221933 ENSG00000167635 ENSG00000170340 ENSG00000157150 ENSG00000135211 ENSG00000155868 ENSG00000196154 ENSG00000204390 ENSG00000184602 ENSG00000258818 ENSG00000187713 ENSG00000127774 ENSG00000169976 ENSG00000185361 ENSG00000181218 ENSG00000168582

The replicate 3 is composed of :
ENSG00000198081 ENSG00000126778 ENSG00000164751 ENSG00000221813 ENSG00000163347 ENSG00000125995 ENSG00000167635 ENSG00000155366 ENSG00000174482 ENSG00000159197 ENSG00000178750 ENSG00000102981 ENSG00000254598 ENSG00000128340 ENSG00000171786 ENSG00000180440 ENSG00000262179 ENSG00000157005

The replicate 4 is composed of :
ENSG00000177868 ENSG00000123700 ENSG00000157150 ENSG00000183671 ENSG00000113013 ENSG00000175793 ENSG00000128340 ENSG00000121742 ENSG00000167414 ENSG00000169976 ENSG00000160307 ENSG00000214194 ENSG00000135116 ENSG00000170903 ENSG00000156127 ENSG00000157551 ENSG00000187678 ENSG00000177105

3.1 Chain convergence

Obtained with the mechanistic inference model developed in this paper of site-specific amino-acid fitness profiles and log-Brownian process for N_{e}, μ and life-history traits.

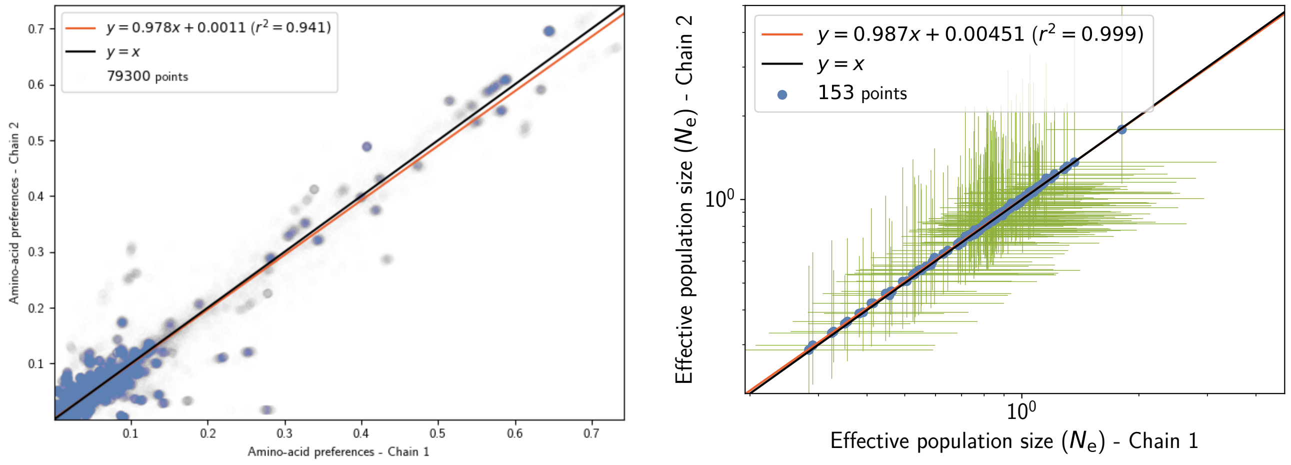

Figure 8: Chain convergence of site amino-acid preferences (left panel) and branch N_{e} (right panel).

3.2 Traits estimation & correlation (replicate 1, chain 1)

Obtained with the mechanistic inference model developed in this paper of site-specific amino-acid fitness profiles and log-Brownian process for N_{e}, μ and life-history traits.

Covariance (Σ)	N_{e}	μ	Maximum longevity	Adult weight	Female maturity
N_{e}	0.281**	0.324**	−0.268**	−1.29**	−0.308**
μ	-	1.93**	−1.12**	−5.19**	−1.43**
Maximum longevity	-	-	0.934**	3.58**	1.01**
Adult weight	-	-	-	19.9**	4.48**
Female maturity	-	-	-	-	1.53**

Table 9: Covariance coefficient between effective population size (N_{e}), mutation rate per site per unit of time (μ), and life-history traits (maximum longevity, adult weight and female maturity) were computed in placental mammals. Asterisks indicate strength of support ($^*pp > 0.95$, $^{**}pp > 0.975$).

Partial coefficient	N_{e}	μ	Maximum longevity	Adult weight	Female maturity
N_{e}	-	−0.146	−0.177	−0.265*	−0.0223
μ	-	-	−0.283*	−0.396**	−0.327**
Maximum longevity	-	-	-	0.236*	0.383**
Adult weight	-	-	-	-	0.179
Female maturity	-	-	-	-	-

Table 10: Partial correlation coefficient between effective population size (N_{e}), mutation rate per site per unit of time (μ), and life-history traits (maximum longevity, adult weight and female maturity) were computed in placental mammals. Asterisks indicate strength of support ($^*pp > 0.95$, $^{**}pp > 0.975$).

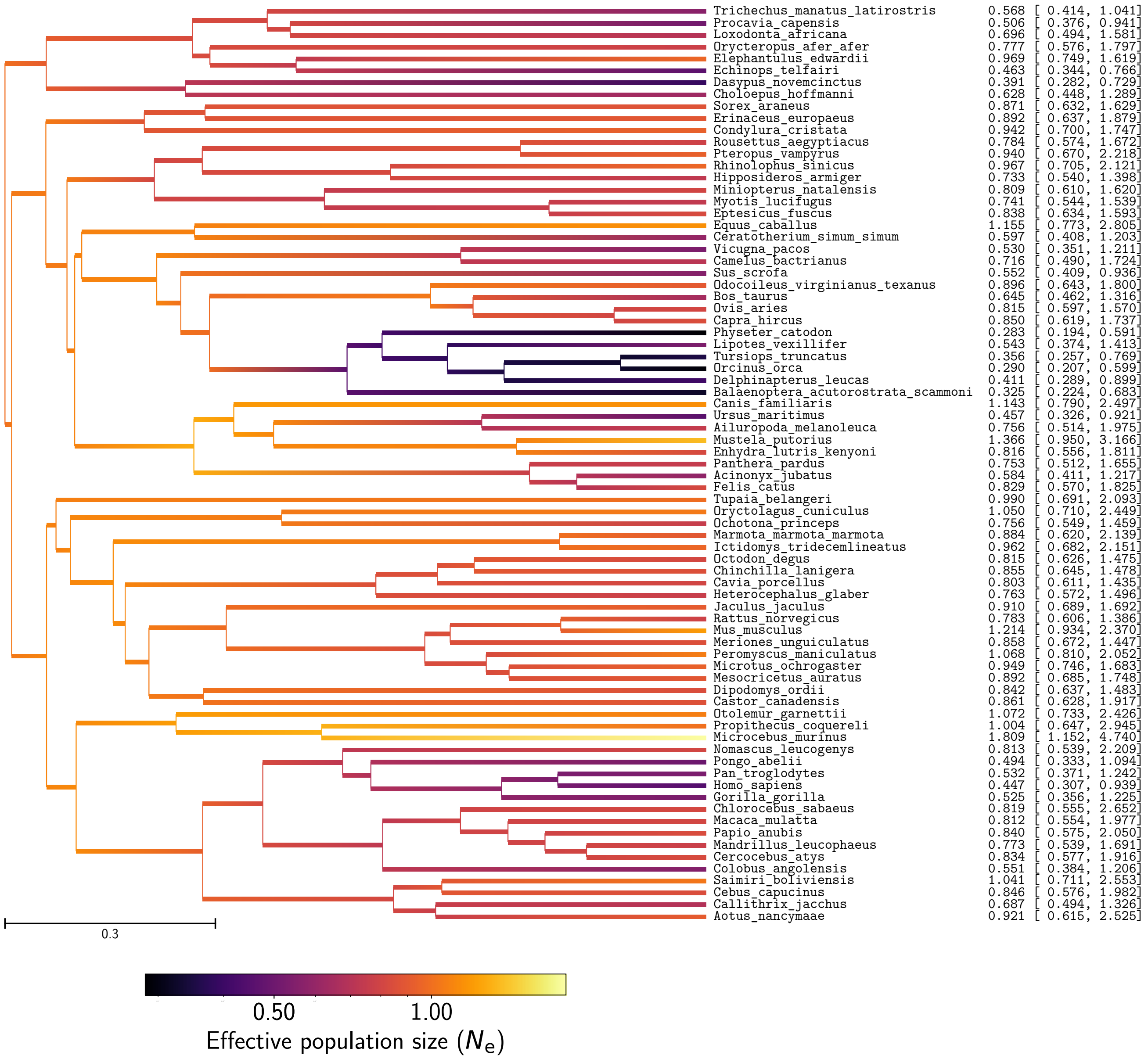

Figure 9: Effective population size (N_e) estimation in mammals

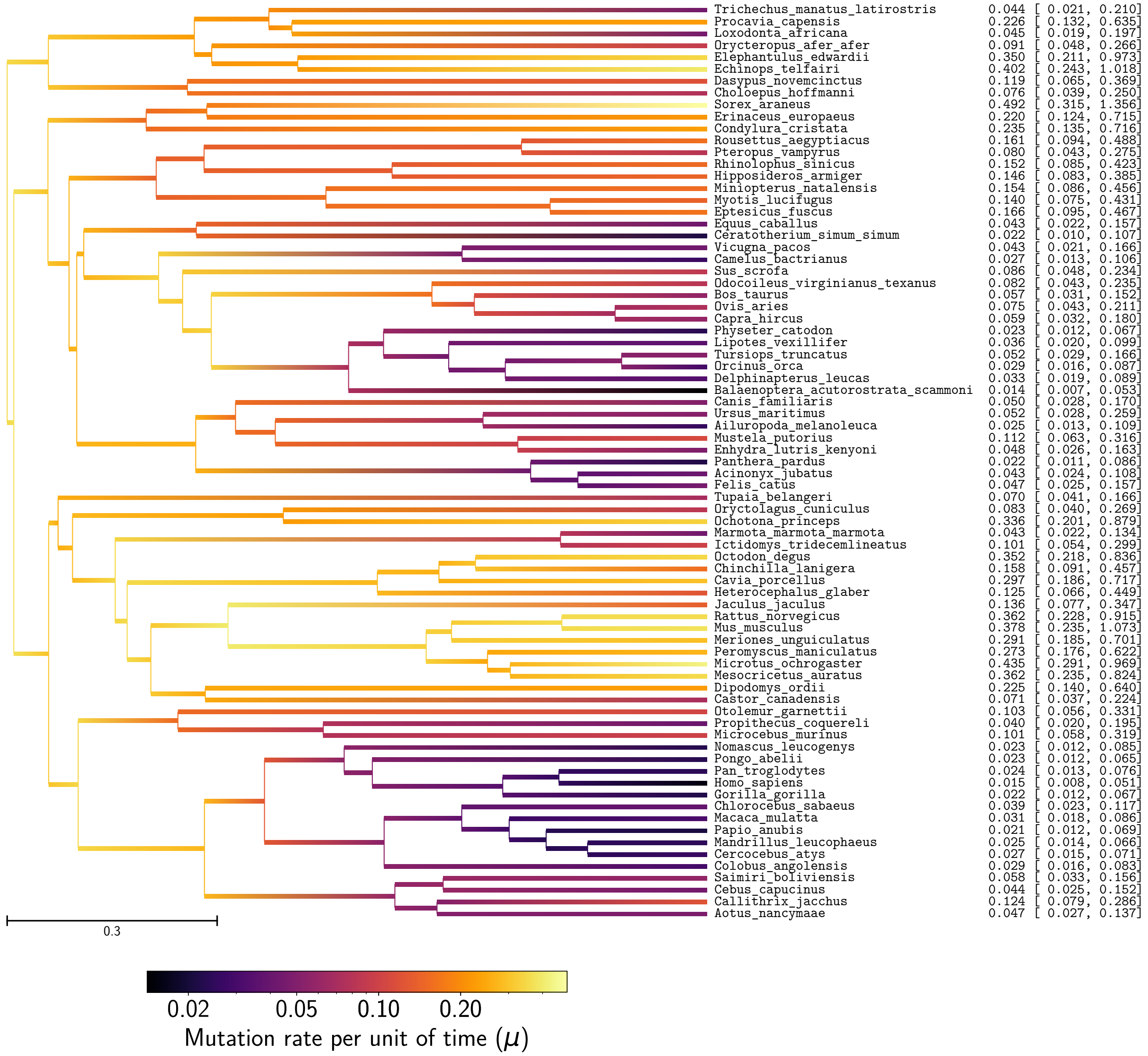

Figure 10: Mutation rate (μ) estimation in mammals

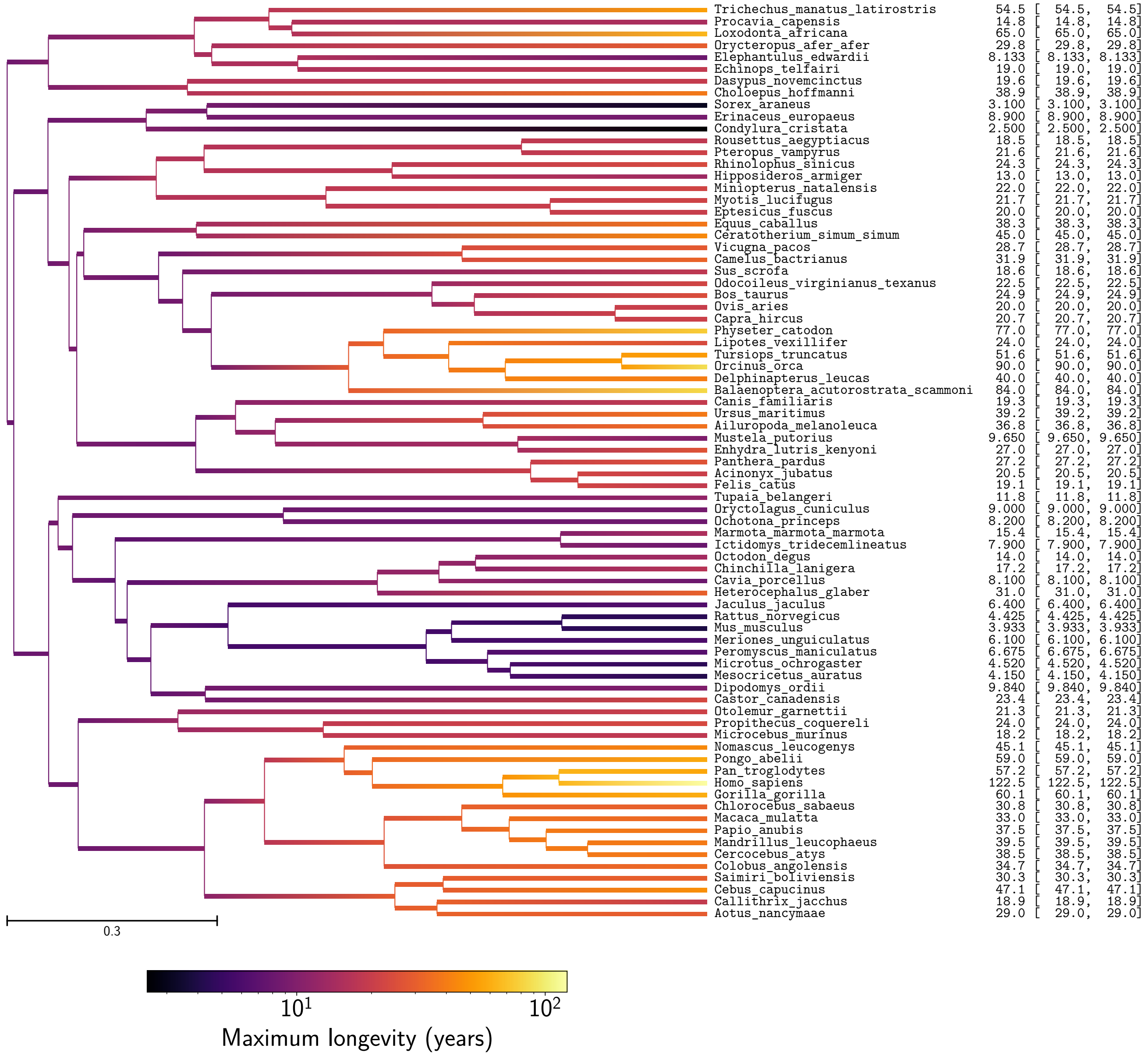

Figure 11: Maximum longevity estimation in mammals

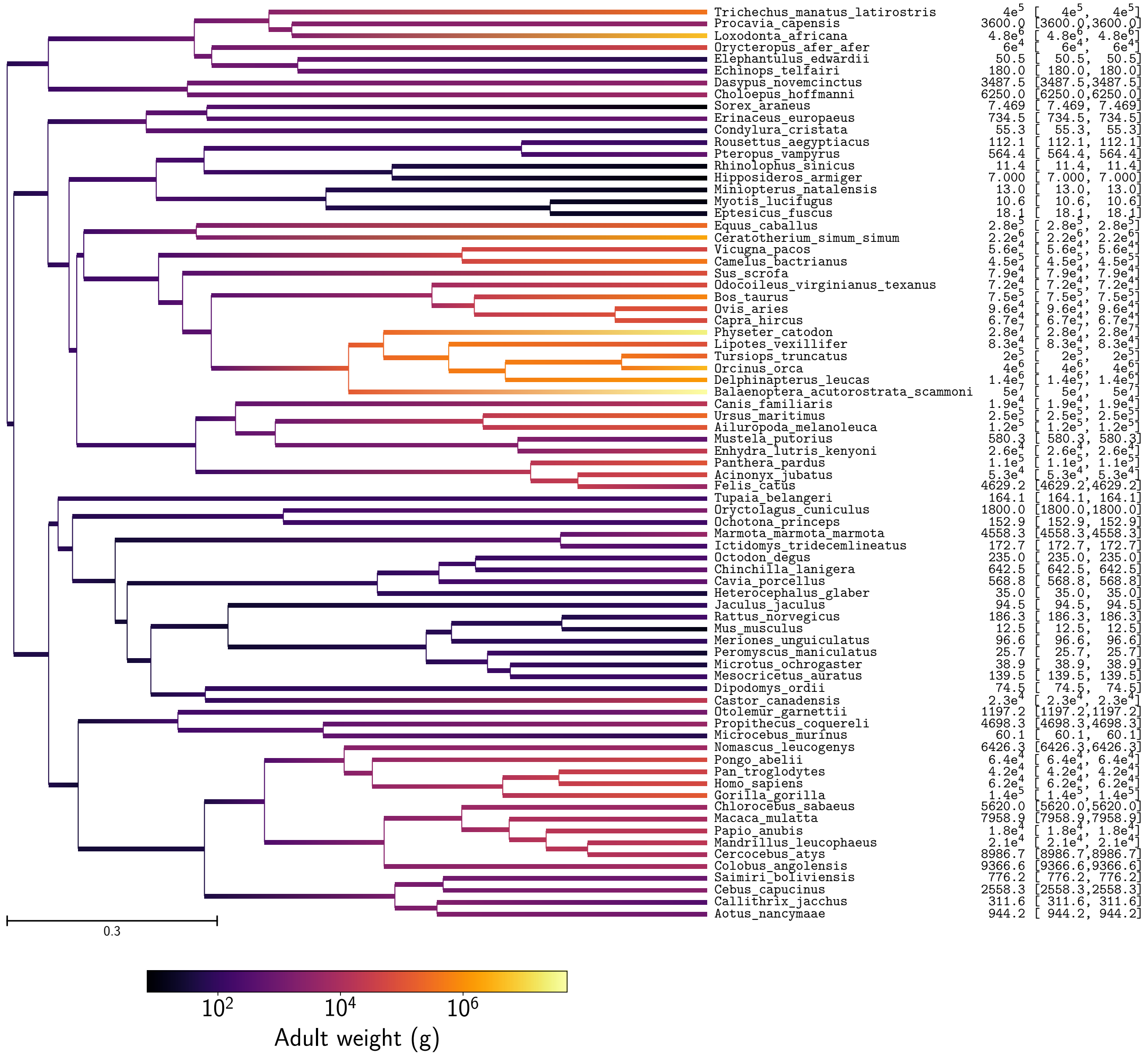

Figure 12: Adult weight estimation in mammals

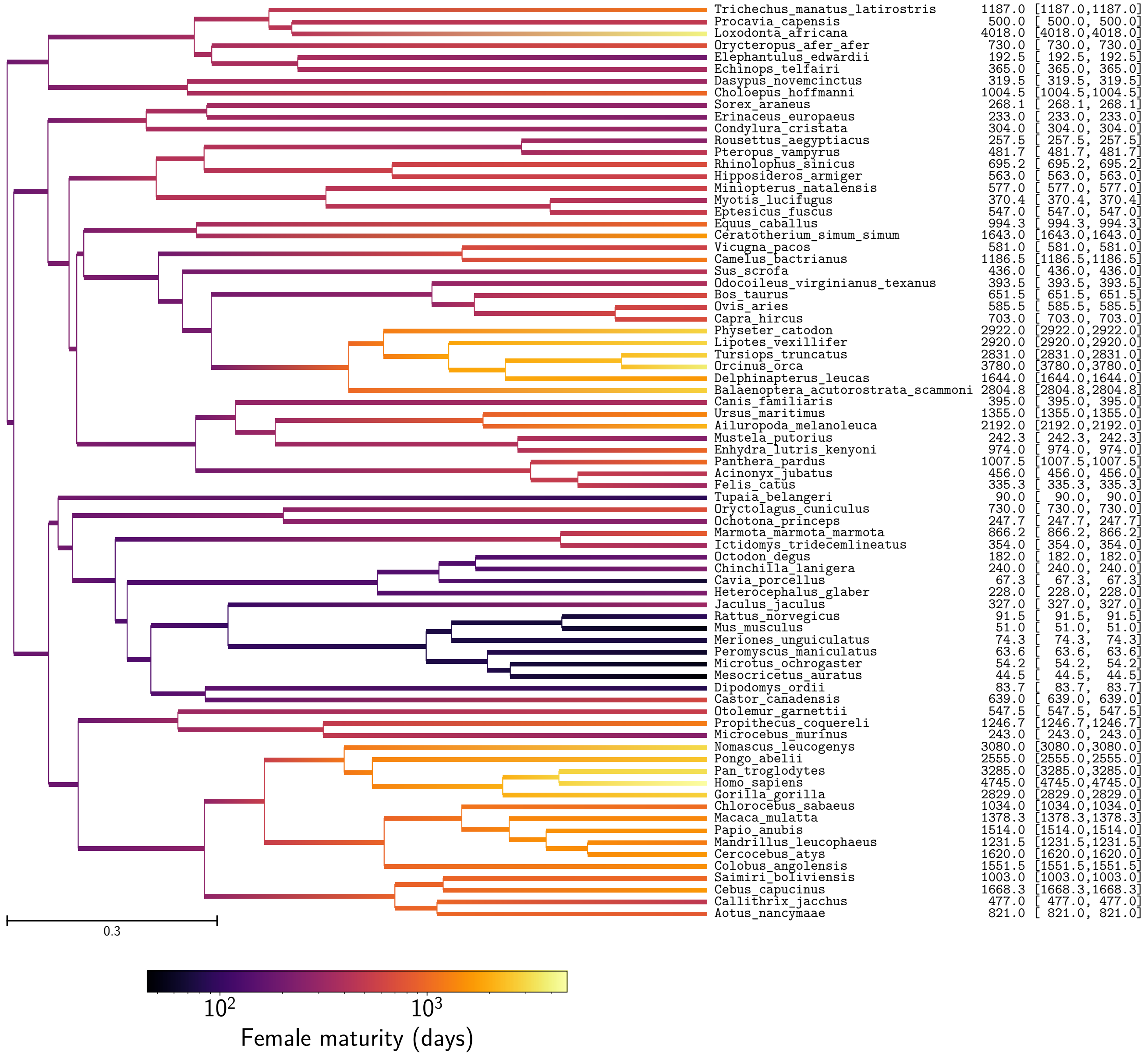

Figure 13: Female maturity estimation in mammals

3.3 Repeatability of experiments

4 independent inferences were performed on a randomly chosen set of 18 coding sequences (CDS) out of 226. Obtained with the mechanistic inference model developed in this paper of site-specific amino-acid fitness profiles and log-Brownian process for N_e, μ and life-history traits. Each plot is a correlation between a pair of experiments for a given parameter. For each node (or branch) of the tree, the mean posterior of the parameter over the MCMC (after burn-in) is represented in blue dots, green solid lines are the 90% confidence interval of the MCMC. Solid red line is the regression line between replicates.

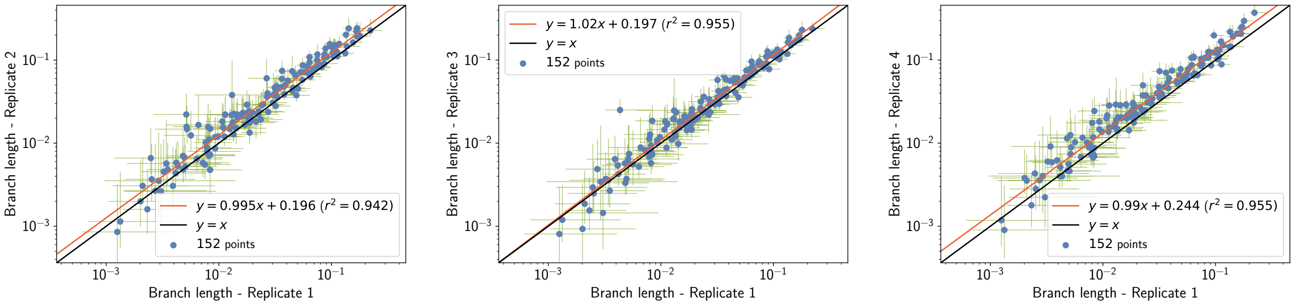

Figure 14: Repeatability of branch length (l) estimation in mammals

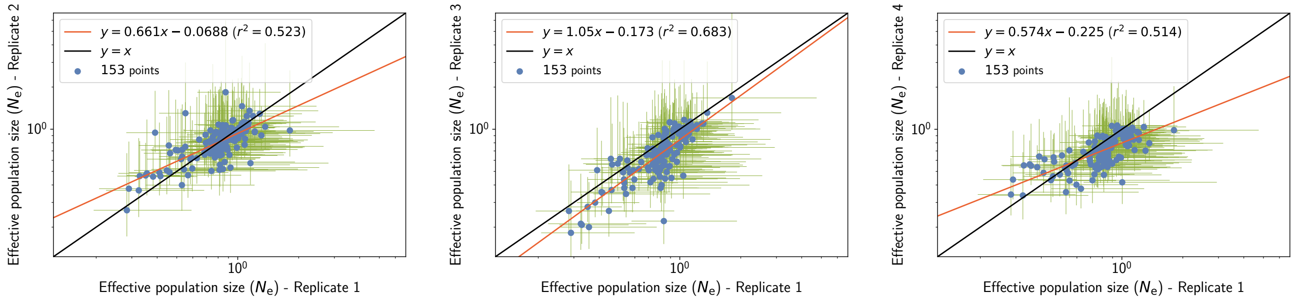

Figure 15: Repeatability of effective population size (N_{e}) estimation in mammals

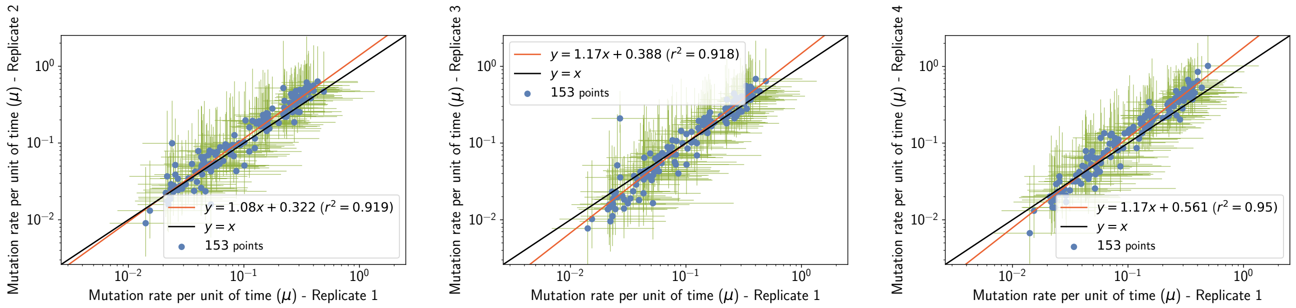

Figure 16: Repeatability of mutation rate (μ) estimation in mammals

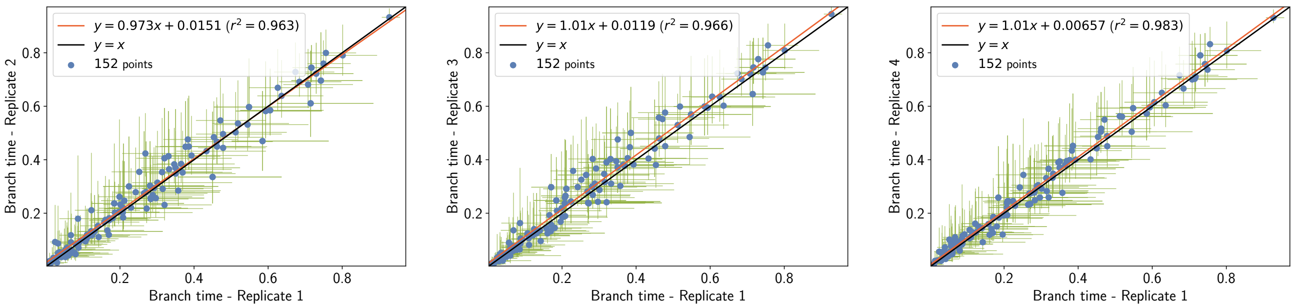

Figure 17: Repeatability of branch time (ΔT) estimation in mammals

Rep. 1	Rep. 2	Rep. 3	Rep. 4	Taxon
0.568	0.469	0.489	0.548	*Trichechus manatus latirostris*
0.506	0.706	0.615	0.65	*Procavia capensis*
0.696	0.799	0.532	0.595	*Loxodonta africana*
0.777	0.812	0.651	0.717	*Orycteropus afer afer*
0.969	0.904	0.68	0.949	*Elephantulus edwardii*
0.463	0.673	0.56	0.586	*Echinops telfairi*
0.391	0.945	0.51	0.639	*Dasypus novemcinctus*
0.628	0.621	0.52	0.376	*Choloepus hoffmanni*
0.871	1.84	0.745	0.819	*Sorex araneus*
0.892	0.833	1.12	1.06	*Erinaceus europaeus*
0.942	1.15	1.1	0.916	*Condylura cristata*
0.784	0.679	0.488	0.535	*Rousettus aegyptiacus*
0.94	0.838	0.662	0.604	*Pteropus vampyrus*
0.967	0.823	0.586	0.636	*Rhinolophus sinicus*
0.733	0.98	0.876	0.746	*Hipposideros armiger*
0.809	0.934	0.742	0.738	*Miniopterus natalensis*
0.741	0.504	0.442	0.53	*Myotis lucifugus*
0.838	0.849	0.588	0.753	*Eptesicus fuscus*
1.16	0.573	0.846	0.711	*Equus caballus*
0.597	0.524	0.438	0.402	*Ceratotherium simum simum*
0.53	0.399	0.438	0.356	*Vicugna pacos*
0.716	0.68	0.418	0.432	*Camelus bactrianus*
0.552	1.3	0.531	0.43	*Sus scrofa*
0.896	0.861	0.761	0.568	*Odocoileus virginianus texanus*
0.645	0.844	0.583	0.69	*Bos taurus*
0.815	0.649	0.747	0.473	*Ovis aries*
0.85	0.723	0.742	0.538	*Capra hircus*
0.283	0.264	0.261	0.342	*Physeter catodon*
0.543	0.517	0.345	0.486	*Lipotes vexillifer*
0.356	0.484	0.2	0.549	*Tursiops truncatus*
0.29	0.376	0.182	0.437	*Orcinus orca*
0.411	0.491	0.356	0.488	*Delphinapterus leucas*
0.325	0.366	0.211	0.337	*Balaenoptera acutorostrata scammoni*
1.14	1.35	1	0.842	*Canis familiaris*
0.457	0.766	0.615	0.461	*Ursus maritimus*
0.756	0.716	0.569	0.53	*Ailuropoda melanoleuca*
1.37	1.04	1.31	0.795	*Mustela putorius*
0.816	0.557	0.7	0.657	*Enhydra lutris kenyoni*
0.753	0.881	0.683	0.556	*Panthera pardus*
0.584	0.61	0.381	0.561	*Acinonyx jubatus*
0.829	0.761	0.655	0.602	*Felis catus*
0.99	0.738	1.04	0.627	*Tupaia belangeri*
1.05	1.46	1.17	0.897	*Oryctolagus cuniculus*
0.756	0.751	0.514	0.902	*Ochotona princeps*
0.884	0.746	0.541	0.862	*Marmota marmota marmota*
0.962	0.933	0.773	0.977	*Ictidomys tridecemlineatus*
0.815	0.733	0.688	0.874	*Octodon degus*
0.855	0.979	0.691	0.645	*Chinchilla lanigera*
0.803	1.08	0.684	0.898	*Cavia porcellus*
0.763	0.76	0.702	0.518	*Heterocephalus glaber*
0.91	0.655	0.449	0.865	*Jaculus jaculus*
0.783	0.956	0.91	0.883	*Rattus norvegicus*
1.21	0.963	1.01	0.839	*Mus musculus*
0.858	0.856	0.818	0.828	*Meriones unguiculatus*
1.07	1.11	0.877	0.757	*Peromyscus maniculatus*
0.949	1.16	1.01	1.06	*Microtus ochrogaster*
0.892	0.949	1.12	0.788	*Mesocricetus auratus*
0.842	1.07	1.01	0.695	*Dipodomys ordii*
0.861	0.583	0.494	0.575	*Castor canadensis*
1.07	1.13	0.812	0.821	*Otolemur garnettii*
1	0.945	0.741	0.418	*Propithecus coquereli*
1.81	0.98	1.67	0.985	*Microcebus murinus*
0.813	0.512	0.399	0.582	*Nomascus leucogenys*
0.494	0.71	0.568	0.475	*Pongo abelii*
0.532	0.713	0.381	0.513	*Pan troglodytes*
0.447	0.508	0.261	0.433	*Homo sapiens*
0.525	0.611	0.493	0.528	*Gorilla gorilla*
0.819	0.754	0.782	0.71	*Chlorocebus sabaeus*
0.812	0.816	0.538	0.679	*Macaca mulatta*
0.84	0.8	0.555	0.676	*Papio anubis*
0.773	0.813	0.501	0.628	*Mandrillus leucophaeus*
0.834	0.823	0.221	0.631	*Cercocebus atys*
0.551	0.749	0.599	0.706	*Colobus angolensis*
1.04	0.93	0.466	0.859	*Saimiri boliviensis*
0.846	0.519	0.444	0.667	*Cebus capucinus*
0.687	0.658	0.659	0.805	*Callithrix jacchus*
0.921	0.532	0.614	0.794	*Aotus nancymaae*
**6.38**	**6.96**	**9.19**	**3.16**	**Maximum range**

Table 11: Repeatability of effective population size (N_e) estimation in mammals, for the extant taxa.

Rep. 1	Rep. 2	Rep. 3	Rep. 4	Taxon
0.0436	0.039	0.0645	0.0422	*Trichechus manatus latirostris*
0.226	0.281	0.369	0.229	*Procavia capensis*
0.0455	0.0656	0.0435	0.0342	*Loxodonta africana*
0.0909	0.0995	0.144	0.0862	*Orycteropus afer afer*
0.35	0.614	0.434	0.457	*Elephantulus edwardii*
0.402	0.478	0.684	0.848	*Echinops telfairi*
0.119	0.194	0.104	0.0861	*Dasypus novemcinctus*
0.0764	0.0713	0.109	0.0915	*Choloepus hoffmanni*
0.492	0.468	0.64	1.01	*Sorex araneus*
0.22	0.521	0.281	0.317	*Erinaceus europaeus*
0.235	0.228	0.282	0.352	*Condylura cristata*
0.161	0.173	0.186	0.266	*Rousettus aegyptiacus*
0.08	0.0942	0.0565	0.103	*Pteropus vampyrus*
0.152	0.222	0.157	0.158	*Rhinolophus sinicus*
0.146	0.232	0.208	0.164	*Hipposideros armiger*
0.154	0.166	0.135	0.23	*Miniopterus natalensis*
0.14	0.164	0.174	0.158	*Myotis lucifugus*
0.166	0.154	0.166	0.217	*Eptesicus fuscus*
0.0428	0.0657	0.0345	0.0404	*Equus caballus*
0.0217	0.0368	0.0158	0.017	*Ceratotherium simum simum*
0.0431	0.0512	0.0335	0.0788	*Vicugna pacos*
0.027	0.0244	0.0357	0.0244	*Camelus bactrianus*
0.0859	0.0431	0.0402	0.0497	*Sus scrofa*
0.0818	0.0677	0.0525	0.0805	*Odocoileus virginianus texanus*
0.057	0.0696	0.0575	0.0424	*Bos taurus*
0.0753	0.0883	0.0886	0.101	*Ovis aries*
0.0586	0.0764	0.0653	0.0641	*Capra hircus*
0.0234	0.0291	0.0161	0.0181	*Physeter catodon*
0.0355	0.0534	0.0373	0.038	*Lipotes vexillifer*
0.0524	0.0474	0.0481	0.0393	*Tursiops truncatus*
0.0292	0.0254	0.0184	0.0171	*Orcinus orca*
0.0331	0.0347	0.019	0.0318	*Delphinapterus leucas*
0.0141	0.00903	0.00772	0.0067	*Balaenoptera acutorostrata scammoni*
0.0505	0.0795	0.0329	0.065	*Canis familiaris*
0.0518	0.0158	0.0165	0.0256	*Ursus maritimus*
0.0255	0.0517	0.0513	0.0368	*Ailuropoda melanoleuca*
0.112	0.0774	0.101	0.163	*Mustela putorius*
0.0479	0.0513	0.0456	0.0528	*Enhydra lutris kenyoni*
0.0218	0.0175	0.0144	0.0194	*Panthera pardus*
0.0426	0.0261	0.039	0.0486	*Acinonyx jubatus*
0.0465	0.0235	0.038	0.038	*Felis catus*
0.0703	0.0841	0.0683	0.127	*Tupaia belangeri*
0.0829	0.127	0.107	0.112	*Oryctolagus cuniculus*
0.336	0.364	0.28	0.398	*Ochotona princeps*
0.0429	0.0489	0.0222	0.0388	*Marmota marmota marmota*
0.101	0.102	0.131	0.136	*Ictidomys tridecemlineatus*
0.352	0.5	0.416	0.447	*Octodon degus*
0.158	0.143	0.195	0.183	*Chinchilla lanigera*
0.297	0.39	0.357	0.283	*Cavia porcellus*
0.125	0.114	0.069	0.116	*Heterocephalus glaber*
0.136	0.193	0.139	0.18	*Jaculus jaculus*
0.362	0.466	0.366	0.401	*Rattus norvegicus*
0.378	0.383	0.409	0.451	*Mus musculus*
0.291	0.256	0.271	0.443	*Meriones unguiculatus*
0.273	0.186	0.224	0.343	*Peromyscus maniculatus*
0.435	0.633	0.473	0.528	*Microtus ochrogaster*
0.362	0.561	0.541	0.538	*Mesocricetus auratus*
0.225	0.221	0.181	0.336	*Dipodomys ordii*
0.0708	0.0903	0.0716	0.0659	*Castor canadensis*
0.103	0.197	0.0849	0.151	*Otolemur garnettii*
0.0403	0.0785	0.0421	0.0504	*Propithecus coquereli*
0.101	0.0509	0.0524	0.144	*Microcebus murinus*
0.0229	0.0197	0.0232	0.0201	*Nomascus leucogenys*
0.0231	0.0187	0.0112	0.0145	*Pongo abelii*
0.0239	0.0992	0.0197	0.0292	*Pan troglodytes*
0.0154	0.0131	0.0102	0.0131	*Homo sapiens*
0.0222	0.0158	0.00956	0.0145	*Gorilla gorilla*
0.0392	0.0311	0.018	0.0295	*Chlorocebus sabaeus*
0.0314	0.029	0.0164	0.0242	*Macaca mulatta*
0.0211	0.0224	0.0137	0.0201	*Papio anubis*
0.0245	0.0231	0.0138	0.0232	*Mandrillus leucophaeus*
0.0269	0.0343	0.209	0.0233	*Cercocebus atys*
0.0291	0.0259	0.014	0.0208	*Colobus angolensis*
0.0581	0.0503	0.0673	0.117	*Saimiri boliviensis*
0.0445	0.0498	0.038	0.039	*Cebus capucinus*
0.124	0.0856	0.0996	0.114	*Callithrix jacchus*
0.0472	0.0469	0.0366	0.0677	*Aotus nancymaae*
**34.9**	**70.1**	**88.6**	**151**	**Maximum range**

Table 12: Repeatability of mutation rate (μ) estimation in mammals, for the extant taxa.

Correlation (ρ)	N_e	μ	Maximum longevity	Adult weight	Female maturity
N_e	-	0.439**	−0.523**	−0.544**	−0.47**
μ	-	-	−0.832**	−0.835**	−0.833**
Maximum longevity	-	-	-	0.827**	0.845**
Adult weight	-	-	-	-	0.809**
Female maturity	-	-	-	-	-

Asterisks indicate strength of support of the posterior probability to be different than 0 (pp) as $^{*}pp > 0.95$ and $^{**}pp > 0.975$.

Correlation (ρ)	N_e	μ	Maximum longevity	Adult weight	Female maturity
N_e	-	0.51**	−0.591**	−0.496**	−0.465**
μ	-	-	−0.771**	−0.722**	−0.679**
Maximum longevity	-	-	-	0.802**	0.812**
Adult weight	-	-	-	-	0.764**
Female maturity	-	-	-	-	-

Correlation (ρ)	N_e	μ	Maximum longevity	Adult weight	Female maturity
N_e	-	0.497**	−0.643**	−0.577**	−0.627**
μ	-	-	−0.803**	−0.795**	−0.739**
Maximum longevity	-	-	-	0.836**	0.843**
Adult weight	-	-	-	-	0.805**
Female maturity	-	-	-	-	-

Correlation (ρ)	N_e	μ	Maximum longevity	Adult weight	Female maturity
N_e	-	0.707**	−0.687**	−0.638**	−0.611**
μ	-	-	−0.85**	−0.865**	−0.83**
Maximum longevity	-	-	-	0.839**	0.851**
Adult weight	-	-	-	-	0.817**
Female maturity	-	-	-	-	-

Table 13: In all four replicates, covariance coefficient between effective population size (N_e), mutation rate per site per unit of time (μ), and life-history traits (maximum longevity, adult weight and female maturity) were computed in placental mammals. Asterisks indicate strength of support ($^{*}pp > 0.95$, $^{**}pp > 0.975$).

3.4 Amino-acid preferences entropy

Experiment	$\langle \Omega \rangle$ (branch N_e)	$\langle \Omega \rangle$ (constant N_e)
Mammals 18 CDS, replicate 1, Chain 1	1.07 ± 0.10	1.14 ± 0.10
Mammals 18 CDS, replicate 2, Chain 2	1.07 ± 0.09	1.14 ± 0.10
Mammals 18 CDS, replicate 2, Chain 1	1.06 ± 0.10	1.12 ± 0.09
Mammals 18 CDS, replicate 2, Chain 2	1.06 ± 0.09	1.11 ± 0.10
Mammals 18 CDS, replicate 3, Chain 1	1.08 ± 0.12	1.15 ± 0.11
Mammals 18 CDS, replicate 3, Chain 2	1.04 ± 0.10	1.18 ± 0.11
Mammals 18 CDS, replicate 4, Chain 1	0.94 ± 0.11	1.02 ± 0.12
Mammals 18 CDS, replicate 4, Chain 2	0.89 ± 0.11	1.02 ± 0.11
Mammals 36 CDS, replicate 1, Chain 1	1.02 ± 0.06	1.07 ± 0.10
Mammals 36 CDS, replicate 1, Chain 2	0.91 ± 0.07	1.03 ± 0.07
Mammals 36 CDS, replicate 2, Chain 1	0.92 ± 0.09	0.96 ± 0.09
Mammals 36 CDS, replicate 2, Chain 2	1.01 ± 0.09	1.02 ± 0.11
Mammals 36 CDS, replicate 3, Chain 1	0.93 ± 0.00	1.05 ± 0.09
Mammals 36 CDS, replicate 3, Chain 2	1.02 ± 0.07	1.05 ± 0.11
Mammals 36 CDS, replicate 4, Chain 1	1.04 ± 0.07	1.10 ± 0.08
Mammals 36 CDS, replicate 4, Chain 2	1.03 ± 0.10	1.08 ± 0.08
Mammals 36 CDS, replicate 5, Chain 1	1.03 ± 0.10	1.03 ± 0.08
Mammals 36 CDS, replicate 5, Chain 2	0.99 ± 0.10	1.04 ± 0.08
Mammals 36 CDS, replicate 6, Chain 1	1.05 ± 0.10	1.10 ± 0.08
Mammals 36 CDS, replicate 6, Chain 2	0.97 ± 0.11	1.10 ± 0.10

Table 14: Estimated amino-acid entropy in mammals. Obtained with the mechanistic inference model developed in this paper of site-specific amino-acid fitness profiles and log-Brownian process for N_e, μ and life-history traits (in the left column), or under the assumption of constant N_e (in the right column).

3.5 Identifiability of N_e and μ

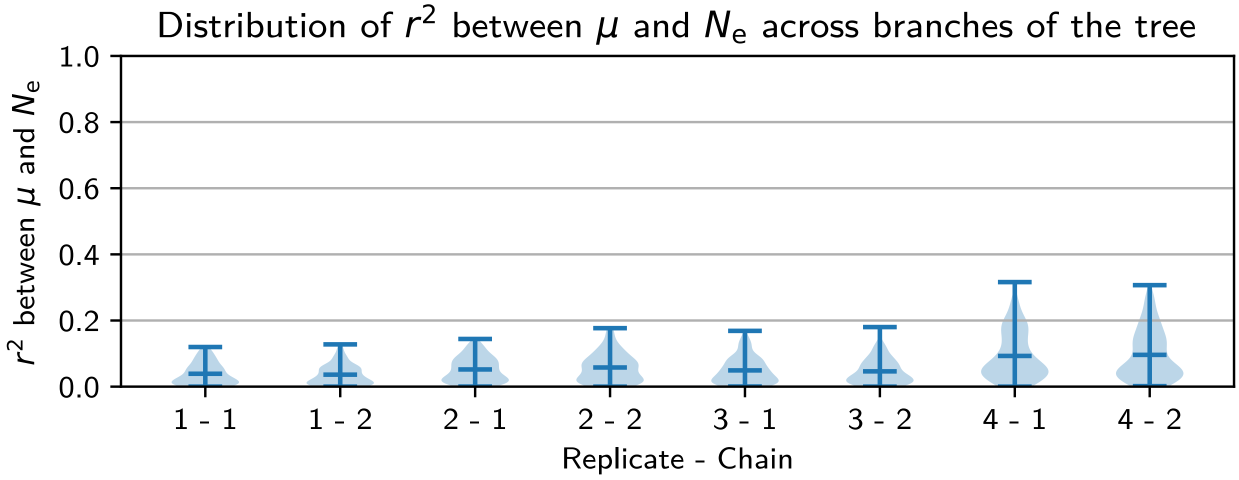

Figure 18: Some parameters might be strongly correlated or only weakly identifiable, which may make it hard to fit the model to realistically sized datasets. To test whether N_e and μ are identifiable, for each branch of the tree we draw a 2-D scatter plot for N_e and μ, where each point is a step in the MCMC procedure. We subsequently fit a linear regression and compute the coefficient of determination (r^2) for each branch of the tree. For a given MCMC, the distribution of r^2 across all branches is then represented as a violin plot. The distribution of r^2 is relatively low, below 0.05 on average, providing confidence that N_e and μ are indeed identifiable.

3.6 Traits estimation with branch ω (replicate 1, chain 1)

Obtained with the phenomenological inference model of log-Brownian process for the μ and the relative non-synonymous substitution rate (ω), as in Lartillot and Poujol (2011).

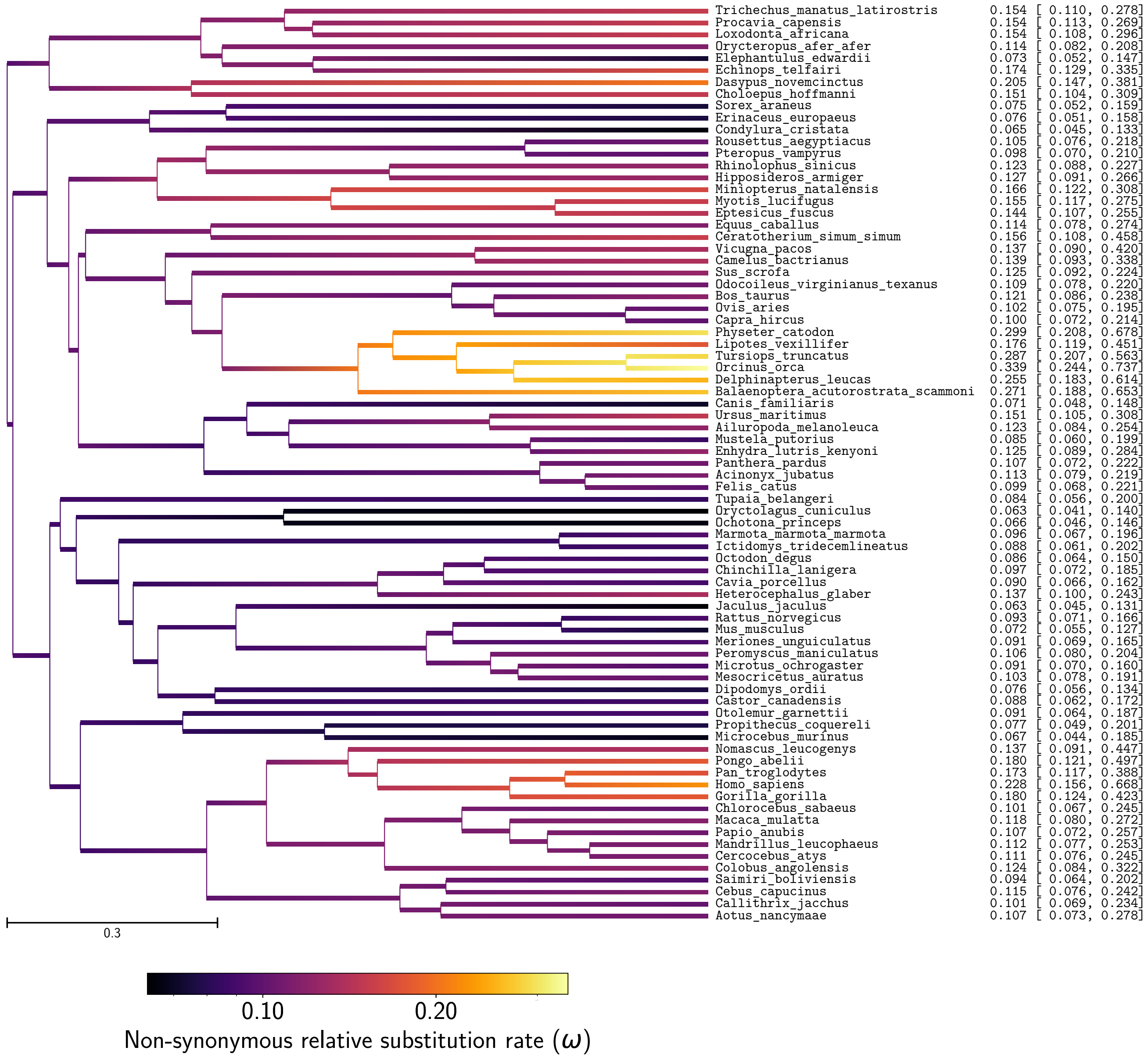

Figure 19: Non-synonymous substitution rate (ω) estimation in mammals

Correlation (ρ)	ω	μ	Maximum longevity	Adult weight	Female maturity
ω	-	-0.374^{**}	0.544^{**}	0.43^{**}	0.433^{**}
μ	-	-	-0.807^{**}	-0.781^{**}	-0.824^{**}
Maximum longevity	-	-	-	0.801^{**}	0.83^{**}
Adult weight	-	-	-	-	0.785^{**}
Female maturity	-	-	-	-	-

Table 15: Correlation coefficient between non-synonymous substitution rate (ω), mutation rate per site per unit of time (μ), and life-history traits (maximum longevity, adult weight and female maturity) were computed in placental mammals. Asterisks indicate strength of support ($^{*}pp > 0.95$, $^{**}pp > 0.975$).

Covariance (Σ)	ω	μ	Maximum longevity	Adult weight	Female maturity
ω	0.215^{**}	-0.236^{**}	0.231^{**}	0.828^{**}	0.242^{**}
μ	-	1.82^{**}	-0.998^{**}	-4.38^{**}	-1.34^{**}
Maximum longevity	-	-	0.837^{**}	3.04^{**}	0.917^{**}
Adult weight	-	-	-	17.1^{**}	3.93^{**}
Female maturity	-	-	-	-	1.45^{**}

Table 16: Correlation coefficient between non-synonymous substitution rate (ω), mutation rate per site per unit of time (μ), and life-history traits (maximum longevity, adult weight and female maturity) were computed in placental mammals. Asterisks indicate strength of support ($^{*}pp > 0.95$, $^{**}pp > 0.975$).

Partial coefficient	ω	μ	Maximum longevity	Adult weight	Female maturity
ω	-	0.15	0.369^{**}	0.0468	0.0223
μ	-	-	-0.299^{*}	-0.272	-0.382^{**}
Maximum longevity	-	-	-	0.283^{**}	0.338^{**}
Adult weight	-	-	-	-	0.21^{*}
Female maturity	-	-	-	-	-

Table 17: Partial correlation coefficient between non-synonymous substitution rate (ω), mutation rate per site per unit of time (μ), and life-history traits (maximum longevity, adult weight and female maturity) were computed in placental mammals. Asterisks indicate strength of support ($^{*}pp > 0.95$, $^{**}pp > 0.975$).

4 Empirical data in Isopods

Only highly conserved coding sequences are kept for the analysis, representing 135 genes with $\leq 1\%$ of gaps in the alignment. The list of genes included in the analysis is (also available at `https://github.com/ThibaultLatrille/MutationSelectionDrift/blob/master/DataEmpirical/Isopods/cds.highcoverage.list`):
OG0009934 OG0009413 OG0008719 OG0010498 OG0005374 OG0004371 OG0008366 OG0007077 OG0010145 OG0011096 OG0011154 OG0007043 OG0006976 OG0010459 OG0010974 OG0007865 OG0011750 OG0007706 OG0007268 OG0008501 OG0009198 OG0004682 OG0011492 OG0009044 OG0005946 OG0011402 OG0008807 OG0004169 OG0006035 OG0005579 OG0011650 OG0005948 OG0010501 OG0009824 OG0005652 OG0011517 OG0009297 OG0008919 OG0006573 OG0004302 OG0008485 OG0009943 OG0009497 OG0011176 OG0008899 OG0006461 OG0006871 OG0010237 OG0007494 OG0010468 OG0005753 OG0008734 OG0010865 OG0010010 OG0010156 OG0009652 OG0006260 OG0010263 OG0007705 OG0008446 OG0011177 OG0010681 OG0006201 OG0011223 OG0010617 OG0009858 OG0009138 OG0007202 OG0009298 OG0004981 OG0005765 OG0006517 OG0005408 OG0008903 OG0009605 OG0009949 OG0011307 OG0008656 OG0005828 OG0010434 OG0011525 OG0008406 OG0008505 OG0007143 OG0002475 OG0008488 OG0009361 OG0011140 OG0008606 OG0011397 OG0009410 OG0005754 OG0010169 OG0010987 OG0005587 OG0010234 OG0010223 OG0009544 OG0011945 OG0010902 OG0008723 OG0010892 OG0009142 OG0011172 OG0009593 OG0008603 OG0008107 OG0006015 OG0011504 OG0004368 OG0002002 OG0009926 OG0009312 OG0008943 OG0007396 OG0009814 OG0007404 OG0012129 OG0005644 OG0011416 OG0008687 OG0009379 OG0010350 OG0011214 OG0006824 OG0008097 OG0009225 OG0007507 OG0009314 OG0008843 OG0007415 OG0009532 OG0008630 OG0009475 OG0005651

6 independent inferences were performed on a randomly chosen set of 12 coding sequences (CDS) out of the 135.

The replicate 1 is composed of :
OG0008097 OG0009298 OG0008719 OG0010501 OG0011650 OG0011096 OG0008603 OG0008899 OG0006573 OG0010498 OG0011750 OG0009138

The replicate 2 is composed of :
OG0009225 OG0011750 OG0009605 OG0011945 OG0008843 OG0011525 OG0009814 OG0008485 OG0006871 OG0009544 OG0010974 OG0009824

The replicate 3 is composed of :
OG0009379 OG0010892 OG0009949 OG0010865 OG0008630 OG0011172 OG0011154 OG0010501 OG0007706 OG0009532 OG0008488 OG0005651

The replicate 4 is composed of :
OG0008899 OG0010169 OG0010892 OG0012129 OG0010350 OG0009593 OG0007268 OG0002475 OG0005753 OG0006573 OG0010987 OG0008943

The replicate 5 is composed of :
OG0005828 OG0005587 OG0006824 OG0007706 OG0010350 OG0011402 OG0008488 OG0009943 OG0005408 OG0008603 OG0010459 OG0005765

The replicate 6 is composed of :
OG0006824 OG0006260 OG0006871 OG0010010 OG0006517 OG0009593 OG0005754 OG0011214 OG0002002 OG0009314 OG0011096 OG0004371

4.1 Traits estimation (replicate 1, chain 1)

Obtained with the mechanistic inference model developed in this paper of site-specific amino-acid fitness profiles and log-Brownian process for N_{e}, μ and life-history traits.

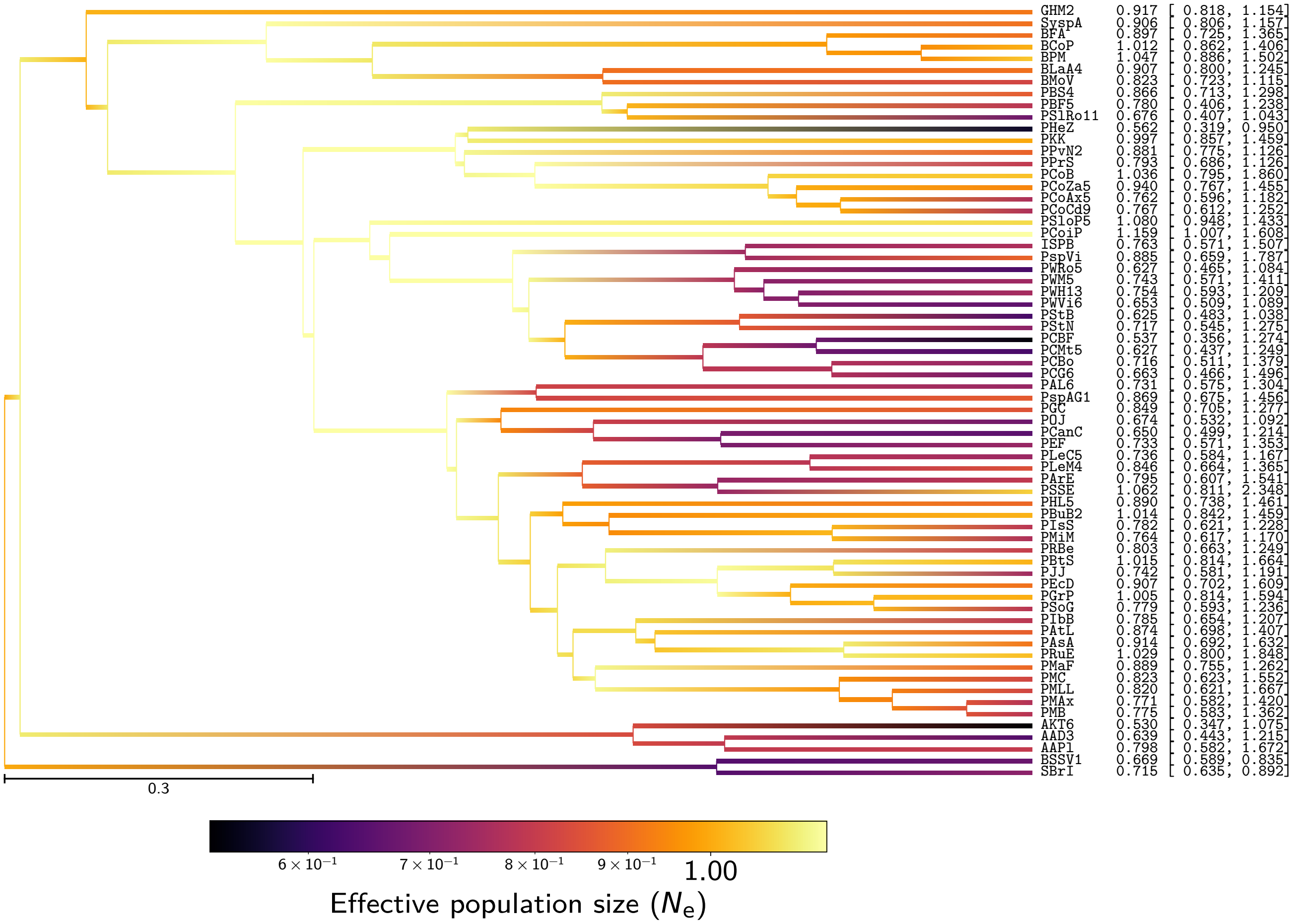

Figure 20: Effective population size (N_e) estimation in isopods

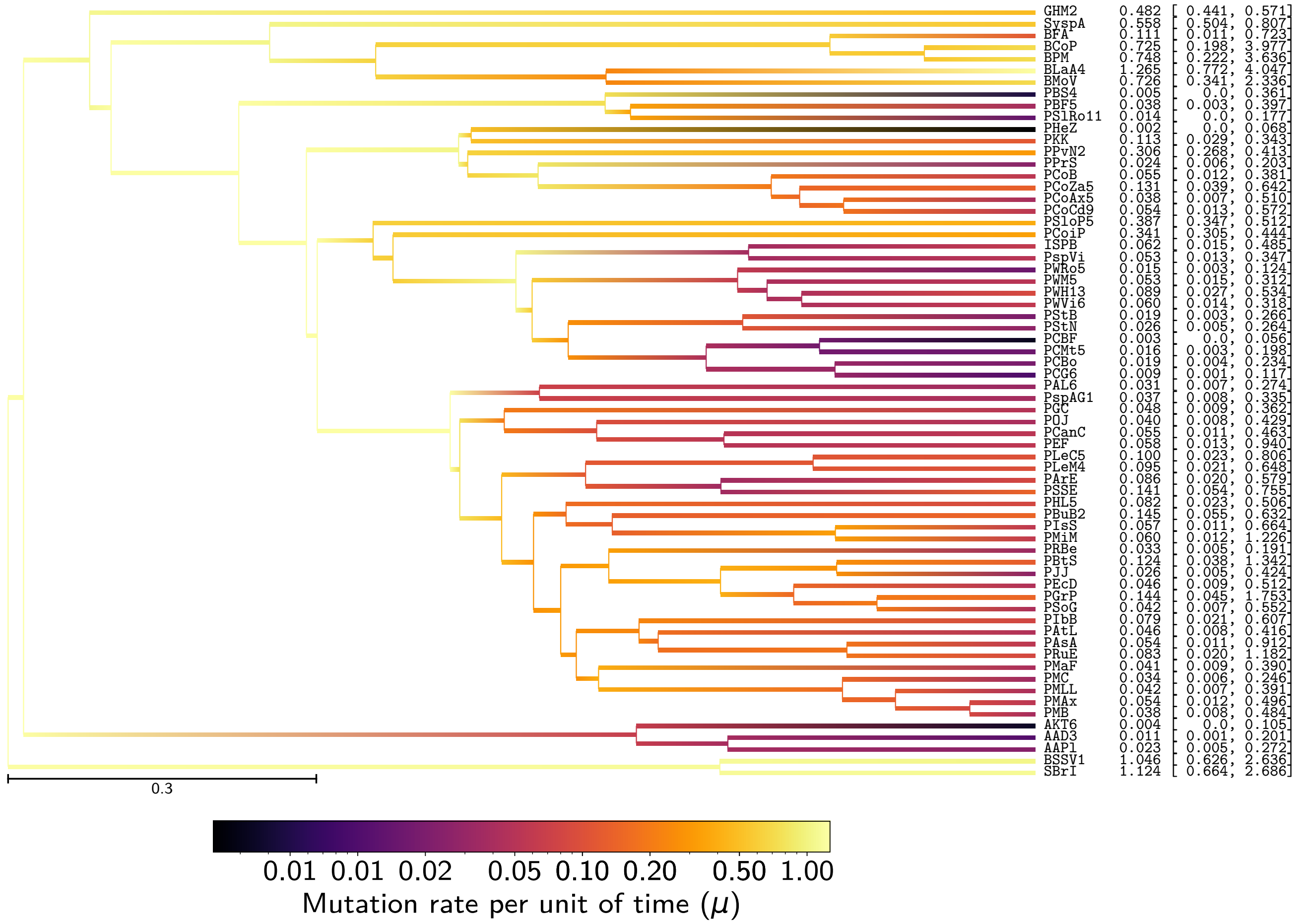

Figure 21: Mutation rate (μ) estimation in isopods

4.2 Repeatability of experiments

6 independent inferences were performed on a randomly chosen set of 12 coding sequences (CDS) out of 135. Obtained with the mechanistic inference model developed in this paper of site-specific amino-acid fitness profiles and log-Brownian process for N_e, μ. Each plot is a correlation between a pair of experiments for a given parameter. For each node (or branch) of the tree, the mean posterior of the parameter over the MCMC (after burn-in) is represented in blue dots, green solid lines are the 90% confidence interval of the MCMC. Solid red line is the regression line between replicates.

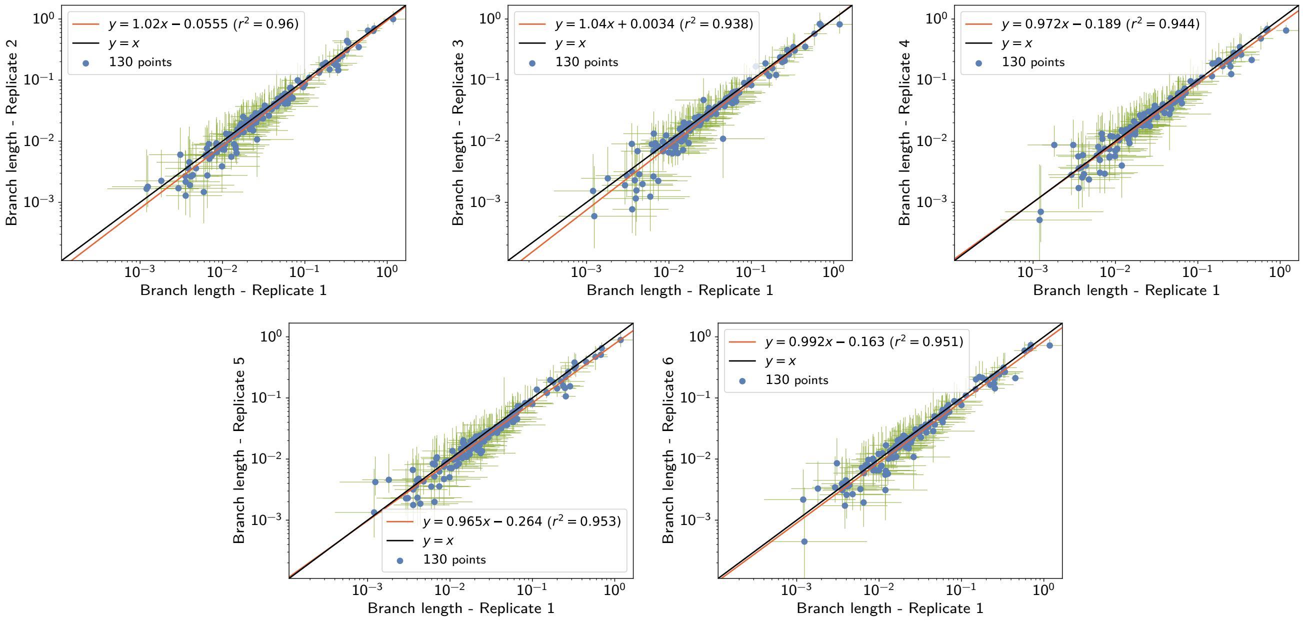

Figure 22: Repeatability of branch length (l) estimation in isopods

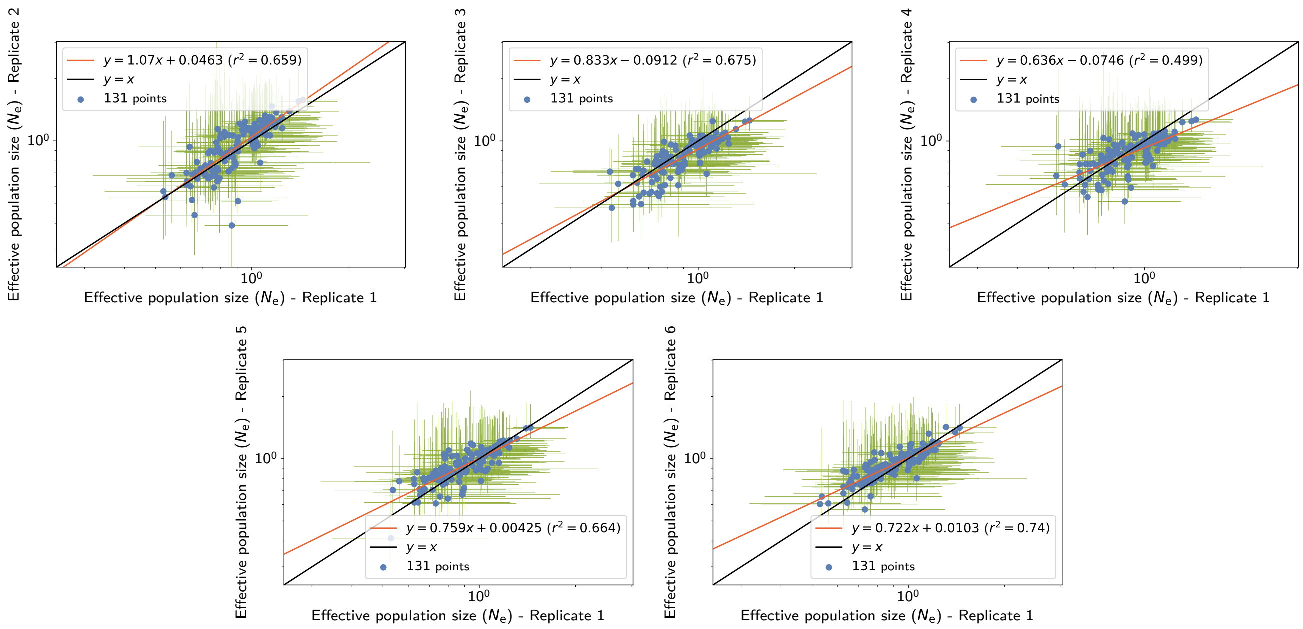

Figure 23: Repeatability of effective population size (N_e) estimation in isopods

Rep. 1	Rep. 2	Rep. 3	Rep. 4	Rep. 5	Rep. 6	Habitat	Pigmentation	Ocular structure	Code	Taxon
0.917	1.21	0.91	1.09	1.1	0.997	Underground	Depigmented	Anophthalmia	GHM2	*Gallasellus heyli*
0.906	0.511	0.876	0.907	0.948	0.964	Underground	Depigmented	Anophthalmia	SyspA	*Synasellus sp*
0.897	0.954	0.838	0.819	0.721	0.859	Underground	Depigmented	Anophthalmia	BFA	*Bragasellus frontellum*
1.01	1.15	0.78	0.894	0.78	0.945	Surface	Pigmented	Ocular	BCoP	*Bragasellus cortesi*
1.05	0.95	0.881	0.815	0.87	1.08	Surface	Pigmented	Ocular	BPM	*Bragasellus peltatus*
0.907	0.891	0.769	0.831	0.873	0.907	Underground	Depigmented	Anophthalmia	BLaA4	-
0.823	0.797	0.839	0.949	0.852	1.06	Underground	Depigmented	Anophthalmia	BMoV	*Bragasellus molinai*
0.866	0.39	0.61	0.51	0.773	0.871	Underground	Depigmented	Anophthalmia	PBS4	*Proasellus boui*
0.78	0.637	0.702	0.808	0.919	0.884	Underground	Depigmented	Anophthalmia	PBF5	*Proasellus boui*
0.676	0.657	0.718	0.895	0.777	0.884	Underground	Depigmented	Anophthalmia	PSlRo11	*Proasellus slavus*
0.562	0.681	0.62	0.616	0.778	0.608	Underground	Depigmented	Anophthalmia	PHeZ	*Proasellus hercegovinensis*
0.997	1.15	0.962	1.08	0.907	1.06	Surface	Pigmented	Ocular	PKK	*Proasellus karamani*
0.881	0.897	0.753	0.746	0.877	0.901	Underground	Depigmented	Anophthalmia	PPvN2	*Proasellus pavani*
0.793	0.66	0.876	0.711	0.813	0.808	Underground	Depigmented	Anophthalmia	PPrS	*Proasellus parvulus*
1.04	1.06	1.06	0.968	1.05	0.957	Surface	Pigmented	Ocular	PCoB	*Proasellus coxalis*
0.94	1.17	1.06	0.937	1.18	1.14	Surface	Pigmented	Ocular	PCoZa5	*Proasellus coxalis*
0.762	0.595	0.792	0.731	0.902	0.657	Underground	Depigmented	Anophthalmia	PCoAx5	*Proasellus coxalis*
0.767	0.705	0.711	0.848	0.847	0.761	Underground	Depigmented	Microphthalmia	PCoCd9	*Proasellus coxalis*
1.08	1.09	0.893	0.975	1.1	0.968	Underground	Depigmented	Anophthalmia	PSloP5	*Proasellus slovenicus*
1.16	1.28	1.02	1.07	1.21	1.32	Surface	Pigmented	Ocular	PCoiP	*Proasellus coiffaiti*
0.763	0.888	0.753	0.709	0.766	0.86	Underground	Depigmented	Anophthalmia	ISPB	*Proasellus nsp*
0.885	0.789	0.765	0.675	0.67	0.857	Underground	Depigmented	Anophthalmia	PspVi	*Proasellus nsp*
0.627	0.636	0.493	0.771	0.754	0.732	Underground	Depigmented	Anophthalmia	PWRo5	*Proasellus walteri*
0.743	0.671	0.558	1.03	0.834	0.761	Underground	Depigmented	Anophthalmia	PWM5	*Proasellus walteri*
0.754	0.718	0.54	0.79	0.656	0.824	Underground	Depigmented	Anophthalmia	PWH13	*Proasellus walteri*
0.653	0.689	0.538	0.774	0.667	0.764	Underground	Depigmented	Anophthalmia	PWVi6	*Proasellus walteri*
0.625	0.701	0.682	0.703	0.796	0.875	Underground	Depigmented	Anophthalmia	PStB	*Proasellus strouhali*
0.717	0.693	0.682	0.582	0.839	0.734	Underground	Depigmented	Anophthalmia	PStN	*Proasellus strouhali*
0.537	0.535	0.475	0.941	0.705	0.656	Underground	Depigmented	Anophthalmia	PCBF	*Proasellus cavaticus*
0.627	0.611	0.512	0.659	0.614	0.68	Underground	Depigmented	Anophthalmia	PCMt5	*Proasellus cavaticus*
0.716	0.761	0.632	0.603	0.844	0.747	Underground	Depigmented	Anophthalmia	PCBo	*Proasellus cavaticus*
0.663	0.437	0.495	0.535	0.689	0.793	Underground	Depigmented	Anophthalmia	PCG6	*Proasellus cavaticus*
0.731	0.668	0.778	0.805	0.608	0.568	Underground	Depigmented	Anophthalmia	PAL6	*Proasellus albigensis*
0.869	0.737	0.729	0.92	0.827	0.896	Underground	Depigmented	Anophthalmia	PspAG1	*Proasellus n*
0.849	0.839	0.95	0.966	0.931	0.94	Underground	Depigmented	Anophthalmia	PGC	*Proasellus grafi*
0.674	0.79	0.719	0.772	0.784	0.826	Surface	Part. dep.	Microphthalmia	POJ	*Proasellus ortizi*
0.65	0.517	0.732	0.613	0.711	0.729	Underground	Depigmented	Anophthalmia	PCanC	*Proasellus cantabricus*
0.733	0.659	0.684	0.579	0.795	0.802	Surface	Part. dep.	Microphthalmia	PEF	*Proasellus ebrensis*
0.736	0.743	0.8	0.685	0.805	0.935	Underground	Depigmented	Anophthalmia	PLeC5	-
0.846	0.711	0.785	0.864	0.957	0.889	Underground	Depigmented	Anophthalmia	PLeM4	-
0.795	1.03	0.846	0.87	0.869	0.851	Surface	Part. dep.	Microphthalmia	PArE	*Proasellus aragonensis*
1.06	0.785	0.694	0.756	0.893	0.804	Underground	Depigmented	Anophthalmia	PSSE	*Proasellus spelaeus*
0.89	0.774	0.727	0.766	0.926	0.992	Underground	Depigmented	Anophthalmia	PHL5	-
1.01	0.994	0.783	0.789	1.14	1.17	Underground	Depigmented	Anophthalmia	PBuB2	-
0.782	1.14	0.991	0.853	1	1.07	Surface	Pigmented	Ocular	PIsS	*Proasellus istrianus*
0.764	0.878	0.853	0.736	0.887	0.966	Surface	Pigmented	Ocular	PMiM	*Proasellus micropectinatus*
0.803	1.08	0.819	0.96	1.1	0.823	Surface	Part. dep.	Microphthalmia	PRBe	*Proasellus racovitzai*
1.01	1.1	0.905	0.931	0.884	1.01	Surface	Pigmented	Ocular	PBtS	*Proasellus beticus*
0.742	0.896	0.842	0.826	0.84	0.882	Underground	Depigmented	Microphthalmia	PJJ	*Proasellus jaloniacus*
0.907	1.04	0.792	0.594	0.859	0.836	Underground	Depigmented	Anophthalmia	PEcD	*Proasellus escolai*
1.01	1.02	0.86	0.786	1.06	0.922	Surface	Part. dep.	Microphthalmia	PGrP	*Proasellus granadensis*
0.779	0.738	0.707	0.731	0.812	0.808	Underground	Depigmented	Anophthalmia	PSoG	*Proasellus solanasi*
0.785	0.918	0.854	0.788	1.04	0.986	Surface	Pigmented	Ocular	PIbB	*Proasellus ibericus*
0.874	0.836	0.764	0.815	0.866	1.05	Underground	Depigmented	Anophthalmia	PAtL	*Proasellus arthrodilus*
0.914	0.951	0.888	0.834	0.881	0.886	Surface	Part. dep.	Microphthalmia	PAsA	*Proasellus assaforensis*
1.03	0.994	0.939	0.854	0.971	1	Underground	Depigmented	Anophthalmia	PRuE	*Proasellus rectus*
0.889	0.764	0.761	0.651	0.698	0.847	Underground	Depigmented	Anophthalmia	PMaF	*Proasellus margalefi*
0.823	1.1	0.961	0.914	1.03	0.838	Surface	Pigmented	Ocular	PMC	*Proasellus meridianus*
0.82	1.05	0.806	0.914	0.856	0.799	Surface	Pigmented	Ocular	PMLL	*Proasellus meridianus*
0.771	0.889	0.796	0.907	0.839	0.863	Underground	Part. dep.	Microphthalmia	PMAx	*Proasellus meridianus*
0.775	0.982	0.892	1.02	1.05	0.882	Surface	Pigmented	Ocular	PMB	*Proasellus meridianus*
0.53	0.57	0.71	0.679	0.413	0.603	Underground	Depigmented	Anophthalmia	AKT6	*Asellus kosswigi*
0.639	0.936	0.732	0.859	0.859	0.866	Surface	Pigmented	Ocular	AAD3	*Asellus aquaticus*
0.798	0.882	0.795	0.89	0.641	0.875	Surface	Pigmented	Ocular	AAPl	*Asellus aquaticus*
0.669	0.704	0.684	0.655	0.805	0.711	Underground	Depigmented	Anophthalmia	BSSV1	*Balkanostenasellus skopljensis*
0.715	0.682	0.685	0.614	0.707	0.77	Underground	Depigmented	Anophthalmia	SBrI	*Stenasellus breuili*
**2.19**	**3.29**	**2.24**	**2.13**	**2.93**	**2.33**	-	-	-	-	**Maximum range**

Table 18: Repeatability of effective population size (N_{e}) estimation in isopods, for the extant taxa.

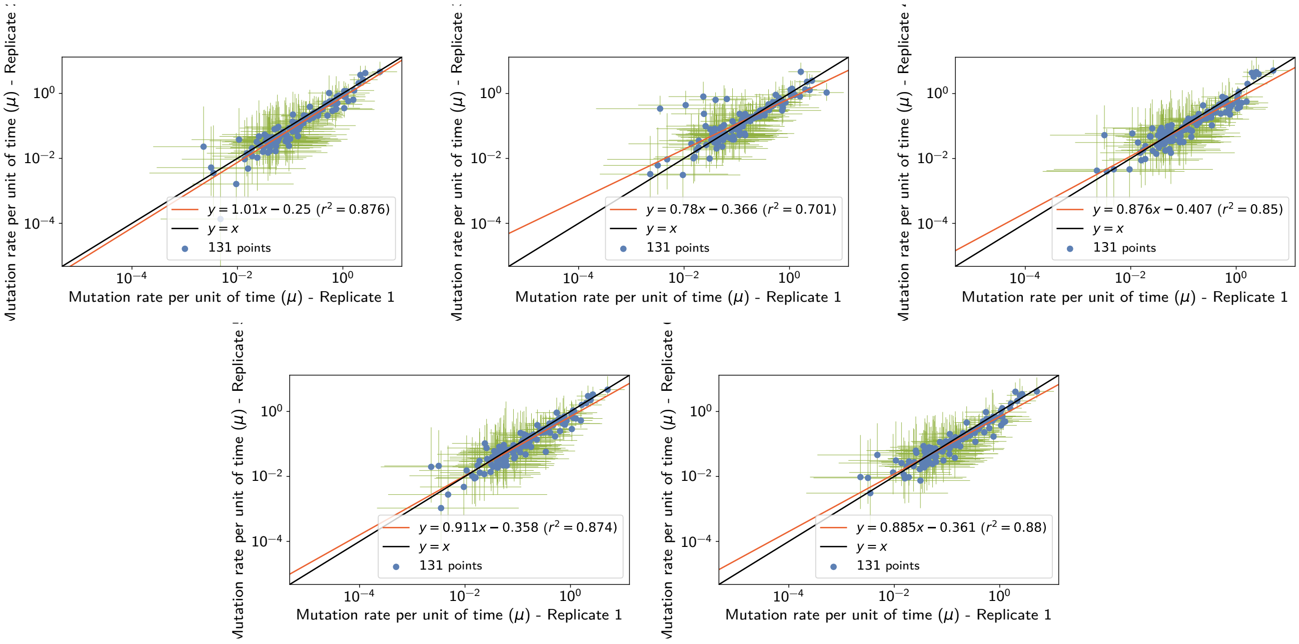

Figure 24: Repeatability of mutation rate (μ) estimation in isopods

Rep. 1	Rep. 2	Rep. 3	Rep. 4	Rep. 5	Rep. 6	Habitat	Pigmentation	Ocular structure	Code	Taxon
0.482	0.488	0.54	0.462	0.456	0.497	Underground	Depigmented	Anophthalmia	GHM2	*Gallasellus heyli*
0.558	0.563	0.536	0.52	0.503	0.59	Underground	Depigmented	Anophthalmia	SyspA	*Synasellus sp*
0.111	0.0674	0.0703	0.0781	0.0555	0.0852	Underground	Depigmented	Anophthalmia	BFA	*Bragasellus frontellum*
0.725	0.32	0.3	0.377	0.421	0.579	Surface	Pigmented	Ocular	BCoP	*Bragasellus cortesi*
0.748	0.337	0.364	0.331	0.427	0.692	Surface	Pigmented	Ocular	BPM	*Bragasellus peltatus*
1.27	0.579	0.893	0.516	0.607	0.49	Underground	Depigmented	Anophthalmia	BLaA4	-
0.726	0.462	0.442	0.463	0.428	0.519	Underground	Depigmented	Anophthalmia	BMoV	*Bragasellus molinai*
0.00478	0.000136	0.00937	0.00462	0.00277	0.046	Underground	Depigmented	Anophthalmia	PBS4	*Proasellus boui*
0.038	0.0347	0.0822	0.0873	0.048	0.0628	Underground	Depigmented	Anophthalmia	PBF5	*Proasellus boui*
0.0137	0.00942	0.0281	0.031	0.0105	0.0259	Underground	Depigmented	Anophthalmia	PSlRo11	*Proasellus slavus*
0.00228	0.0229	0.00329	0.00422	0.0197	0.00957	Underground	Depigmented	Anophthalmia	PHeZ	*Proasellus hercegovinensis*
0.113	0.224	0.235	0.103	0.218	0.202	Surface	Pigmented	Ocular	PKK	*Proasellus karamani*
0.306	0.25	0.257	0.225	0.229	0.23	Underground	Depigmented	Anophthalmia	PPvN2	*Proasellus pavani*
0.0238	0.0105	0.235	0.0472	0.104	0.0275	Underground	Depigmented	Anophthalmia	PPrS	*Proasellus parvulus*
0.0545	0.0359	0.0712	0.0458	0.073	0.0453	Surface	Pigmented	Ocular	PCoB	*Proasellus coxalis*
0.131	0.121	0.213	0.149	0.198	0.141	Surface	Pigmented	Ocular	PCoZa5	*Proasellus coxalis*
0.0381	0.015	0.0518	0.0305	0.0522	0.0356	Underground	Depigmented	Anophthalmia	PCoAx5	*Proasellus coxalis*
0.0544	0.0332	0.0673	0.0728	0.0931	0.0672	Underground	Depigmented	Microphthalmia	PCoCd9	*Proasellus coxalis*
0.387	0.328	0.364	0.348	0.3	0.317	Underground	Depigmented	Anophthalmia	PSloP5	*Proasellus slovenicus*
0.341	0.28	0.296	0.246	0.0573	0.253	Surface	Pigmented	Ocular	PCoiP	*Proasellus coiffaiti*
0.062	0.0427	0.0495	0.0556	0.0217	0.0281	Underground	Depigmented	Anophthalmia	ISPB	*Proasellus nsp*
0.0533	0.0419	0.0549	0.0516	0.0228	0.0245	Underground	Depigmented	Anophthalmia	PspVi	*Proasellus nsp*
0.0151	0.0144	0.0101	0.015	0.00874	0.0115	Underground	Depigmented	Anophthalmia	PWRo5	*Proasellus walteri*
0.0531	0.0167	0.0231	0.0911	0.0218	0.0243	Underground	Depigmented	Anophthalmia	PWM5	*Proasellus walteri*
0.0886	0.0317	0.0384	0.0491	0.0402	0.0934	Underground	Depigmented	Anophthalmia	PWH13	*Proasellus walteri*
0.0597	0.093	0.0527	0.106	0.0297	0.0383	Underground	Depigmented	Anophthalmia	PWVi6	*Proasellus walteri*
0.019	0.0117	0.0353	0.0242	0.0131	0.0291	Underground	Depigmented	Anophthalmia	PStB	*Proasellus strouhali*
0.0263	0.0262	0.026	0.0173	0.0345	0.0534	Underground	Depigmented	Anophthalmia	PStN	*Proasellus strouhali*
0.00317	0.00521	0.00612	0.0523	0.0211	0.00902	Underground	Depigmented	Anophthalmia	PCBF	*Proasellus cavaticus*
0.0159	0.00485	0.0112	0.00879	0.00919	0.00876	Underground	Depigmented	Anophthalmia	PCMt5	*Proasellus cavaticus*
0.0188	0.0205	0.0221	0.00963	0.0302	0.00951	Underground	Depigmented	Anophthalmia	PCBo	*Proasellus cavaticus*
0.0095	0.00162	0.00309	0.00461	0.00475	0.0132	Underground	Depigmented	Anophthalmia	PCG6	*Proasellus cavaticus*
0.0314	0.021	0.0791	0.0263	0.0238	0.00749	Underground	Depigmented	Anophthalmia	PAL6	*Proasellus albigensis*
0.0372	0.0313	0.073	0.0375	0.0464	0.0191	Underground	Depigmented	Anophthalmia	PspAG1	*Proasellus n*
0.0477	0.0403	0.0817	0.0666	0.0594	0.0425	Underground	Depigmented	Anophthalmia	PGC	*Proasellus grafi*
0.0404	0.03	0.0467	0.0547	0.0285	0.0343	Surface	Part. dep.	Microphthalmia	POJ	*Proasellus ortizi*
0.0551	0.0385	0.0367	0.0343	0.0369	0.052	Underground	Depigmented	Anophthalmia	PCanC	*Proasellus cantabricus*
0.0578	0.0337	0.0446	0.0149	0.0349	0.0499	Surface	Part. dep.	Microphthalmia	PEF	*Proasellus ebrensis*
0.1	0.0399	0.0968	0.0322	0.0743	0.104	Underground	Depigmented	Anophthalmia	PLeC5	-
0.095	0.0243	0.0793	0.0904	0.0706	0.12	Underground	Depigmented	Anophthalmia	PLeM4	-
0.0856	0.0426	0.0472	0.0317	0.0361	0.0311	Surface	Part. dep.	Microphthalmia	PArE	*Proasellus aragonensis*
0.141	0.0482	0.0583	0.0461	0.0474	0.0372	Underground	Depigmented	Anophthalmia	PSSE	*Proasellus spelaeus*
0.0822	0.0338	0.0387	0.0355	0.0522	0.059	Underground	Depigmented	Anophthalmia	PHL5	-
0.145	0.0777	0.052	0.0705	0.0868	0.132	Underground	Depigmented	Anophthalmia	PBuB2	-
0.0573	0.0857	0.106	0.0794	0.0706	0.0643	Surface	Pigmented	Ocular	PIsS	*Proasellus istrianus*
0.0599	0.0387	0.0584	0.0471	0.0606	0.0728	Surface	Pigmented	Ocular	PMiM	*Proasellus micropectinatus*
0.0328	0.0543	0.0365	0.052	0.0391	0.0289	Surface	Part. dep.	Microphthalmia	PRBe	*Proasellus racovitzai*
0.124	0.0797	0.15	0.168	0.128	0.12	Surface	Pigmented	Ocular	PBtS	*Proasellus beticus*
0.0256	0.0475	0.0985	0.0846	0.0751	0.0621	Underground	Depigmented	Microphthalmia	PJJ	*Proasellus jaloniacus*
0.0455	0.03	0.0441	0.0175	0.0339	0.0279	Underground	Depigmented	Anophthalmia	PEcD	*Proasellus escolai*
0.144	0.102	0.234	0.163	0.185	0.161	Surface	Part. dep.	Microphthalmia	PGrP	*Proasellus granadensis*
0.0423	0.028	0.0529	0.0454	0.0455	0.0635	Underground	Depigmented	Anophthalmia	PSoG	*Proasellus solanasi*
0.0788	0.0552	0.0724	0.0547	0.0686	0.0646	Surface	Pigmented	Ocular	PIbB	*Proasellus ibericus*
0.0458	0.0447	0.0517	0.0443	0.0601	0.0791	Underground	Depigmented	Anophthalmia	PAtL	*Proasellus arthrodilus*
0.0543	0.0351	0.0573	0.0478	0.065	0.0503	Surface	Part. dep.	Microphthalmia	PAsA	*Proasellus assaforensis*
0.083	0.0536	0.139	0.0608	0.0918	0.0674	Underground	Depigmented	Anophthalmia	PRuE	*Proasellus rectus*
0.0413	0.0168	0.0202	0.0369	0.0292	0.0359	Underground	Depigmented	Anophthalmia	PMaF	*Proasellus margalefi*
0.0343	0.034	0.0559	0.0219	0.0199	0.0235	Surface	Pigmented	Ocular	PMC	*Proasellus meridianus*
0.0416	0.0215	0.0337	0.0312	0.0187	0.0231	Surface	Pigmented	Ocular	PMLL	*Proasellus meridianus*
0.0542	0.0241	0.044	0.0194	0.0247	0.0486	Underground	Part. dep.	Microphthalmia	PMAx	*Proasellus meridianus*
0.0385	0.0252	0.0807	0.0782	0.0541	0.06	Surface	Pigmented	Ocular	PMB	*Proasellus meridianus*
0.00351	0.00353	0.34	0.00401	0.00106	0.00307	Underground	Depigmented	Anophthalmia	AKT6	*Asellus kosswigi*
0.0107	0.0378	0.438	0.0589	0.0152	0.031	Surface	Pigmented	Ocular	AAD3	*Asellus aquaticus*
0.0232	0.0321	0.802	0.048	0.0118	0.0209	Surface	Pigmented	Ocular	AAPl	*Asellus aquaticus*
1.05	0.901	1.31	0.585	0.841	0.711	Underground	Depigmented	Anophthalmia	BSSV1	*Balkanostenasellus skopljensis*
1.12	0.784	1.11	0.479	0.641	0.701	Underground	Depigmented	Anophthalmia	SBrI	*Stenasellus breuili*
**554**	**6.63e+03**	**423**	**146**	**792**	**231**	-	-	-	-	**Maximum range**

Table 19: Repeatability of mutation rate (μ) estimation in isopods, for the extant taxa.

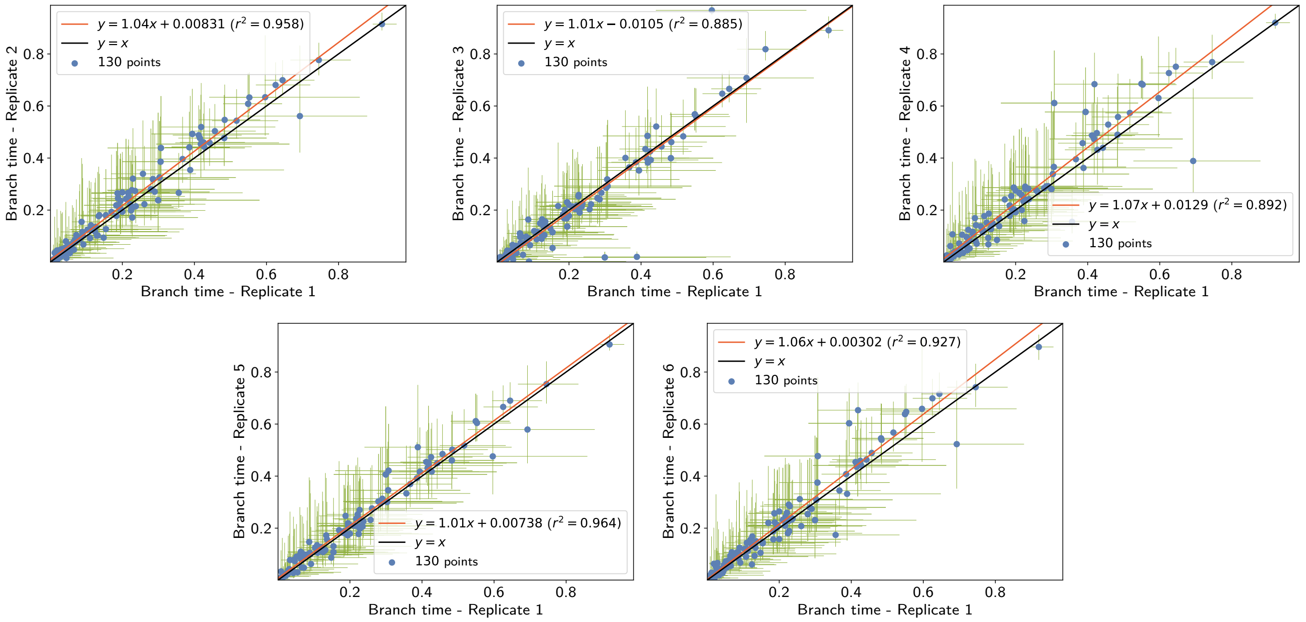

Figure 25: Repeatability of branch time (ΔT) estimation in isopods

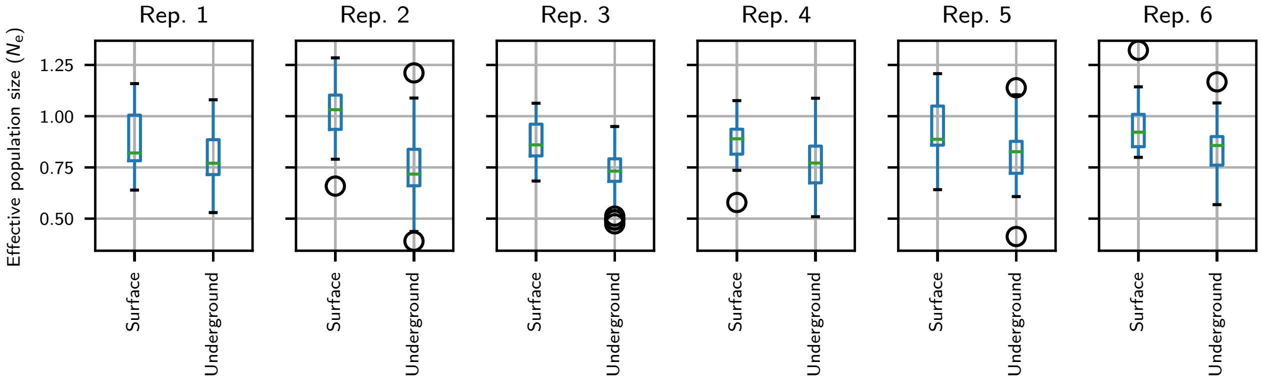

Figure 26: N_e as a function of habitat in isopods.

```
Analysis of Variance Table

Response: PopulationSize
           Df Sum Sq Mean Sq F value    Pr(>F)
Habitat     1 1.6777 1.67769  89.506 < 2.2e-16 ***
Rep         5 0.4226 0.08452   4.509 0.0005236 ***
Residuals 389 7.2913 0.01874
---
Signif. codes:  0 ‘***’ 0.001 ‘**’ 0.01 ‘*’ 0.05 ‘.’ 0.1 ‘ ’ 1
```

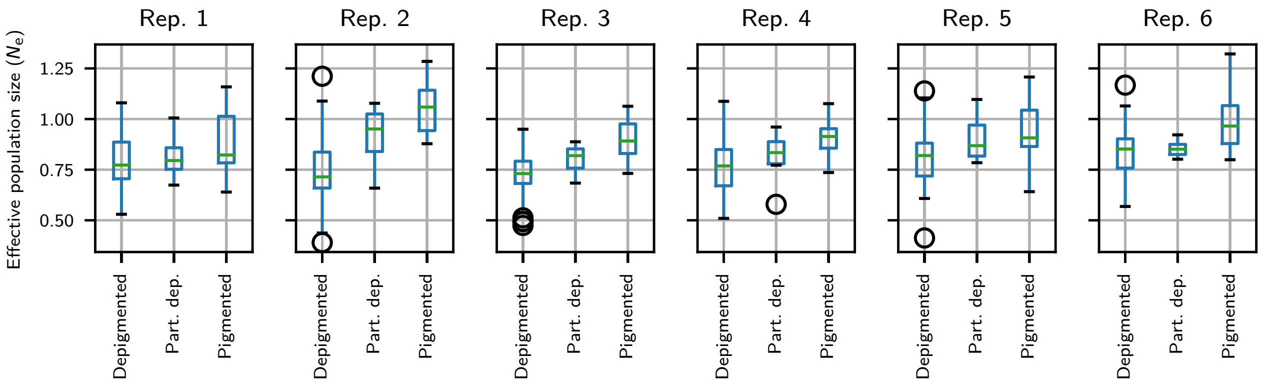

Figure 27: N_e as a function of pigmentation in isopods

```
Analysis of Variance Table

Response: PopulationSize
              Df Sum Sq Mean Sq F value    Pr(>F)
Pigmentation   2 1.9442 0.97210 53.6917 < 2.2e-16 ***
Rep            5 0.4226 0.08452  4.6681 0.0003764 ***
Residuals    388 7.0248 0.01811
---
Signif. codes:  0 ‘***’ 0.001 ‘**’ 0.01 ‘*’ 0.05 ‘.’ 0.1 ‘ ’ 1
```

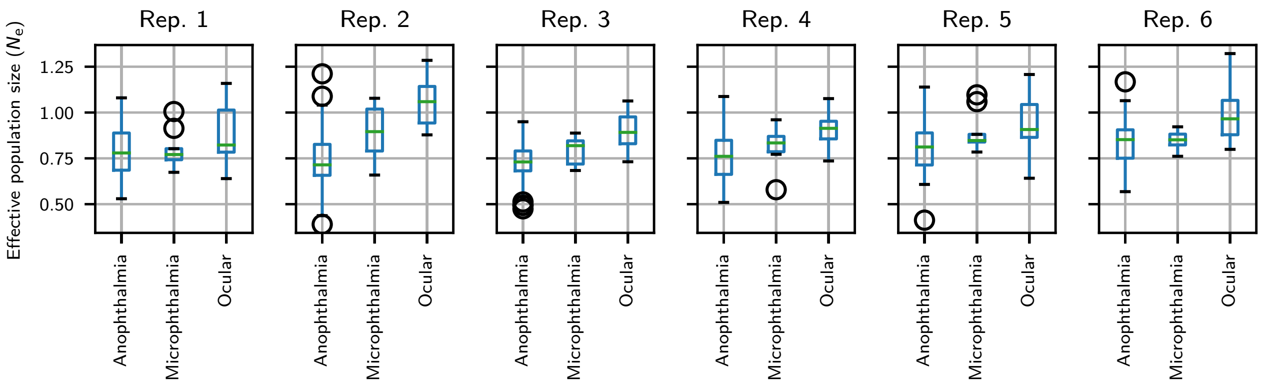

Figure 28: N_e as a function of ocular structure in isopods

```
Analysis of Variance Table

Response: PopulationSize
                  Df Sum Sq Mean Sq F value    Pr(>F)
Ocular.structure   2 1.9335 0.96676  53.316 < 2.2e-16 ***
Rep                5 0.4226 0.08452   4.661  0.000382 ***
Residuals        388 7.0355 0.01813
---
Signif. codes:  0 ‘***’ 0.001 ‘**’ 0.01 ‘*’ 0.05 ‘.’ 0.1 ‘ ’ 1
```

4.3 Identifiability of N_e and μ

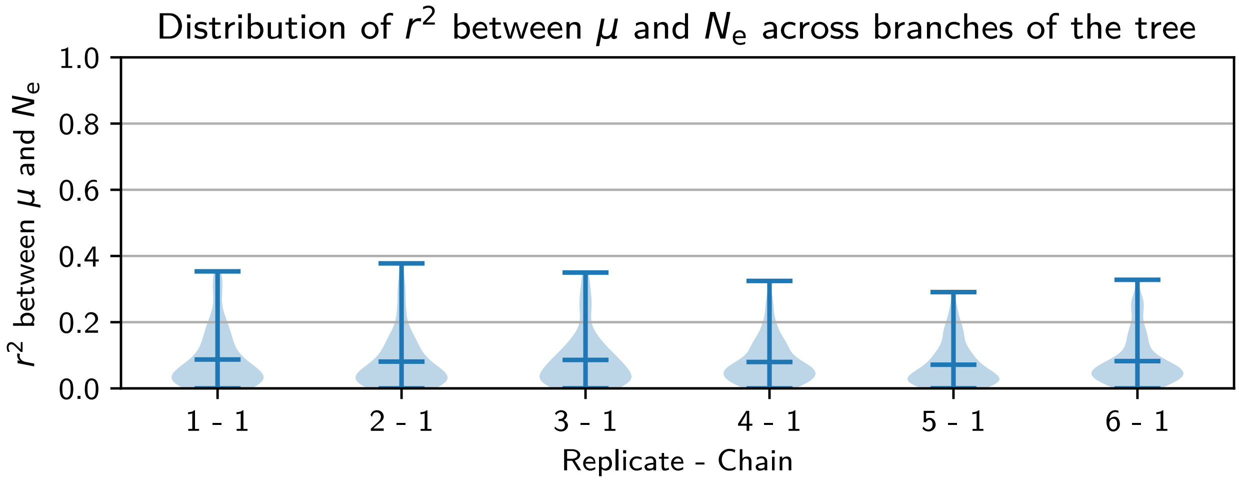

Figure 29: Some parameters might be strongly correlated or only weakly identifiable, which may make it hard to fit the model to realistically sized datasets. To test whether N_e and μ are identifiable, for each branch of the tree we draw a 2-D scatter plot for N_e and μ, where each point is a step in the MCMC procedure. We subsequently fit a linear regression and compute the coefficient of determination (r^2) for each branch of the tree. For a given MCMC, the distribution of r^2 across all branches is then represented as a violin plot. The distribution of r^2 is relatively low, below 0.1 on average, providing confidence that N_e and μ are indeed identifiable.

5 Empirical data in Primates

5.1 Chain convergence

Obtained with the mechanistic inference model developed in this paper of site-specific amino-acid fitness profiles and log-Brownian process for N_e, μ and life-history traits.

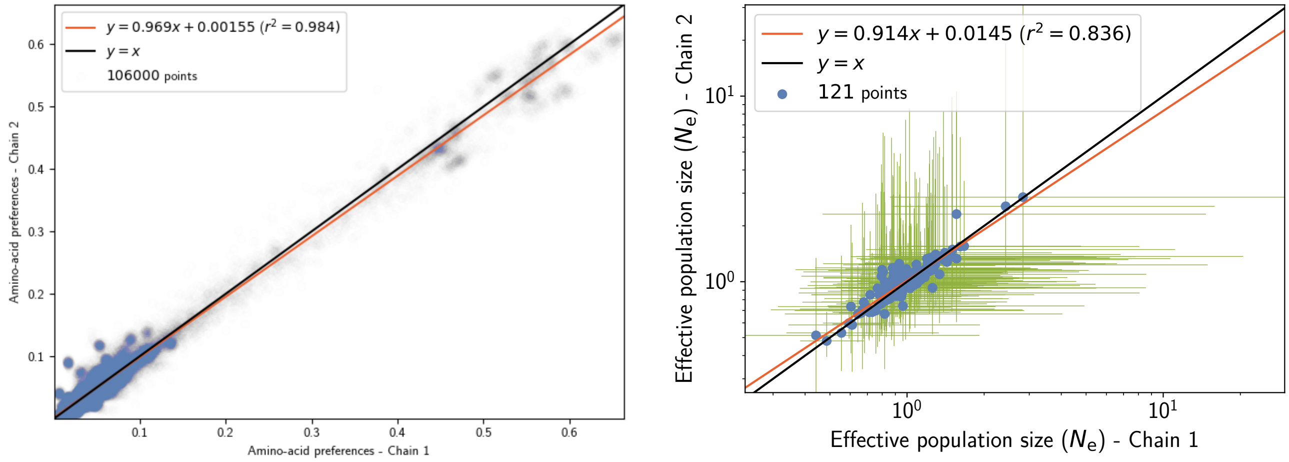

Figure 30: Chain convergence of site amino-acid preferences (left panel) and branch N_e (right panel).

5.2 Traits estimation (chain 1)

Obtained with the mechanistic inference model developed in this paper of site-specific amino-acid fitness profiles and log-Brownian process for N_e, μ and life-history traits.

**Correlation (ρ)**	N_e	μ	**maturity**	**mass**	**longevity**	π_S	π_N/π_S	**generation time**
N_e	-	-0.433^{**}	0.155	0.166	0.157	-0.133	0.104	0.16
μ	-	-	-0.792^{**}	-0.791^{**}	-0.773^{**}	0.62^{**}	-0.59	-0.78^{**}
**maturity**	-	-	-	0.986^{**}	0.985^{**}	-0.8^{**}	0.746	0.991^{**}
**mass**	-	-	-	-	0.977^{**}	-0.737^{**}	0.695	0.981^{**}
**longevity**	-	-	-	-	-	-0.819^{**}	0.752	0.999^{**}
π_S	-	-	-	-	-	-	-0.86^{**}	-0.816^{**}
π_N/π_S	-	-	-	-	-	-	-	0.752
**generation time**	-	-	-	-	-	-	-	-

Table 20: Correlation coefficient between effective population size (N_e), mutation rate per site per unit of time (μ), and life-history traits (maximum longevity, adult weight and female maturity) were computed in primates. Asterisks indicate strength of support ($^*pp > 0.95$, $^{**}pp > 0.975$).

**Covariance (Σ)**	N_e	μ	**maturity**	**mass**	**longevity**	π_S	π_N/π_S	**generation time**
N_e	1.08^{**}	-1.39^{**}	0.66	1.18	0.414	-0.251	0.0898	0.452
μ	-	9.86^{**}	-10.1^{**}	-17.5^{**}	-6.44^{**}	3.42^{**}	-1.28	-6.96^{**}
**maturity**	-	-	16.9^{**}	28.4^{**}	10.6^{**}	-5.39^{**}	1.9	11.5^{**}
**mass**	-	-	-	49.8^{**}	18.1^{**}	-8.89^{**}	3.29	19.5^{**}
**longevity**	-	-	-	-	6.99^{**}	-3.75^{**}	1.31	7.47^{**}
π_S	-	-	-	-	-	3.26^{**}	-0.986^{**}	-3.96^{**}
π_N/π_S	-	-	-	-	-	-	0.419^{**}	1.39
**generation time**	-	-	-	-	-	-	-	8.02^{**}

Table 21: Correlation coefficient between effective population size (N_e), mutation rate per site per unit of time (μ), and life-history traits (maximum longevity, adult weight and female maturity) were computed in primates. Asterisks indicate strength of support ($^*pp > 0.95$, $^{**}pp > 0.975$).

**Partial coefficient**	N_e	μ	**maturity**	**mass**	**longevity**	π_S	π_N/π_S	**generation time**
N_e	-	-0.411	-0.0622	0.0184	-0.0436	-0.0482	-0.00476	0.0333
μ	-	-	0.0548	-0.101	0.146	-0.0134	-0.102	-0.124
**maturity**	-	-	-	0.292	-0.793^{**}	-0.167	0.0547	0.824^{**}
**mass**	-	-	-	-	-0.0589	0.43	-0.195	0.101
**longevity**	-	-	-	-	-	-0.159	-0.148	0.991^{**}
π_S	-	-	-	-	-	-	-0.573^{**}	0.11
π_N/π_S	-	-	-	-	-	-	-	0.144
**generation time**	-	-	-	-	-	-	-	-

Table 22: Partial correlation coefficient between Neffective population size (N_e), mutation rate per site per unit of time (μ), and life-history traits (maximum longevity, adult weight and female maturity) were computed in primates. Asterisks indicate strength of support ($^*pp > 0.95$, $^{**}pp > 0.975$).

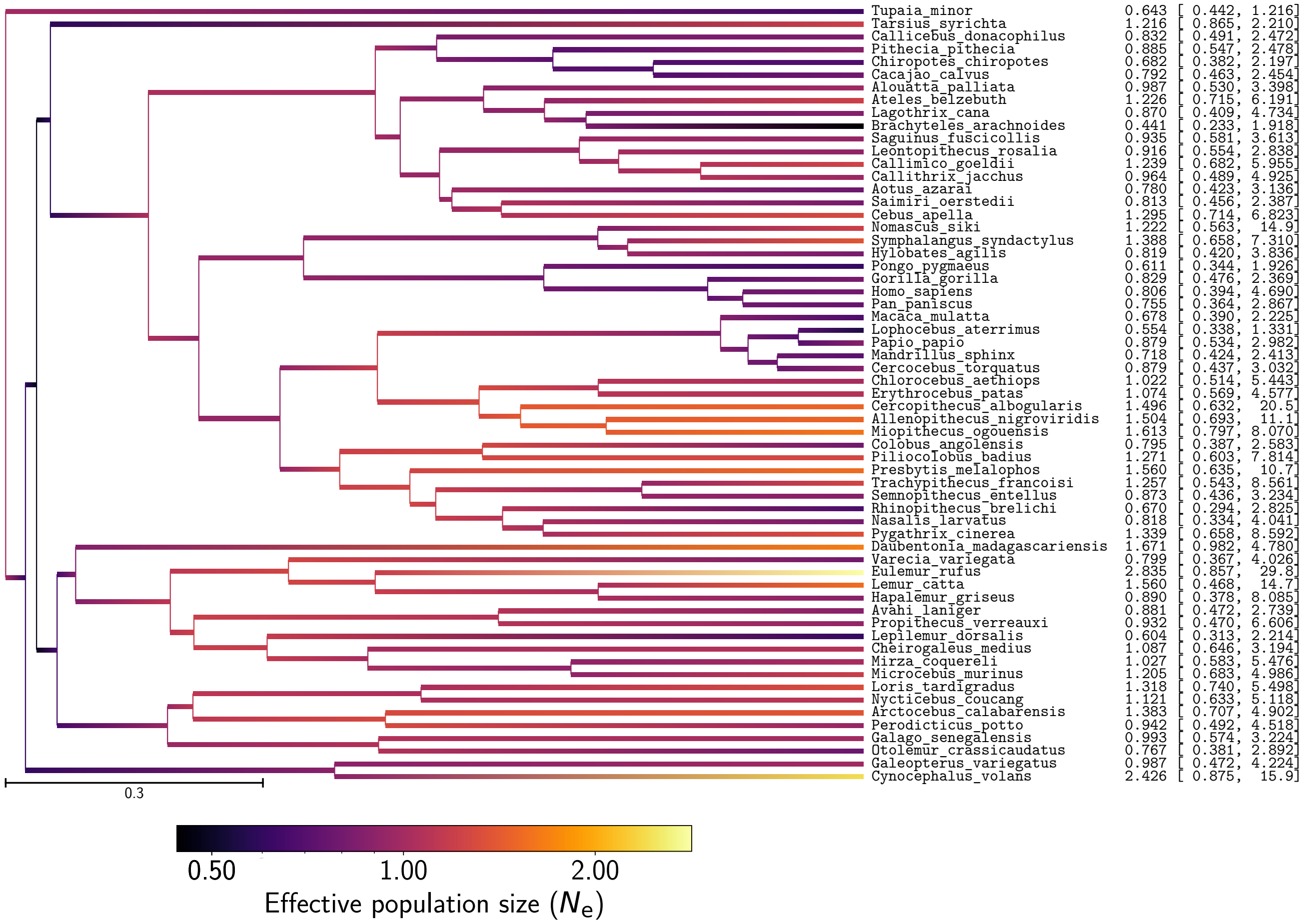

Figure 31: Effective population size (N_e) estimation in primates

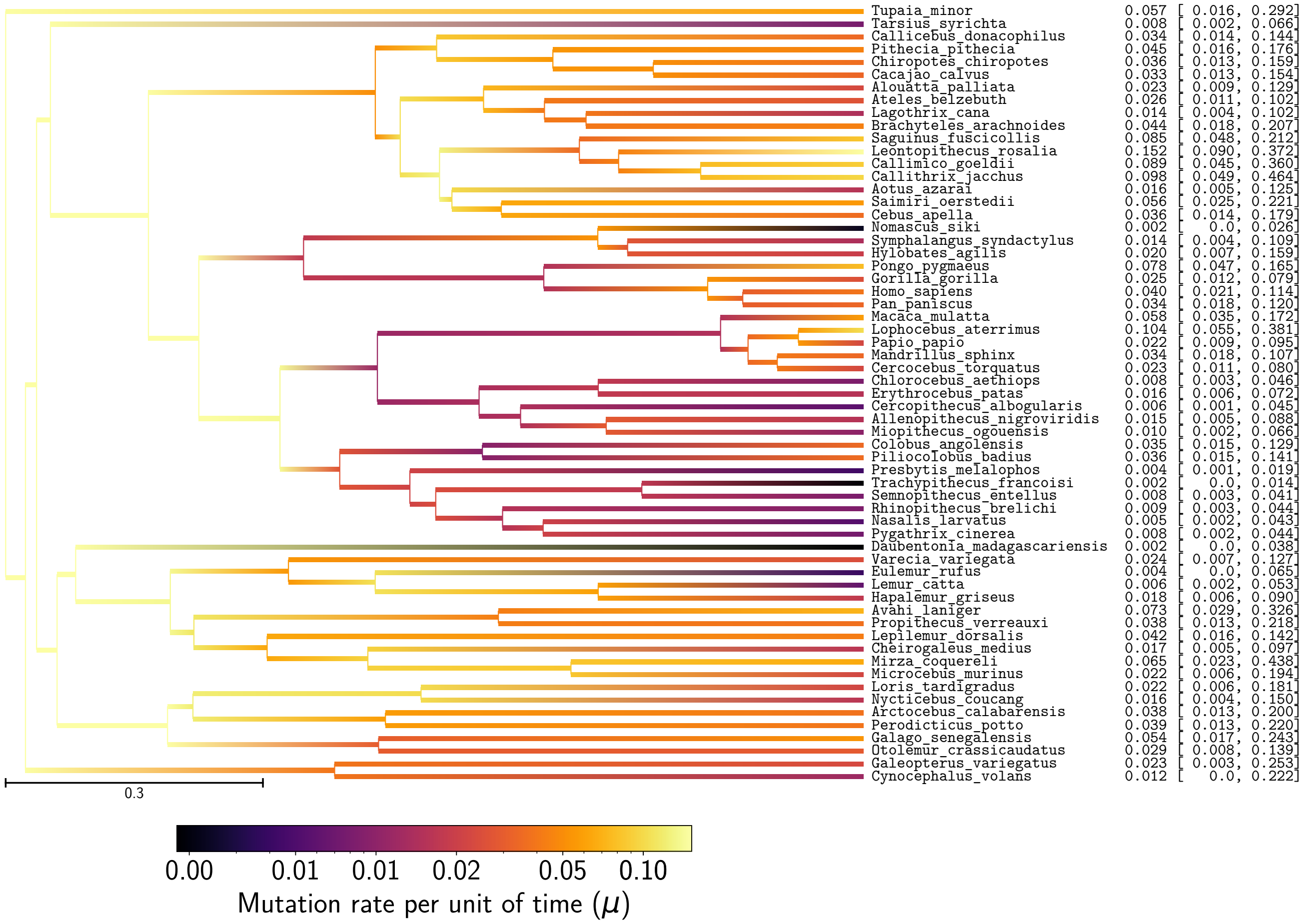

Figure 32: Mutation rate (μ) estimation in primates

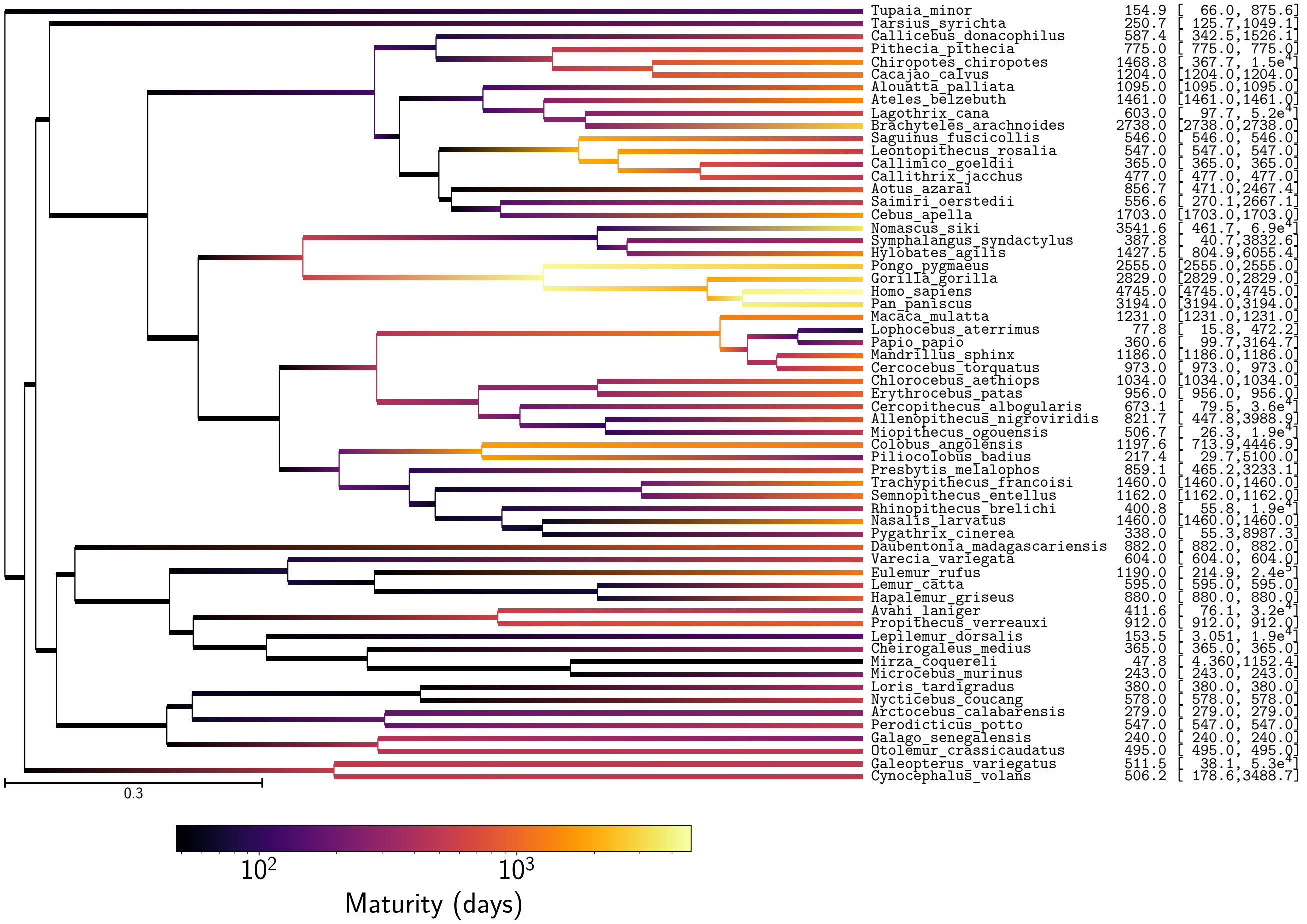

Figure 33: Female maturity estimation in primates

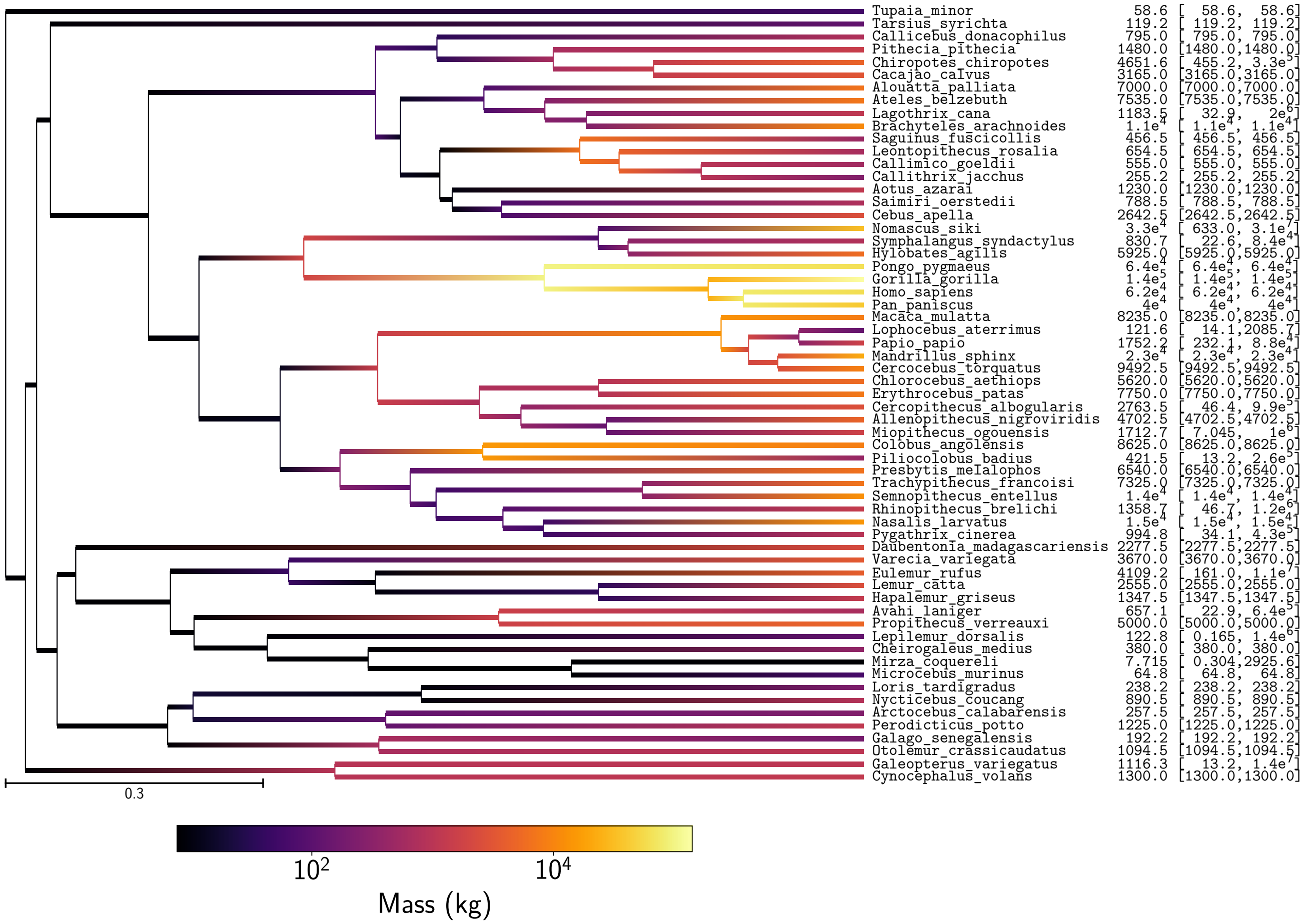

Figure 34: Mass estimation in primates

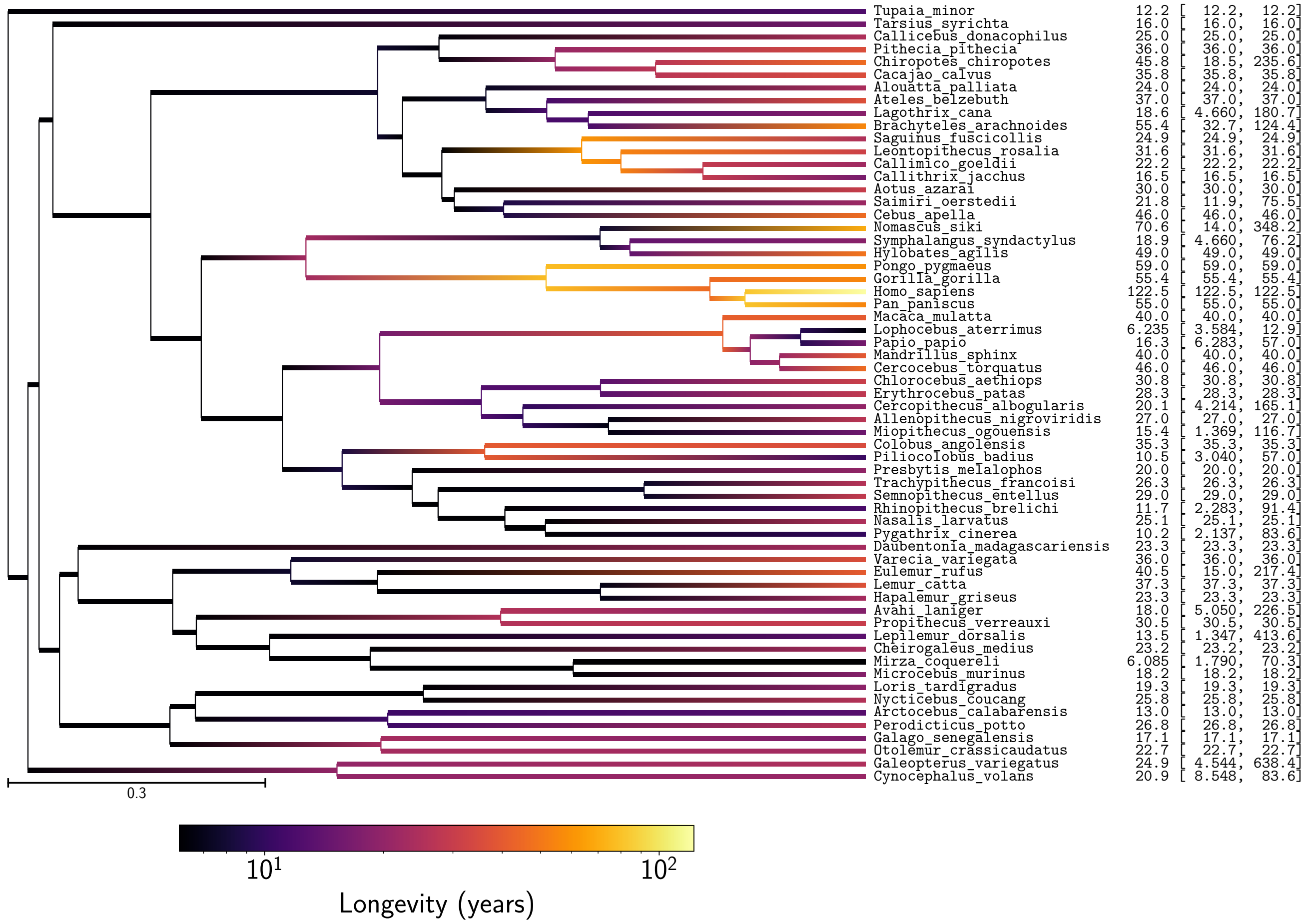

Figure 35: Longevity estimation in primates

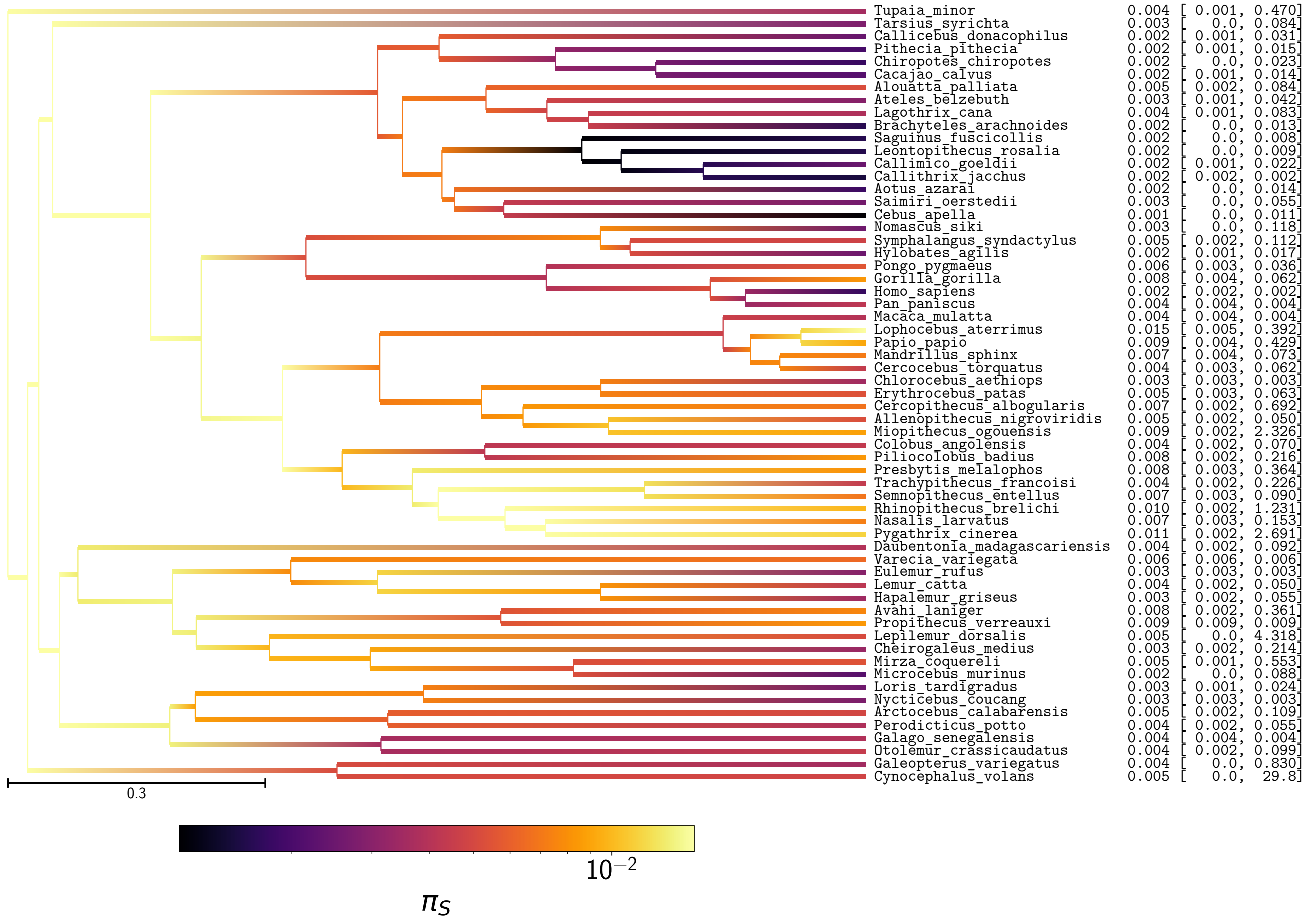

Figure 36: π_S estimation in primates

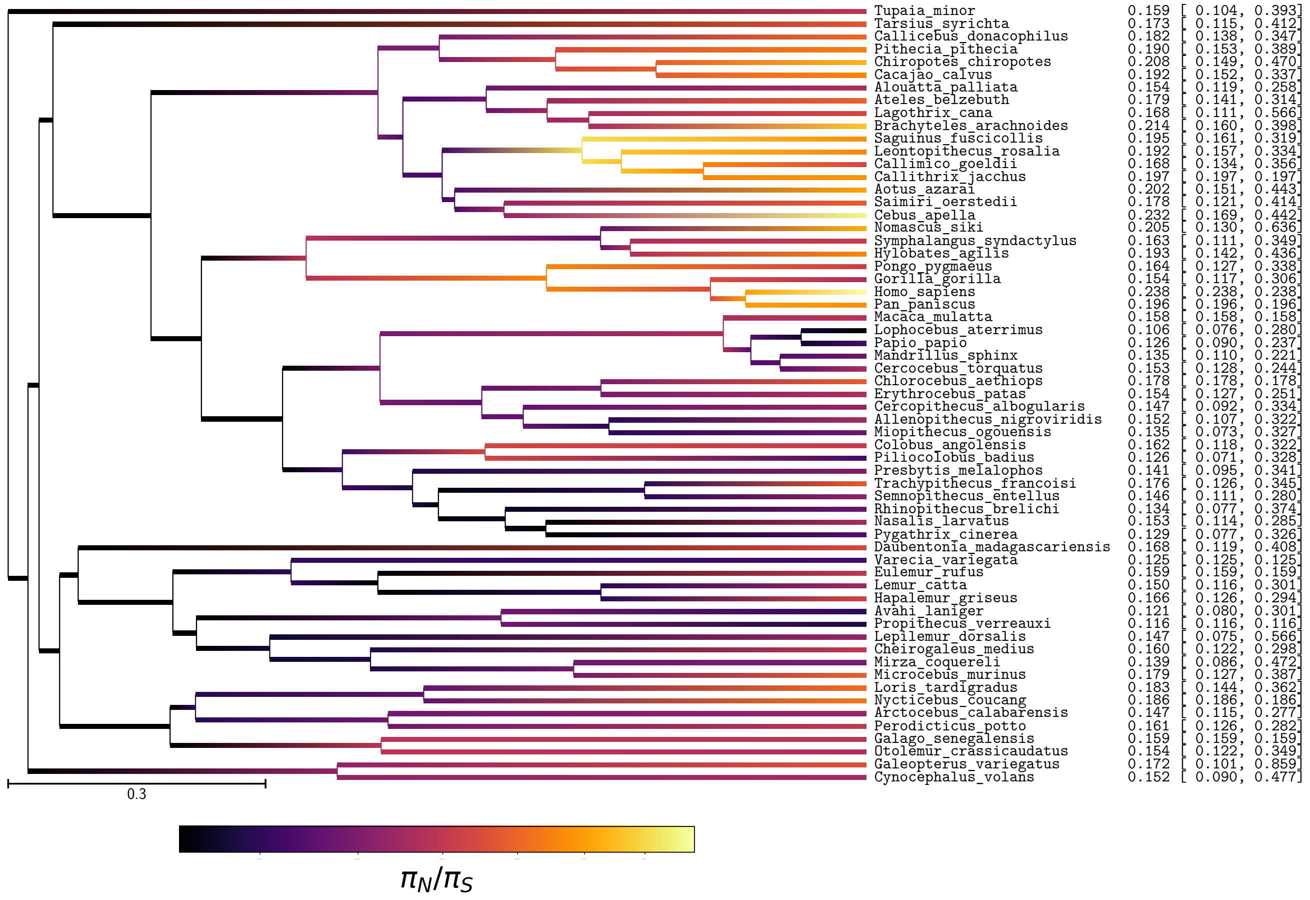

Figure 37: π_N/π_S estimation in primates

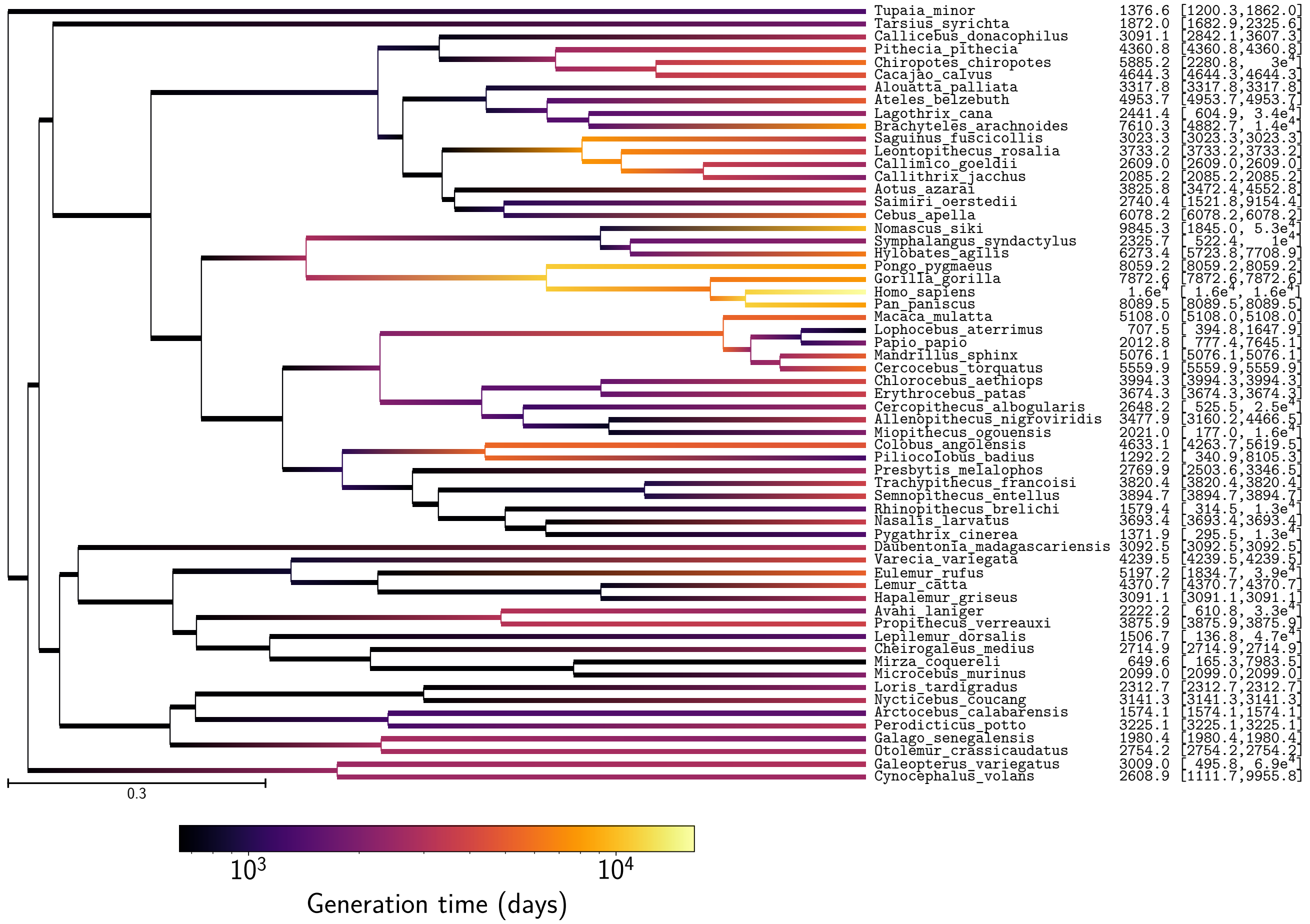

Figure 38: Generation time estimation in primates

5.3 Amino-acid preferences entropy

Experiment	$\langle\Omega\rangle$ (branch N_e)	$\langle\Omega\rangle$ (constant N_e)
Primates, chain 1	1.41 ± 0.10	1.49 ± 0.08
Primates, chain 2	1.40 ± 0.10	1.48 ± 0.08

Table 23: Estimated amino-acid entropy in primates. Obtained with the mechanistic inference model developed in this paper of site-specific amino-acid fitness profiles and log-Brownian process for N_e, μ and life-history traits (in the left column), or under the assumption of constant N_e (in the right column).

5.4 Traits estimation with branch ω (chain 1)

Obtained with the phenomenological inference model of log-Brownian process for the μ and the relative non-synonymous substitution rate (ω), as in Lartillot and Poujol (2011).

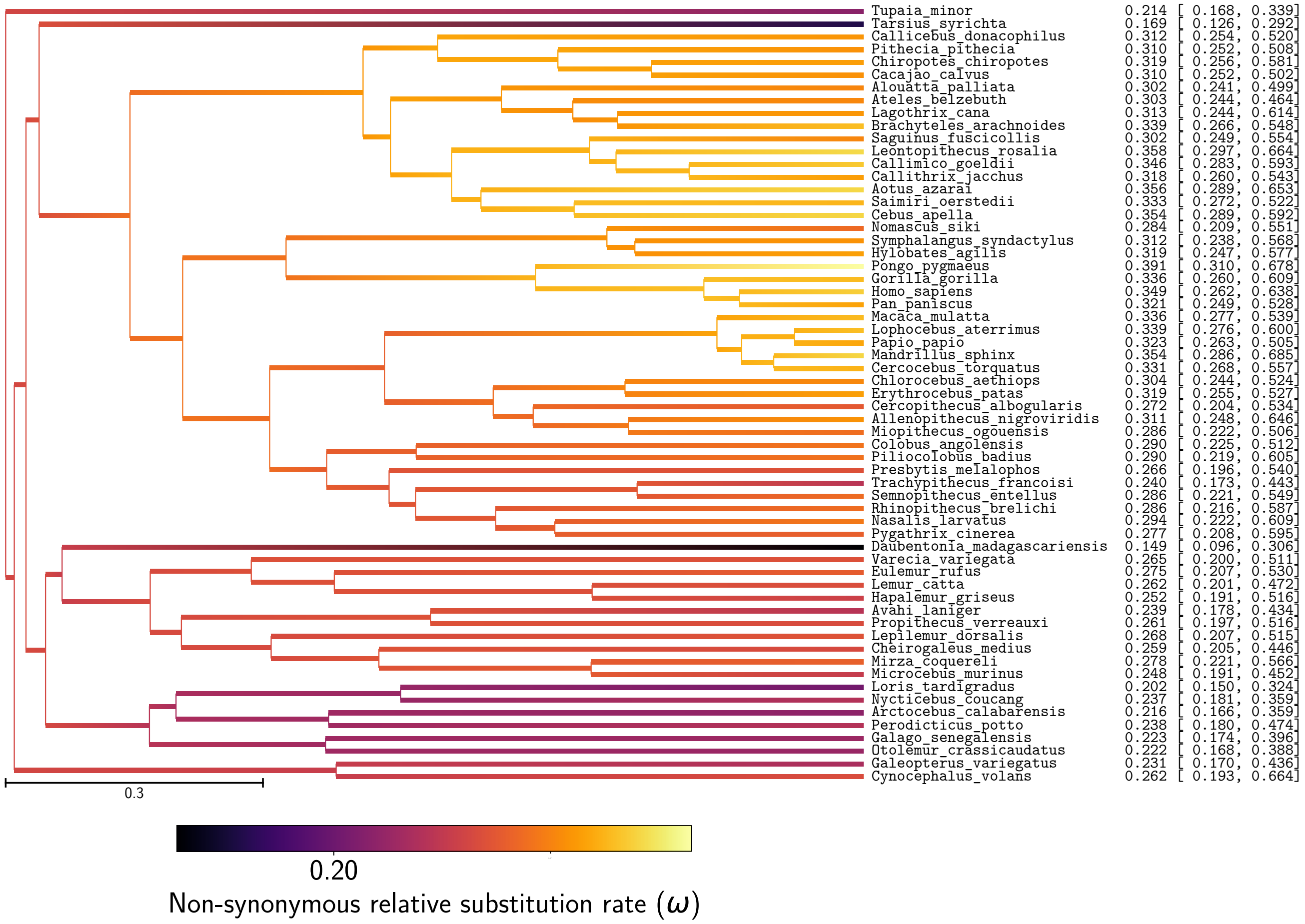

Figure 39: Non-synonymous substitution rate (ω) estimation in primates

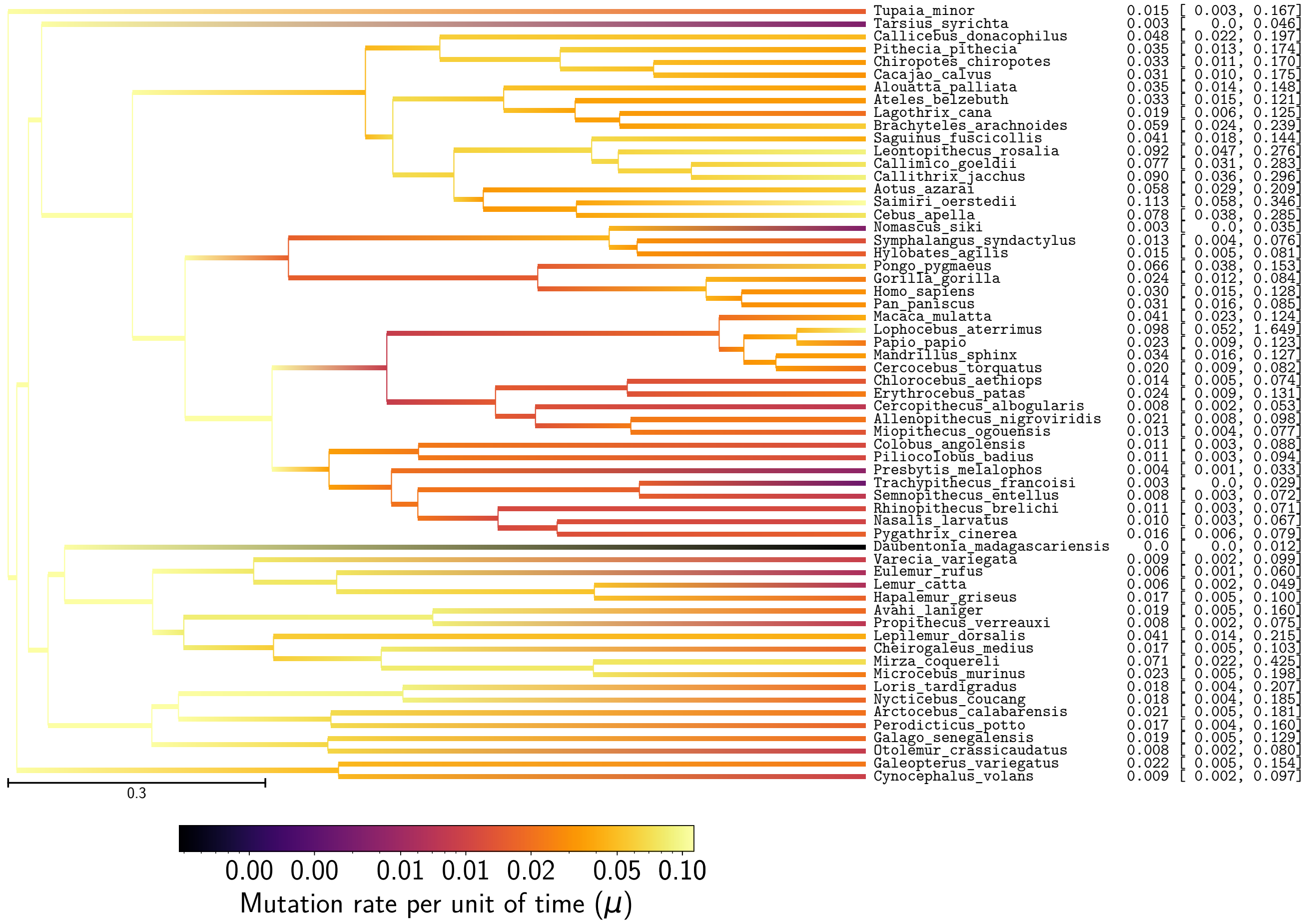

Figure 40: Mutation rate (μ) estimation in primates

**Correlation (ρ)**	ω	μ	**maturity**	**mass**	**longevity**	π_S	π_N/π_S	**generation time**
ω	-	0.294	0.000316	0.0361	0.0155	-0.197	0.145	0.0111
μ	-	-	-0.804^{**}	-0.798^{**}	-0.817^{**}	-0.0201	0.031	-0.823^{**}
**maturity**	-	-	-	0.952^{**}	0.957^{**}	-0.166	0.162	0.97^{**}
**mass**	-	-	-	-	0.933^{**}	-0.0437	0.0427	0.943^{**}
**longevity**	-	-	-	-	-	-0.223	0.165	0.999^{**}
π_S	-	-	-	-	-	-	-0.664	-0.212
π_N/π_S	-	-	-	-	-	-	-	0.162
**generation time**	-	-	-	-	-	-	-	-

Table 24: Correlation coefficient between non-synonymous substitution rate (ω), mutation rate per site per unit of time (μ), and life-history traits (maximum longevity, adult weight and female maturity) were computed in primates. Asterisks indicate strength of support ($^*pp > 0.95$, $^{**}pp > 0.975$).

Covariance (Σ)	ω	μ	maturity	mass	longevity	π_S	π_N/π_S	generation time
ω	0.0674^{**}	0.231	−0.0106	0.0149	−0.00138	−0.0435	0.0101	−0.00314
μ	-	8.71^{**}	-4.8^{**}	-9.22^{**}	-3.97^{**}	0.188	0.0483	-4.08^{**}
maturity	-	-	4.95^{**}	8.37^{**}	3.29^{**}	−1.01	0.000924	3.53^{**}
mass	-	-	-	16.3^{**}	6.14^{**}	−0.932	−0.0741	6.45^{**}
longevity	-	-	-	-	2.76^{**}	−0.577	0.0919	2.82^{**}
π_S	-	-	-	-	-	1.3^{**}	−0.148	−0.637
π_N/π_S	-	-	-	-	-	-	0.182^{**}	0.0775
generation time	-	-	-	-	-	-	-	2.92^{**}

Table 25: Correlation coefficient between non-synonymous substitution rate (ω), mutation rate per site per unit of time (μ), and life-history traits (maximum longevity, adult weight and female maturity) were computed in primates. Asterisks indicate strength of support ($^*pp > 0.95$, $^{**}pp > 0.975$).

Partial coefficient	ω	μ	maturity	mass	longevity	π_S	π_N/π_S	generation time
ω	-	0.463	−0.0461	0.248	−0.027	−0.193	−0.0681	0.0319
μ	-	-	0.0649	−0.000258	0.0374	−0.128	0.115	−0.075
maturity	-	-	-	0.228	-0.834^{**}	−0.0991	0.0491	0.854^{**}
mass	-	-	-	-	−0.038	0.435	−0.123	0.0851
longevity	-	-	-	-	-	−0.184	−0.145	0.994^{**}
π_S	-	-	-	-	-	-	-0.553^{*}	0.125
π_N/π_S	-	-	-	-	-	-	-	0.136
generation time	-	-	-	-	-	-	-	-

Table 26: Partial correlation coefficient between non-synonymous substitution rate (ω), mutation rate per site per unit of time (μ), and life-history traits (maximum longevity, adult weight and female maturity) were computed in primates. Asterisks indicate strength of support ($^*pp > 0.95$, $^{**}pp > 0.975$).

6 Sufficient statistics

A sequence of length Z evolves by point substitutions, according to a random process defined by the substitution matrices $\boldsymbol{Q}^{(\mathrm{b,z})}$, over a phylogenetic tree. A realization of the random process along a branch b, and at a particular site z results in a detailed substitution history, which will be denoted by $\mathcal{H}^{(\mathrm{b,z})}$.

6.1 Path sufficient statistics

All sites owning to the same category of fitness profile share the same substitution rate matrix. Hence, $\mathcal{H}^{(\mathrm{b,z})}$ can be gathered across all sites owing to a specific category k, denoted $\mathcal{H}^{(\mathrm{b})}$. If we express the probability of the substitution mapping ($\mathcal{H}^{(\mathrm{b,k})}$) as a function of the codon substitution process for this category k, we get the following expression:

$$\mathbb{P}(\mathcal{H}^{(\mathrm{b,k})}|l^{(\mathrm{b})},\boldsymbol{Q}^{(\mathrm{b,k})}) \propto \left[\prod_{i=1}^{61}\left[\pi_i^{(\mathrm{b,k})}\right]^{n_i^{(\mathrm{b,k})}}\right]\cdot\left[\prod_{1\leq i,j\leq 61}\left[Q_{i,j}^{(\mathrm{b,k})}\right]^{m_{i,j}^{(\mathrm{b,k})}}\right]\cdot\left[\prod_{i=1}^{61}\mathrm{e}^{-\left|Q_{i,i}^{(\mathrm{b,k})}\right|a_i^{(\mathrm{b,k})}}\right], \tag{26}$$

where we define the sufficient statistics:

- $m_{i,j}^{(\mathrm{b,k})}$ is the total number of substitutions from codon i to codon j
- $n_i^{(\mathrm{b,k})}$ is the number of sites starting with codon i at the tip of the branch.
- $a_i^{(\mathrm{b,k})}$ is the total waiting time in codon i.

Once these sufficient statistics have been computed, the parameters of the substitution matrix $\boldsymbol{Q}^{(\mathrm{b,k})}$ can be resampled conditional on $\mathcal{H}^{(\mathrm{b,k})}$, using equation 26 each time the likelihood needs to be recomputed. This leads to relatively fast MCMC strategy.

6.2 Length sufficient statistics

$\mathcal{H}^{(\mathrm{b,z})}$ can also be gathered across all sites along a specific branch, giving $\mathcal{H}^{(\mathrm{b})}$. Then the probability of the substitution history given the branch lengths ($l^{(\mathrm{b})} = \mu^{(\mathrm{b})}\Delta T^{(\mathrm{b})}$), takes a very simple form:

$$\mathbb{P}(\mathcal{H}^{(\mathrm{b})}|L^{(\mathrm{b})}) \propto \left[L^{(\mathrm{b})}\right]^{u^{(\mathrm{b})}} \mathrm{e}^{-r^{(\mathrm{b})}L^{(\mathrm{b})}}, \tag{27}$$

where we define the sufficient statistics:

- $u^{(\mathrm{b})}$ is the total number of substitutions over branch b, summed over all sites.
- $r^{(\mathrm{b})}$ is the mean rate away from current codon state (averaged over the entire substitution history).

Thus, formally, the probability of the substitution mapping can be summarized by saying that the total number of substitutions along a given branch over all sites, $u^{(\mathrm{b})}$, is Poisson distributed, of mean $r^{(\mathrm{b})}L^{(\mathrm{b})}$.

6.3 Scatter sufficient statistics

From the independent contrast $\boldsymbol{C}^{(\mathrm{b})}$ of the Brownian process $\boldsymbol{B}^{(\mathrm{n})}$, we can define the 2×2 scatter sufficient statistic matrix, $\boldsymbol{A}$ as:

$$\boldsymbol{A} = \sum_{b=1}^{2P-2} \boldsymbol{C}^{(\mathrm{b})} \cdot \left[\boldsymbol{C}^{(\mathrm{b})}\right]^{\mathrm{T}} \tag{28}$$

By Bayes theorem, the posterior on $\boldsymbol{\Sigma}$, conditional on a particular realization of B (and thus of $\boldsymbol{C}$) is an invert Wishart distribution, of parameter $\kappa\boldsymbol{I} + \boldsymbol{A}$ and with $2P - 2 + 3$ degrees of freedom.

$$\boldsymbol{\Sigma} \sim \mathrm{Wishart}^{-1}\left(\kappa\boldsymbol{I} + \boldsymbol{A}, 2P - 2 + 3\right) \tag{29}$$

This invert Wishart distribution can be obtained by sampling $2P - 2 + 3$ independent and identically distributed multivariate normal random variables $\boldsymbol{Z}^{(a)}$ defined by

$$\boldsymbol{Z}^{(a)} \sim \mathcal{N}\left(\boldsymbol{0}, \left[\kappa\boldsymbol{I} + \boldsymbol{A}\right]^{-1}\right). \tag{30}$$

And from these multivariate samples, $\boldsymbol{\Sigma}$ is Gibbs sampled as:

$$\boldsymbol{\Sigma} = \left(\sum_{k=1}^{2P-2+3} \boldsymbol{Z}^{(a)} \cdot \left[\boldsymbol{Z}^{(a)}\right]^{\mathrm{T}}\right)^{-1} \tag{31}$$

References

Blanquart, F. and Bataillon, T. 2016. Epistasis and the structure of fitness landscapes: Are experimental fitness landscapes compatible with fisher's geometric model? *Genetics*, 203(2): 847–862.

Bloom, J. D. 2017. Identification of positive selection in genes is greatly improved by using experimentally informed site-specific models. *Biology Direct*, 12(1): 1–24.

De Magalhães, J. P. and Costa, J. 2009. A database of vertebrate longevity records and their relation to other life-history traits. *Journal of Evolutionary Biology*, 22(8): 1770–1774.

Gillespie, D. T. 1977. Exact stochastic simulation of coupled chemical reactions. *The Journal of Physical Chemistry*, 81(25): 2340–2361.

Goldstein, R. A. and Pollock, D. D. 2017. Sequence entropy of folding and the absolute rate of amino acid substitutions. *Nature Ecology & Evolution*, 1(12): 1923–1930.

Lartillot, N. and Poujol, R. 2011. A phylogenetic model for investigating correlated evolution of substitution rates and continuous phenotypic characters. *Molecular Biology and Evolution*, 28(1): 729–744.

Miyazawa, S. and Jernigan, R. L. 1985. Estimation of effective interresidue contact energies from protein crystal structures: quasi-chemical approximation. *Macromolecules*, 18(3): 534–552.

Ranwez, V., Delsuc, F., Ranwez, S., Belkhir, K., Tilak, M. K., and Douzery, E. J. 2007. OrthoMaM: A database of orthologous genomic markers for placental mammal phylogenetics. *BMC Evolutionary Biology*, 7(1): 1–12.

Scornavacca, C., Belkhir, K., Lopez, J., Dernat, R., Delsuc, F., Douzery, E. J., and Ranwez, V. 2019. OrthoMaM v10: Scaling-up orthologous coding sequence and exon alignments with more than one hundred mammalian genomes. *Molecular Biology and Evolution*, 36(4): 861–862.

Tacutu, R., Craig, T., Budovsky, A., Wuttke, D., Lehmann, G., Taranukha, D., Costa, J., Fraifeld, V. E., and De Magalhães, J. P. 2013. Human Ageing Genomic Resources: Integrated databases and tools for the biology and genetics of ageing. *Nucleic Acids Research*, 41(D1): D1027–D1033.

Tenaillon, O. 2014. The utility of Fisher's geometric model in evolutionary genetics. *Annual Review of Ecology, Evolution, and Systematics*, 45(1): 179–201.
